# Supplementary figures and images for: Adaptor linked K63 di-ubiquitin activates Nedd4/Rsp5 E3 ligase
Source: eLife. 2022 Jun 30;11:e77424. doi: 10.7554/eLife.77424 (PMC9282857; doi:10.7554/eLife.77424)

**B**

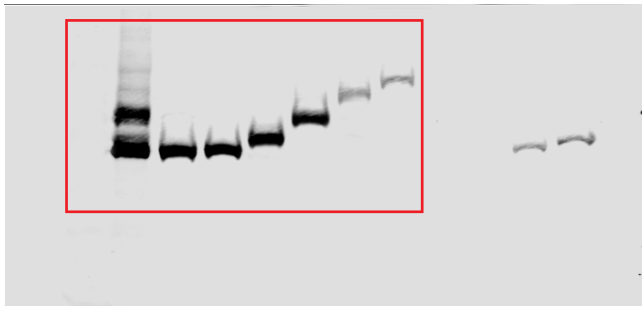

Blot: FLAG

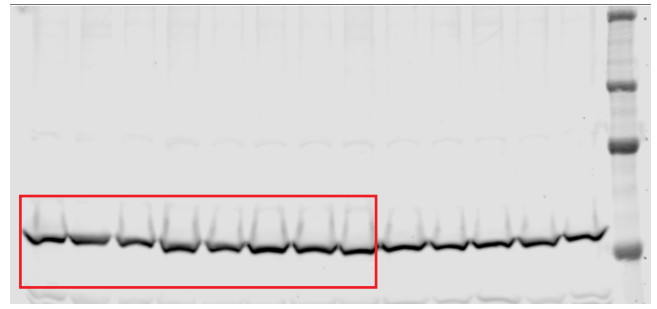

Blot: G6PDH

**C**

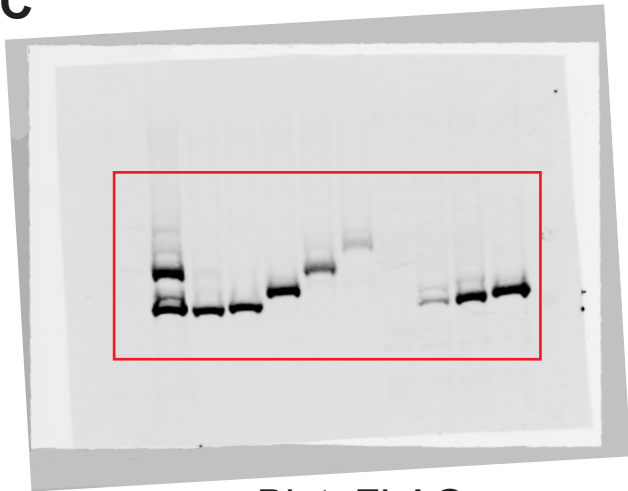

Blot: FLAG

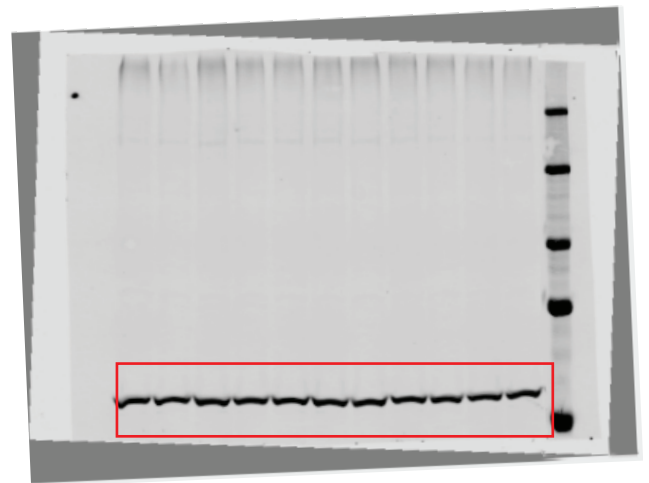

Blot: G6PDH

**Figure 1**

Supplement: Figure 1—source data 1. [file elife-77424-fig1-data1.pdf]

**B**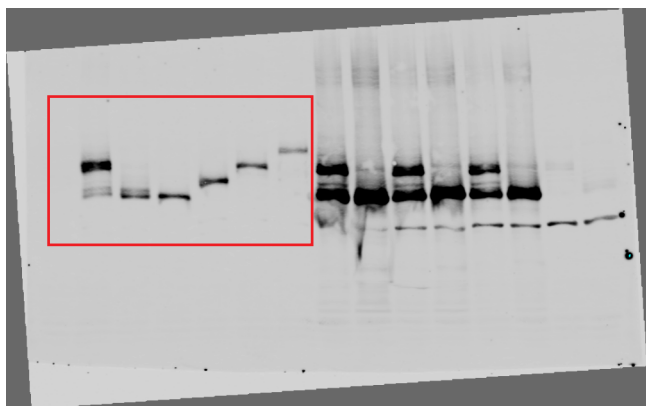

Blot: FLAG

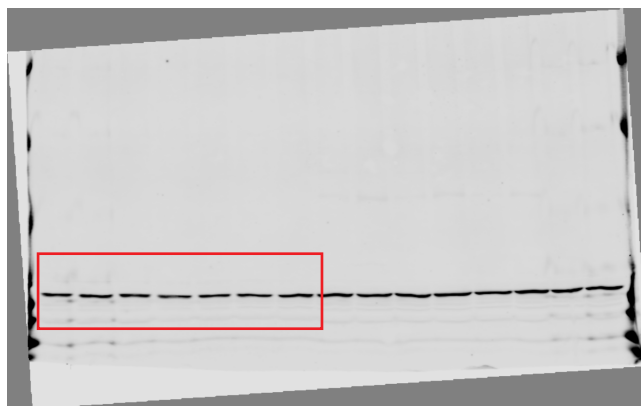

Blot: G6PDH

**C**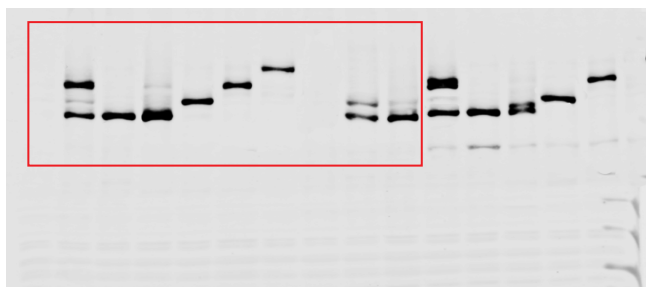

Blot: FLAG

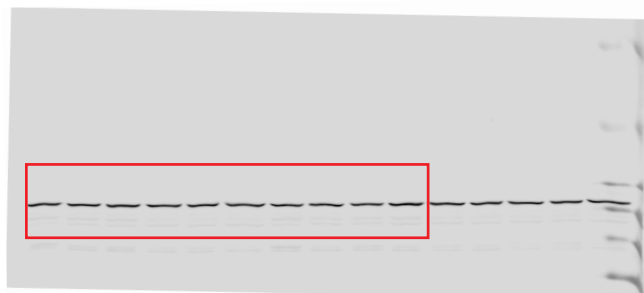

Blot: G6PDH

**Figure 1-figure supplement 1**

Supplement: Figure 1—figure supplement 1—source data 1. [file elife-77424-fig1-figsupp1-data1.pdf]

**1B**

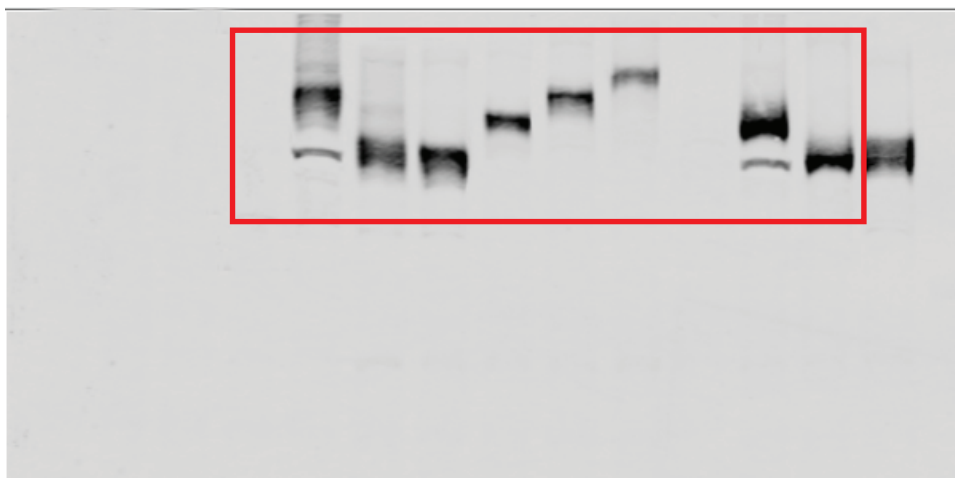

(Blot: FLAG)

**1B**

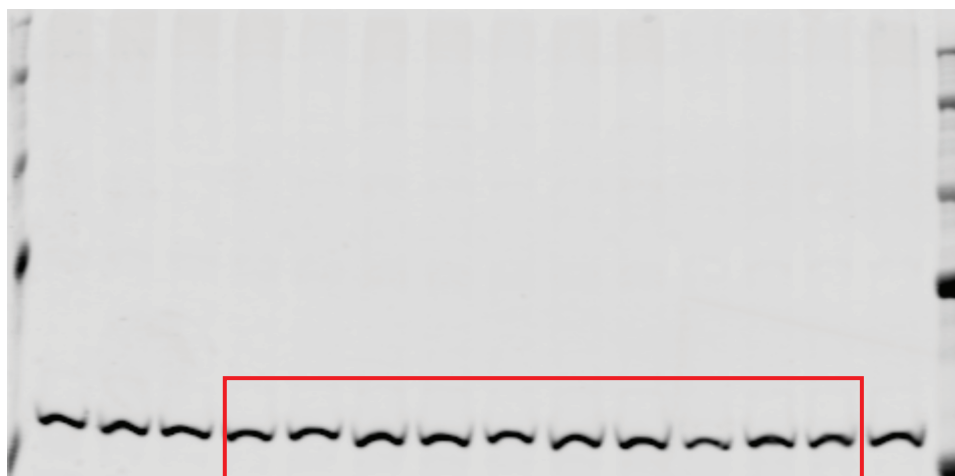

(Blot: G6PDH)

**Figure 1-figure supplement 2**

Supplement: Figure 1—figure supplement 2—source data 1. [file elife-77424-fig1-figsupp2-data1.pdf]

**A**

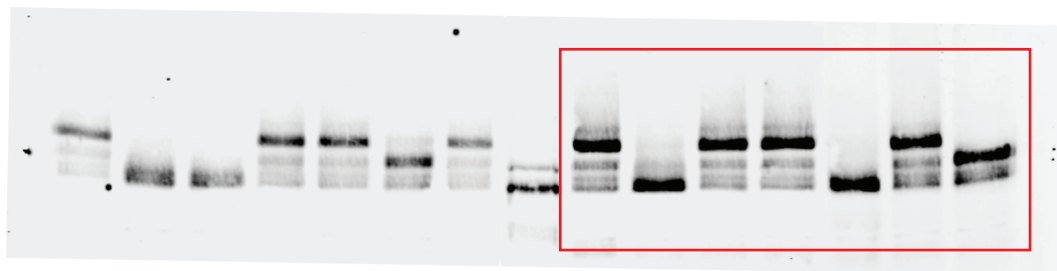

(Blot: FLAG)

**B**

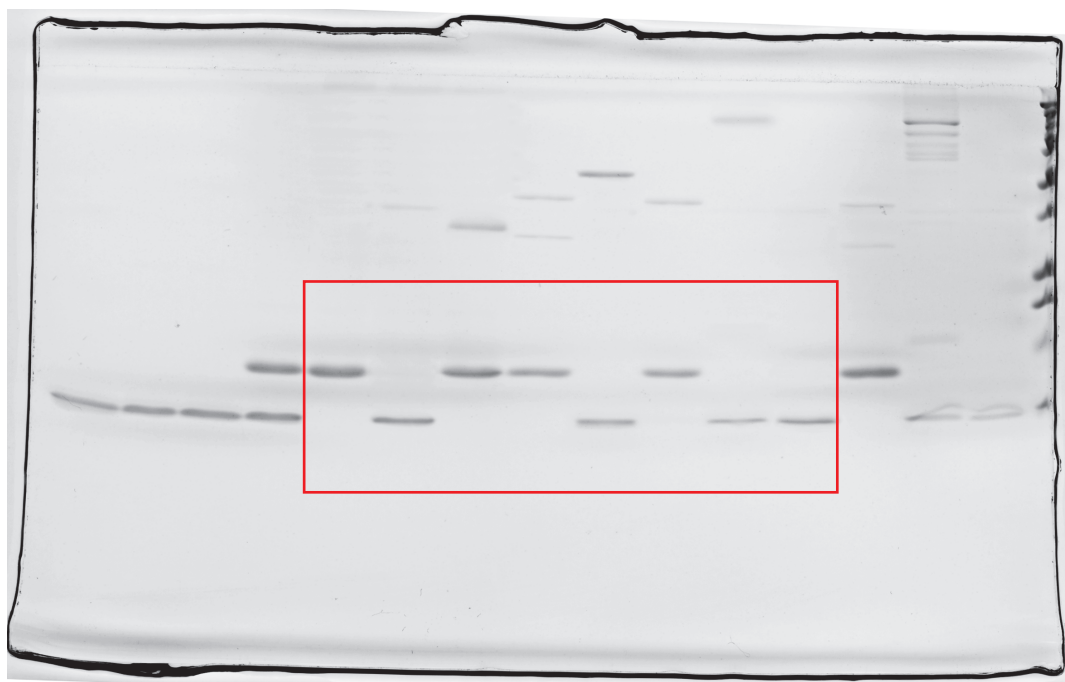

(Coomassie stain)

**Figure 1-figure supplement 3**

Supplement: Figure 1—figure supplement 3—source data 1. [file elife-77424-fig1-figsupp3-data1.pdf]

**A**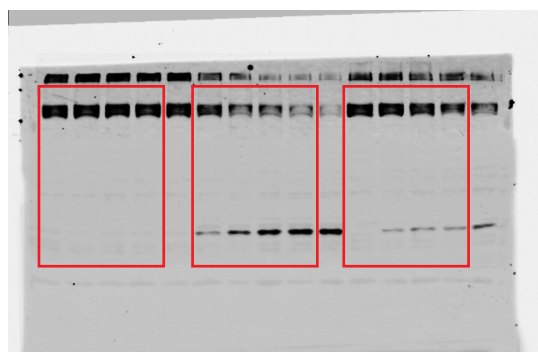

Blot: GFP

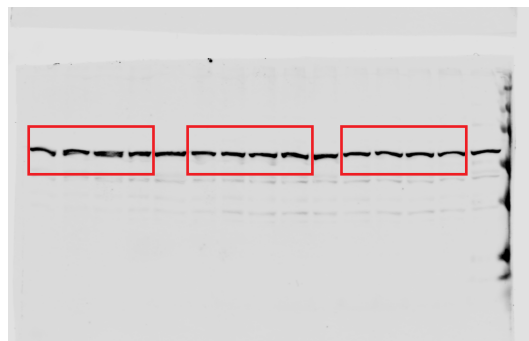

Blot: G6PDH

**D**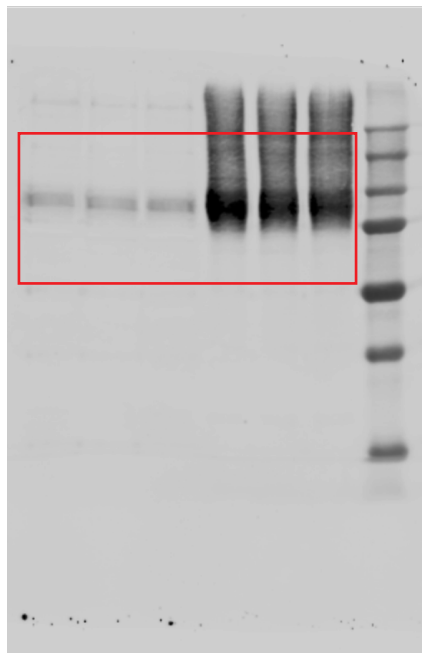

Blot: GFP

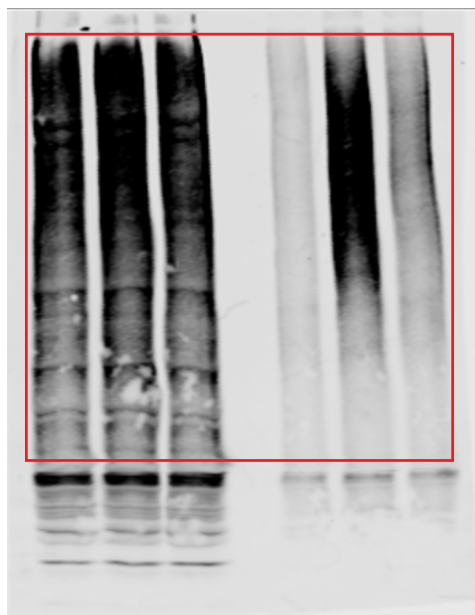

Blot: Myc

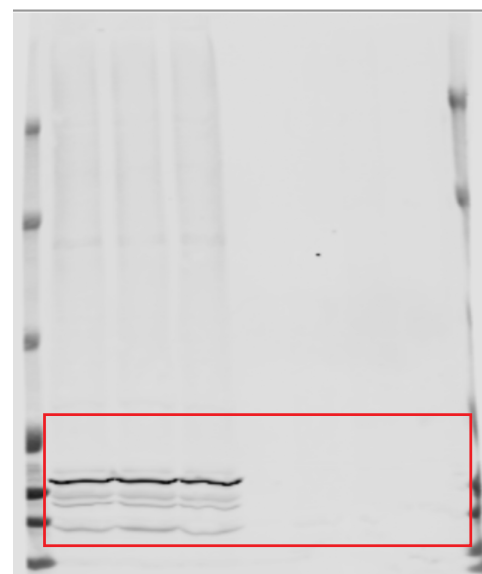

Blot: G6PDH

**E**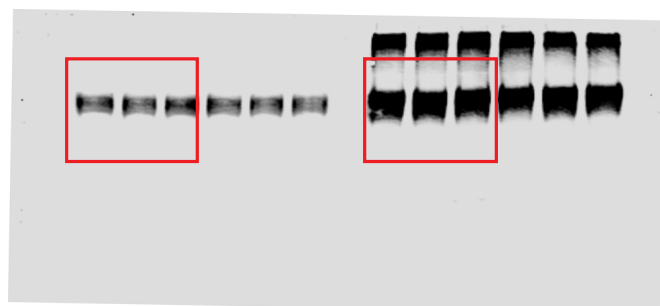

Blot: GFP

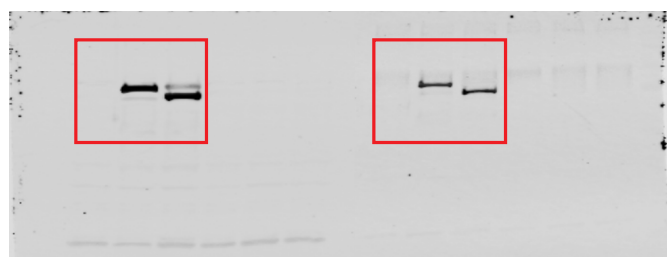

Blot: FLAG

**Figure 2**

Supplement: Figure 2—source data 1. [file elife-77424-fig2-data1.pdf]

**A**

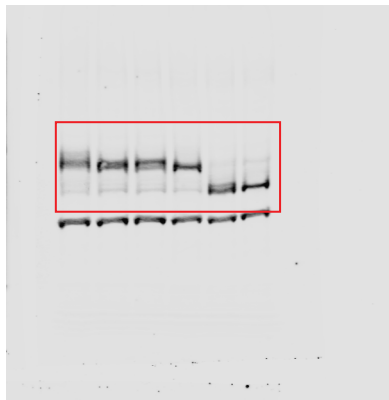

Blot: FLAG

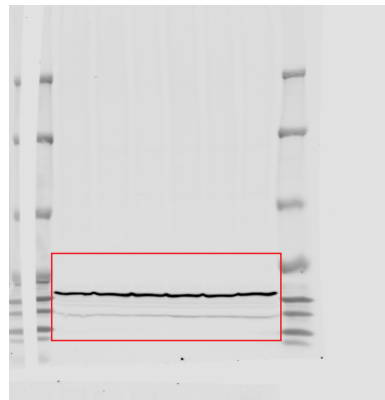

Blot: G6PDH

**B**

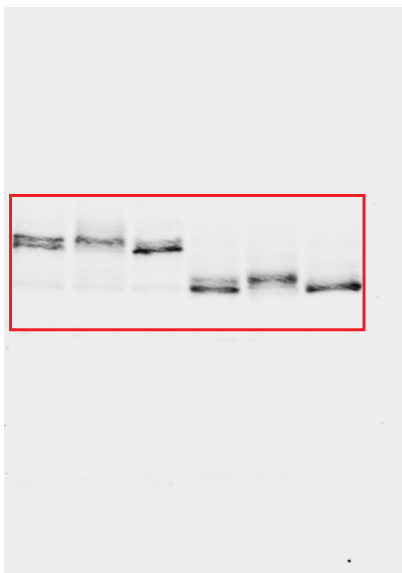

Blot: FLAG

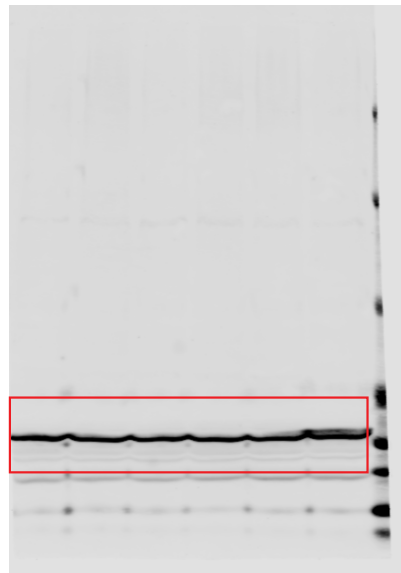

Blot: G6PDH

**Figure 2-figure supplement 2**

Supplement: Figure 2—figure supplement 2—source data 1. [file elife-77424-fig2-figsupp2-data1.pdf]

**A**

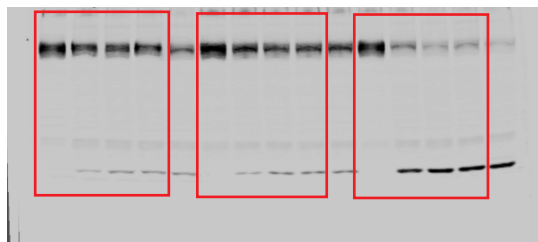

Blot: GFP

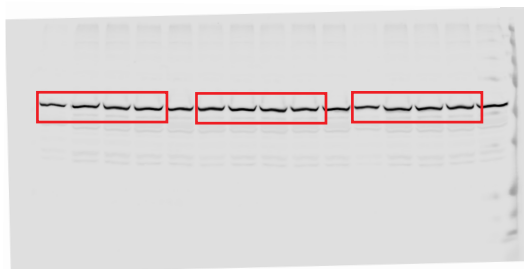

Blot: G6PDH

**C**

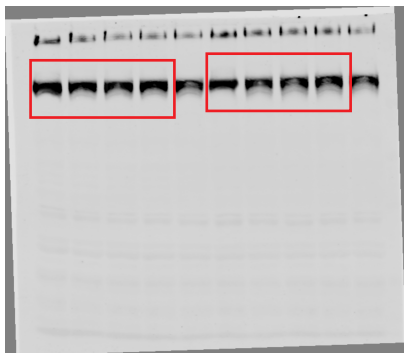

Blot: GFP

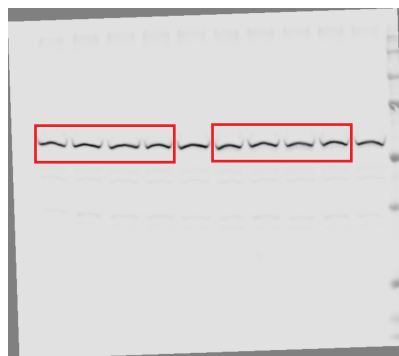

Blot: G6PDH

**Figure 2-figure supplement 3**

Supplement: Figure 2—figure supplement 3—source data 1. [file elife-77424-fig2-figsupp3-data1.pdf]

**A**

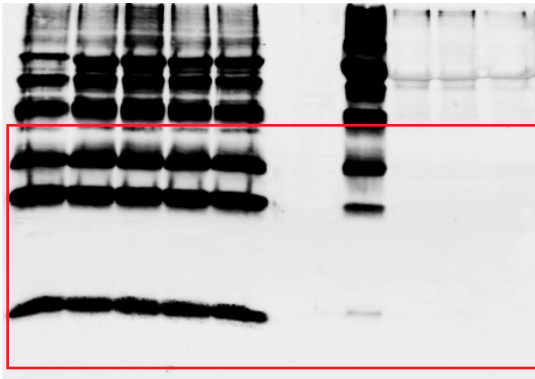

**Blot: 6xHis**

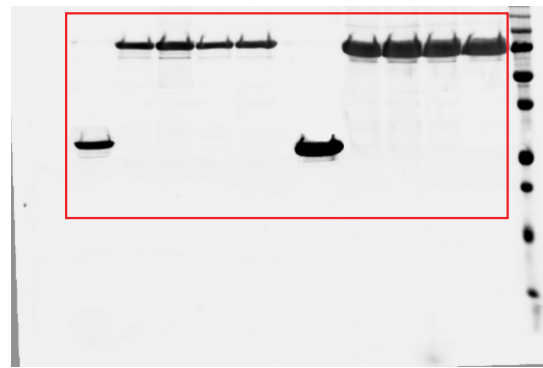

**Blot: GST**

**E**

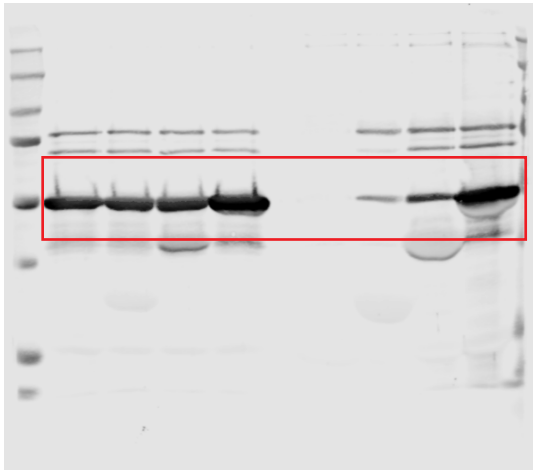

**Blot: Rsp5**

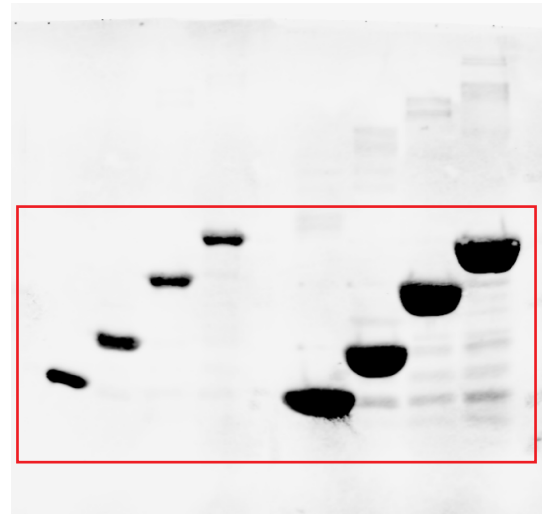

**Blot: GST**

**Figure 4**

Supplement: Figure 4—source data 1. [file elife-77424-fig4-data1.pdf]

**A**

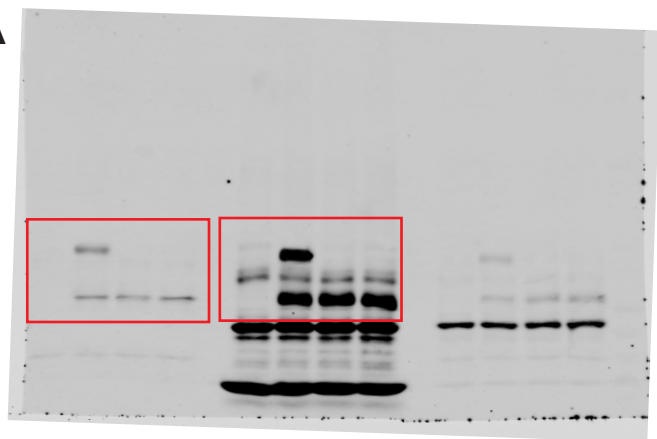

**Blot: FLAG**

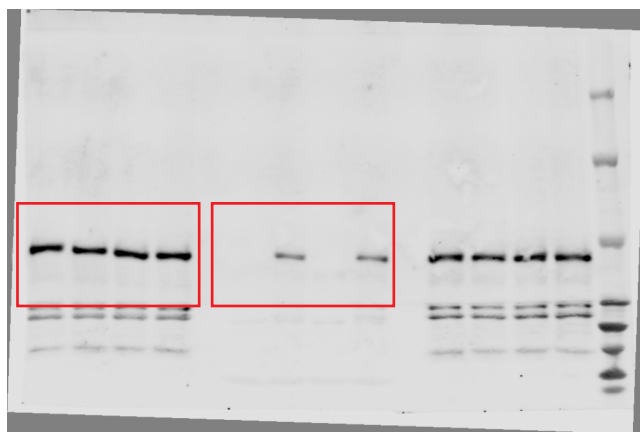

**Blot: Rsp5**

**B**

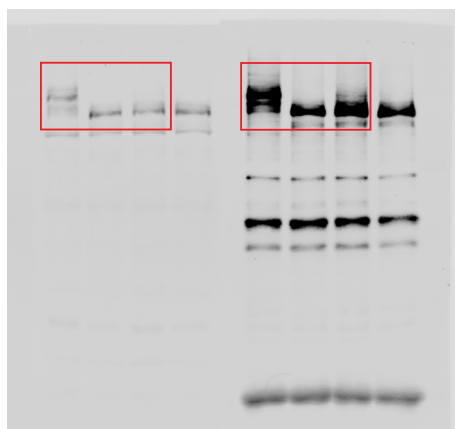

**Blot: FLAG**

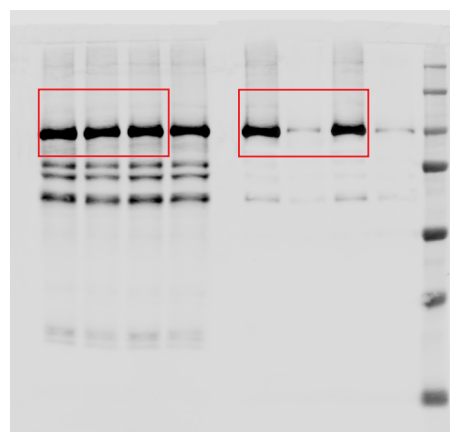

**Blot: Rsp5**

**Figure 5**

Supplement: Figure 5—source data 1. [file elife-77424-fig5-data1.pdf]

**A**

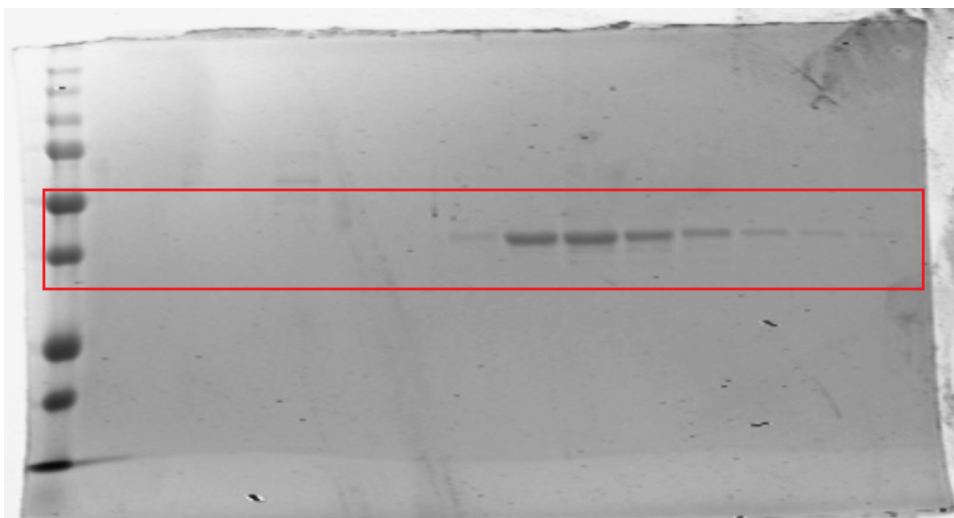

**B**

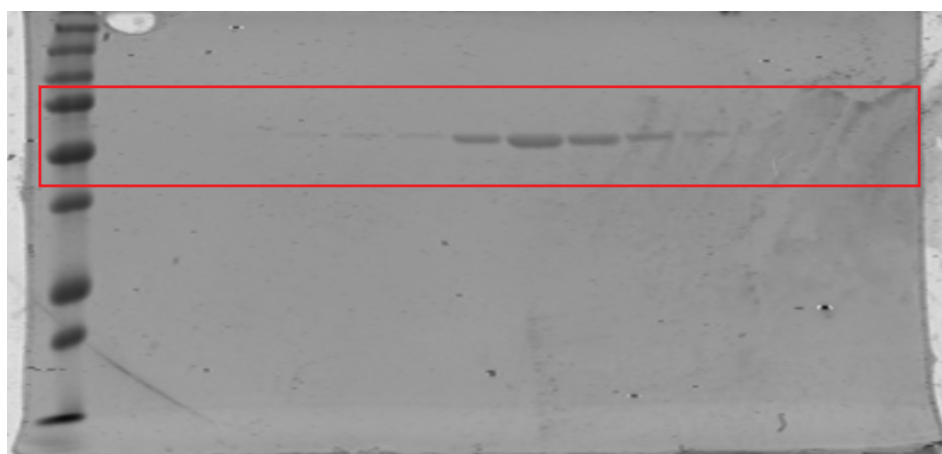

**C**

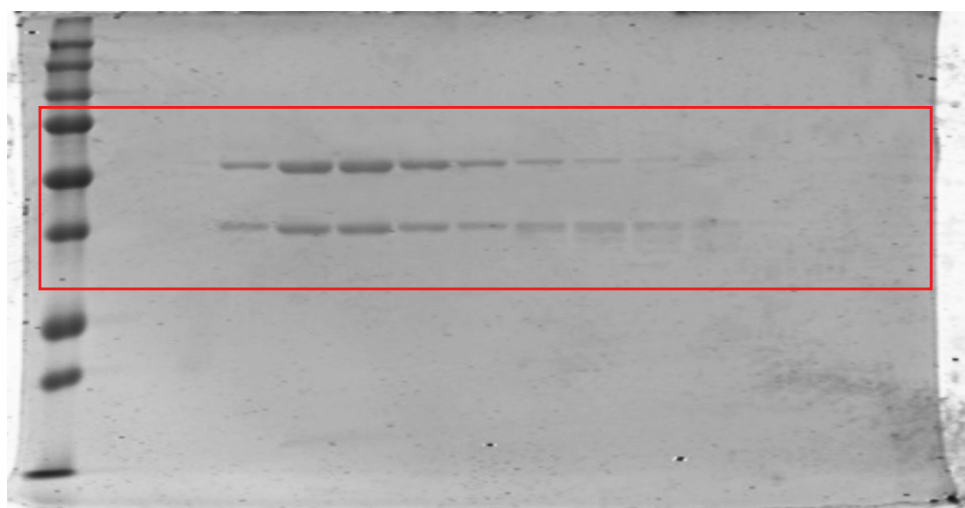

**Figure 5-figure supplement 2**

Supplement: Figure 5—figure supplement 2—source data 1. [file elife-77424-fig5-figsupp2-data1.pdf]

**A**

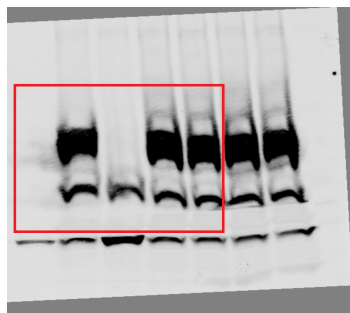

Blot: HA

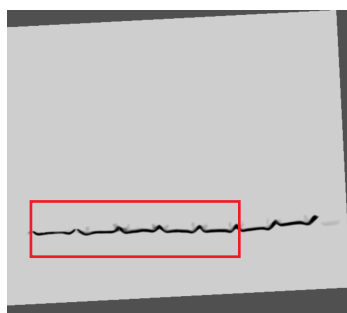

Blot: G6PDH

**B**

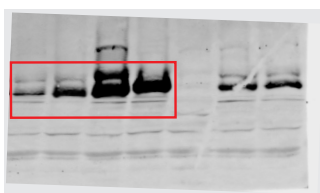

Blot: HA

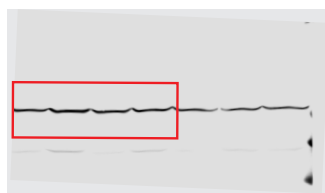

Blot: G6PDH

**C**

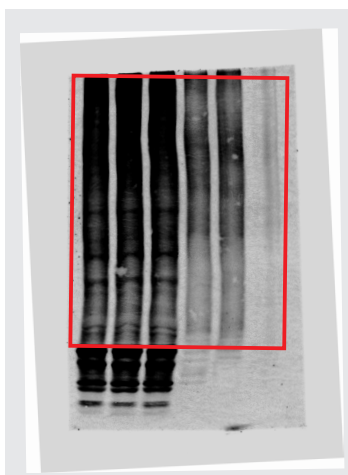

Blot: Myc

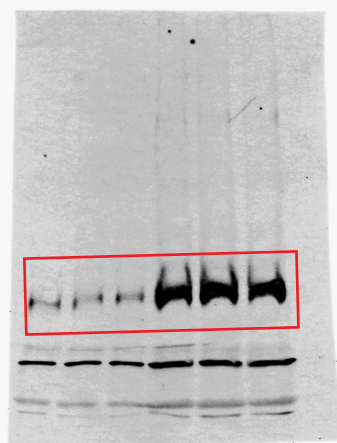

Blot: HA

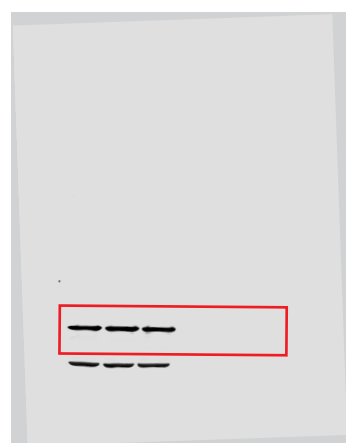

Blot: G6PDH

**D**

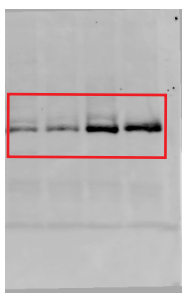

Blot: HA

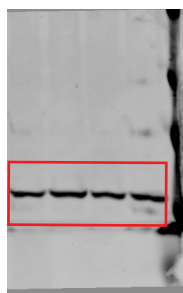

Blot: G6PDH

**Figure 6**

Supplement: Figure 6—source data 1. [file elife-77424-fig6-data1.pdf]

**A**

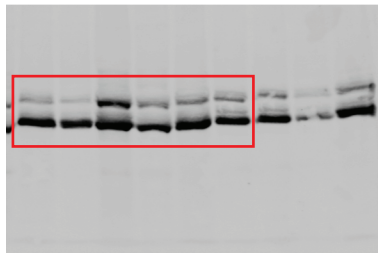

Blot: FLAG

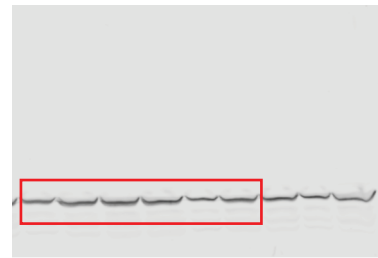

Blot: G6PDH

**B**

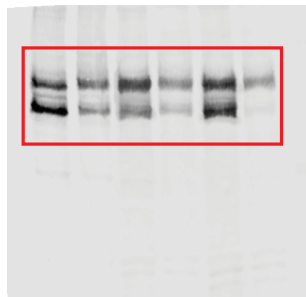

Blot: FLAG

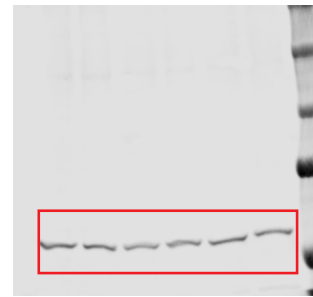

Blot: G6PDH

**Figure 6-figure supplement 2**

Supplement: Figure 6—figure supplement 2—source data 1. [file elife-77424-fig6-figsupp2-data1.pdf]

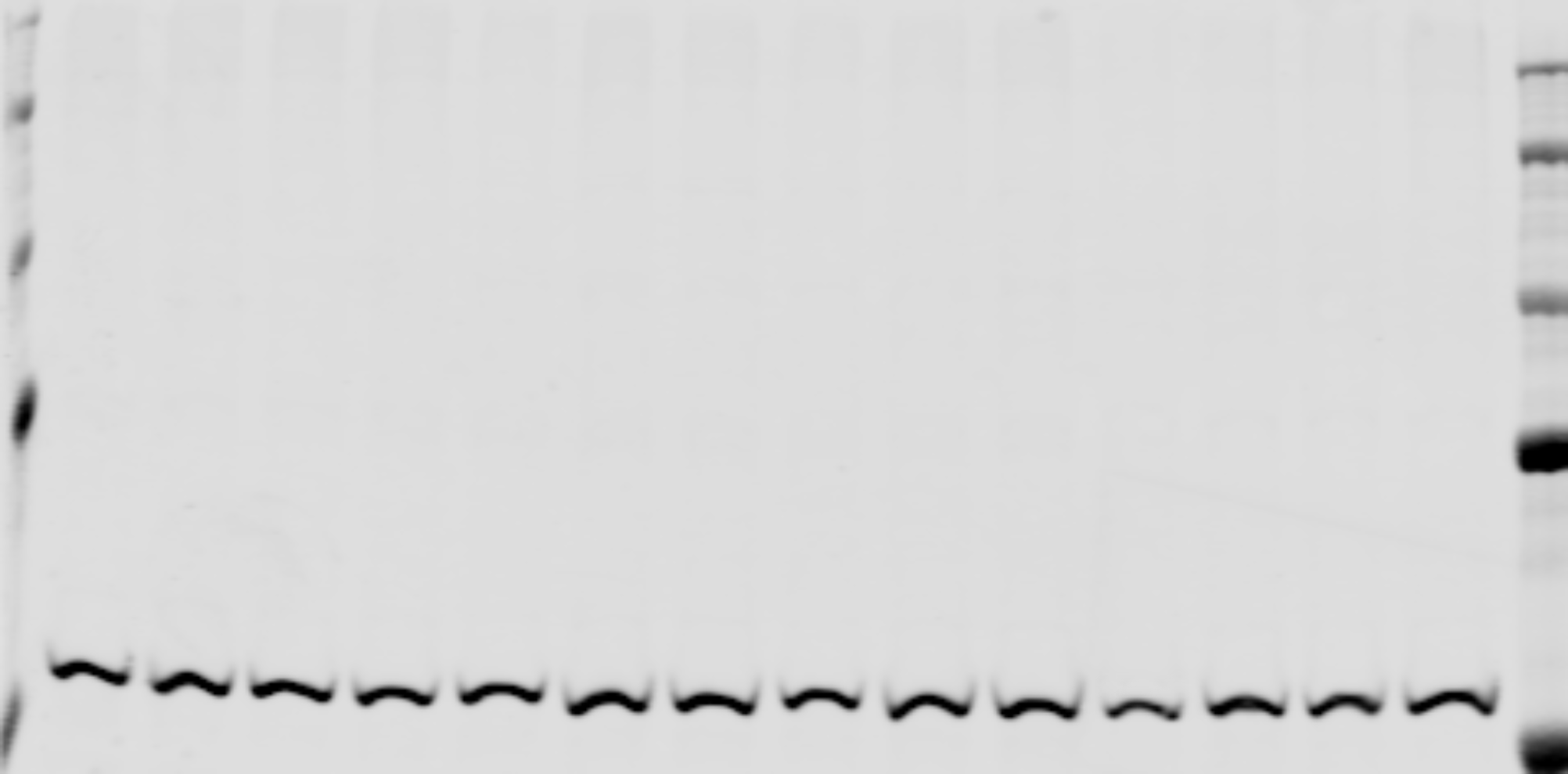

Supplement: Source data 1. [file elife-77424-data1.zip › Source data/Figure 1-figure supplemental 2/Figure 1-figure supplemental 2_G6PDH.tif]

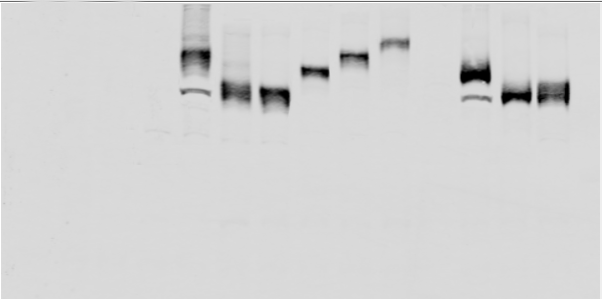

Supplement: Source data 1. [file elife-77424-data1.zip › Source data/Figure 1-figure supplemental 2/Figure 1-figure supplemental 2_FLAG.png]

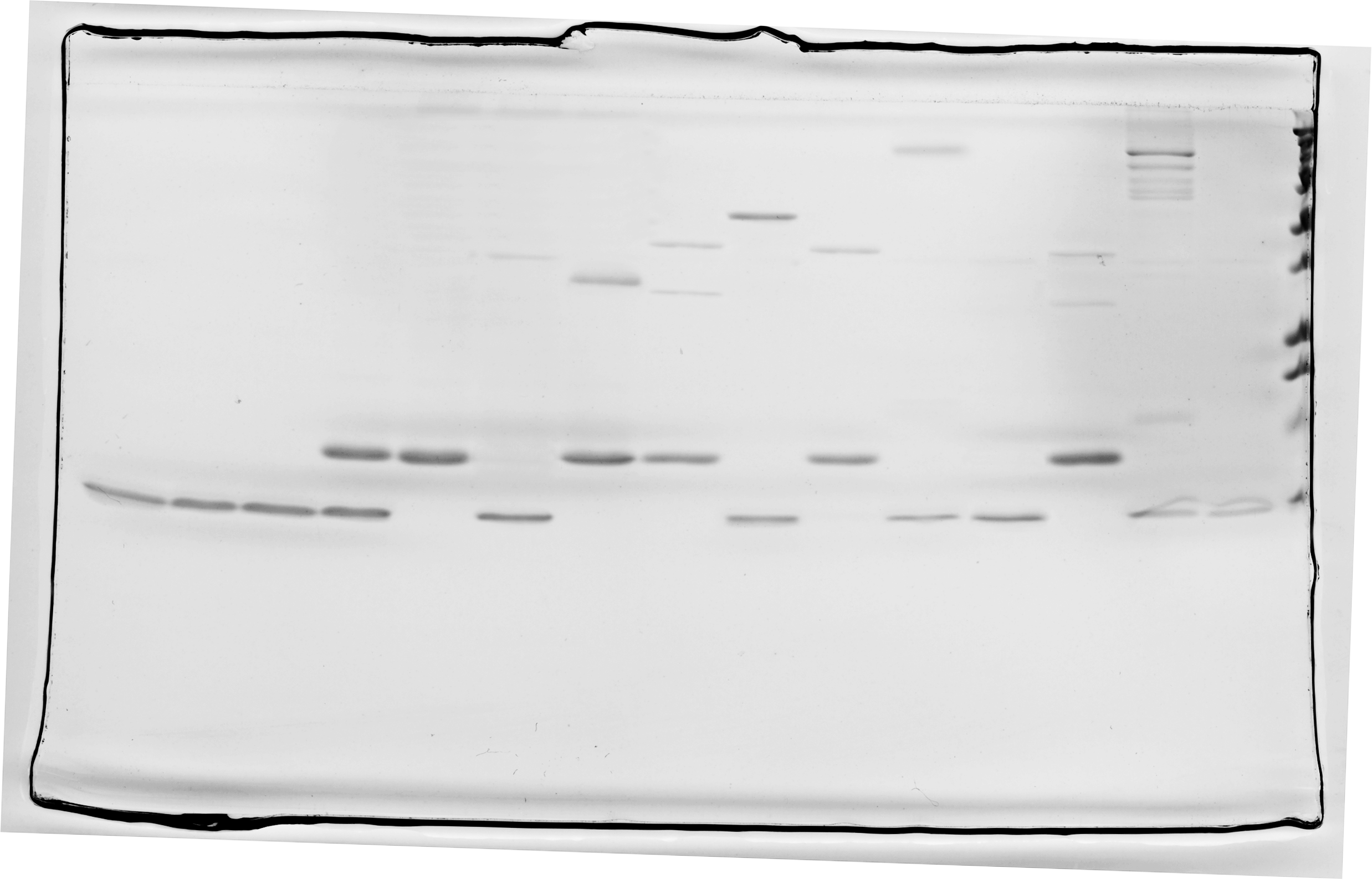

Supplement: Source data 1. [file elife-77424-data1.zip › Source data/Figure 1-figure supplemental 3/Figure 1-figure supplemental 3B.tif]

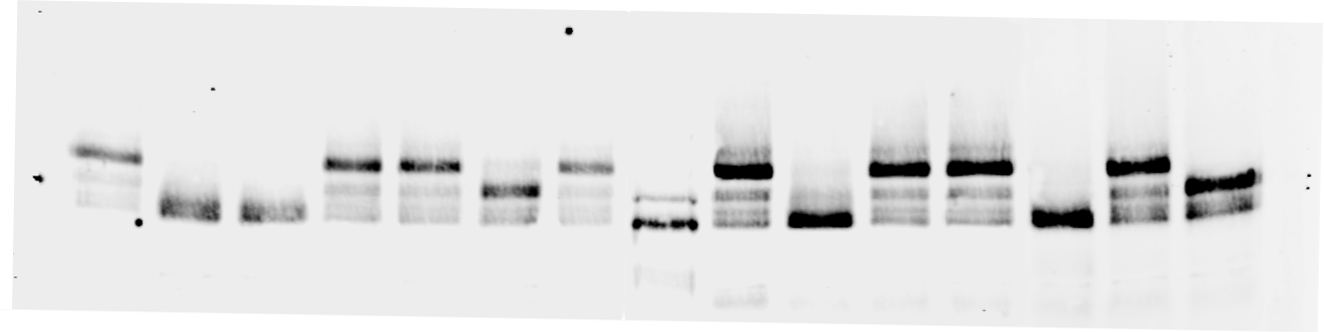

Supplement: Source data 1. [file elife-77424-data1.zip › Source data/Figure 1-figure supplemental 3/Figure 1-figure supplemental 3A_FLAG.tif]

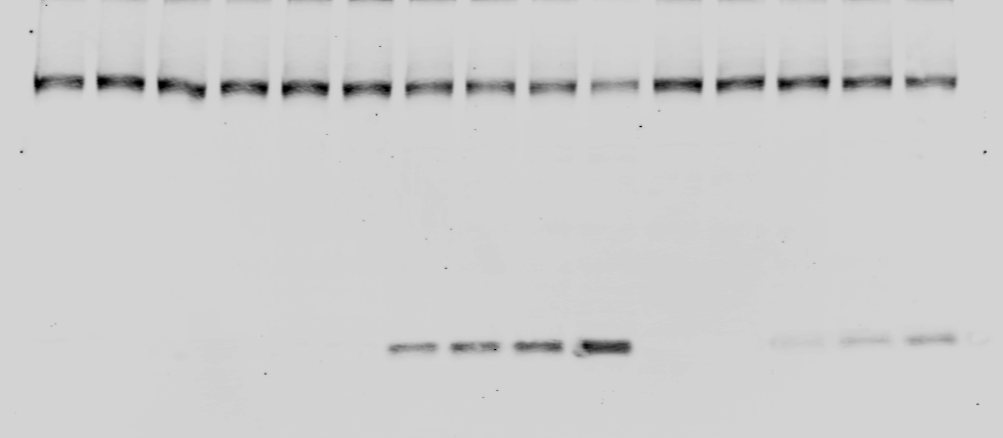

Supplement: Source data 1. [file elife-77424-data1.zip › Source data/Figure 2-figure supplemental 1/figure 2-figure supplemental 1A-GFP.tif]

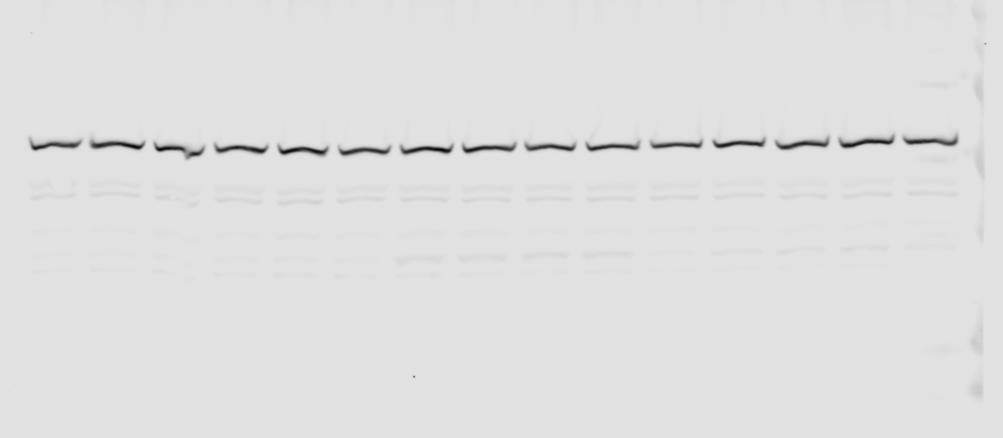

Supplement: Source data 1. [file elife-77424-data1.zip › Source data/Figure 2-figure supplemental 1/figure 2-figure supplemental 1A-G6PDH.tif]

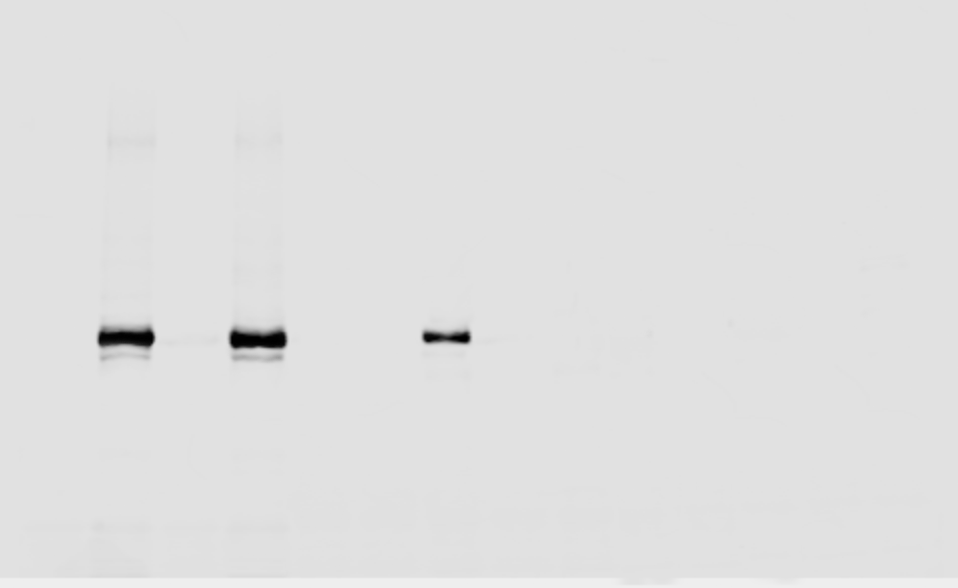

Supplement: Source data 1. [file elife-77424-data1.zip › Source data/Figure 2-figure supplemental 1/figure 2-figure supplemental 1E-FLAG.tif]

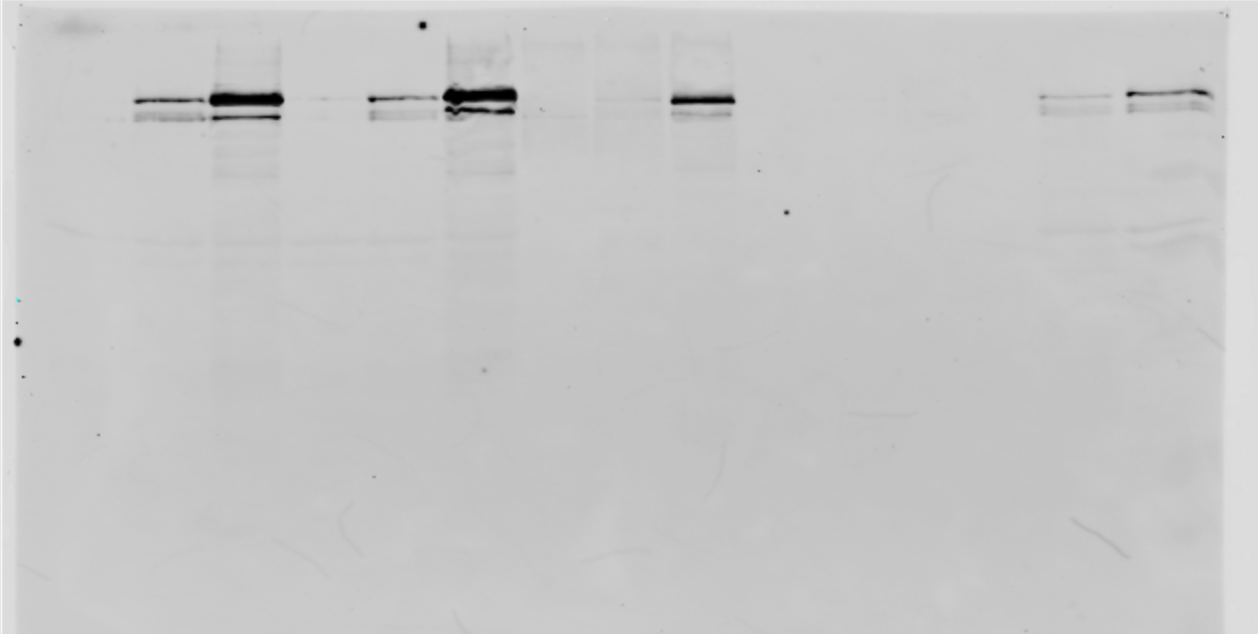

Supplement: Source data 1. [file elife-77424-data1.zip › Source data/Figure 2-figure supplemental 1/figure 2-figure supplemental 1D-FLAG.tif]

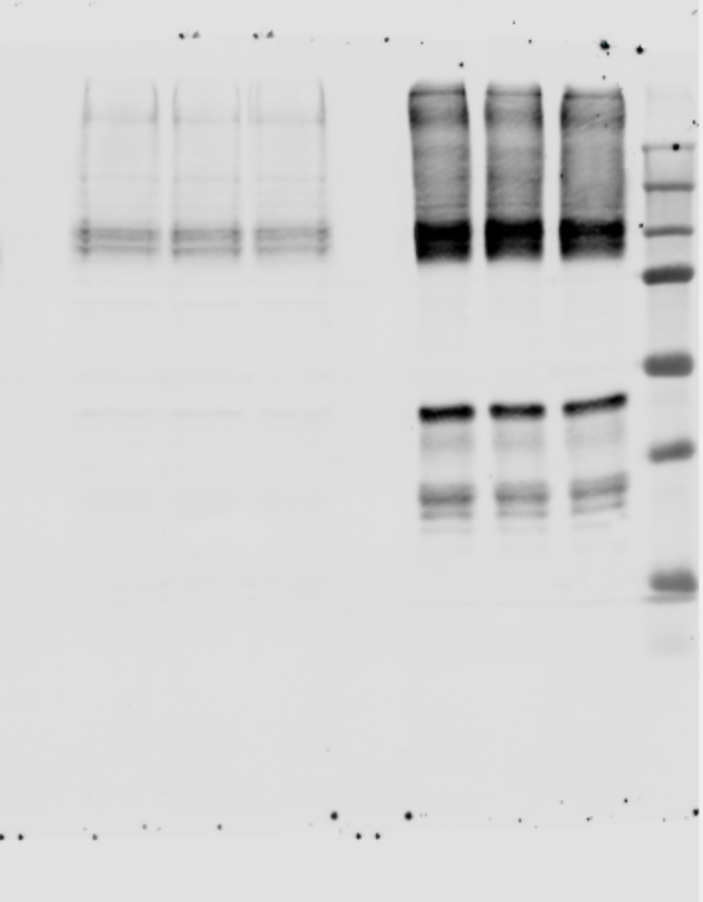

Supplement: Source data 1. [file elife-77424-data1.zip › Source data/Figure 2-figure supplemental 1/figure 2-figure supplemental 1F-GFP.tif]

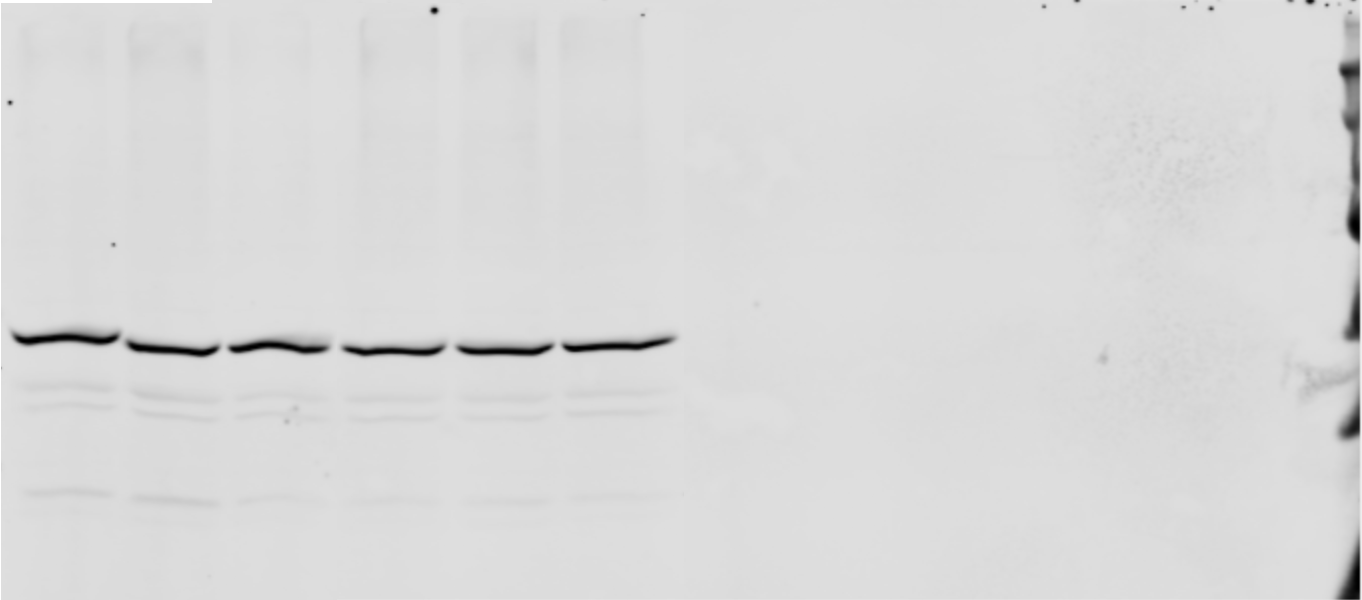

Supplement: Source data 1. [file elife-77424-data1.zip › Source data/Figure 2-figure supplemental 1/figure 2-figure supplemental 1D-G6PDH.tif]

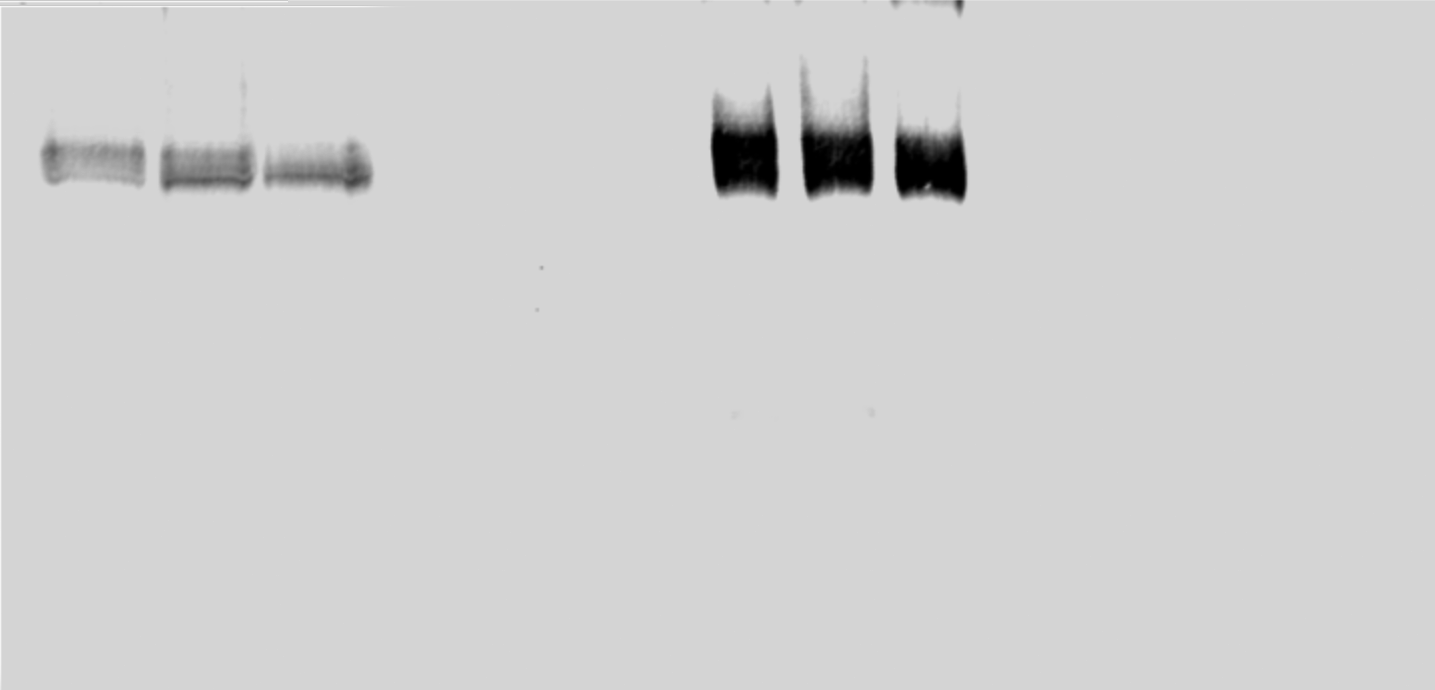

Supplement: Source data 1. [file elife-77424-data1.zip › Source data/Figure 2-figure supplemental 1/figure 2-figure supplemental 1D-GFP.tif]

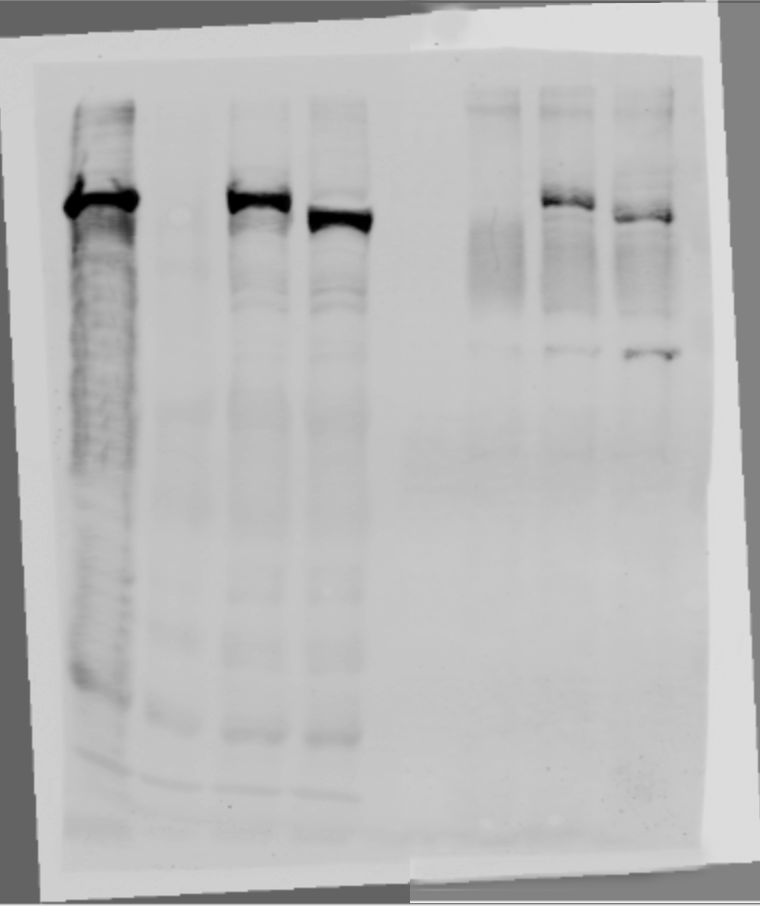

Supplement: Source data 1. [file elife-77424-data1.zip › Source data/Figure 2-figure supplemental 1/figure 2-figure supplemental 1F-FLAG.tif]

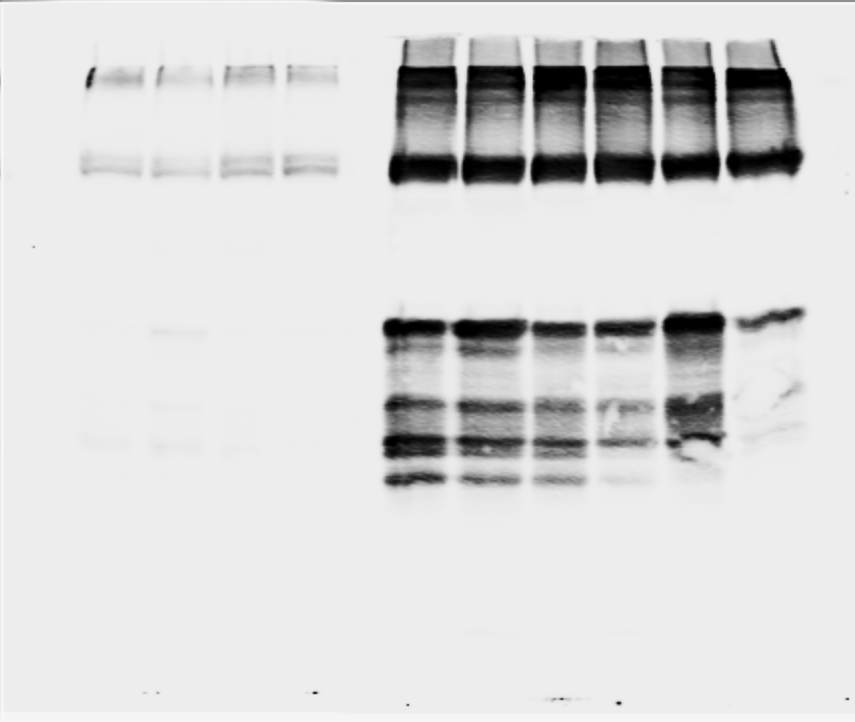

Supplement: Source data 1. [file elife-77424-data1.zip › Source data/Figure 2-figure supplemental 1/figure 2-figure supplemental 1E-GFP.tif]

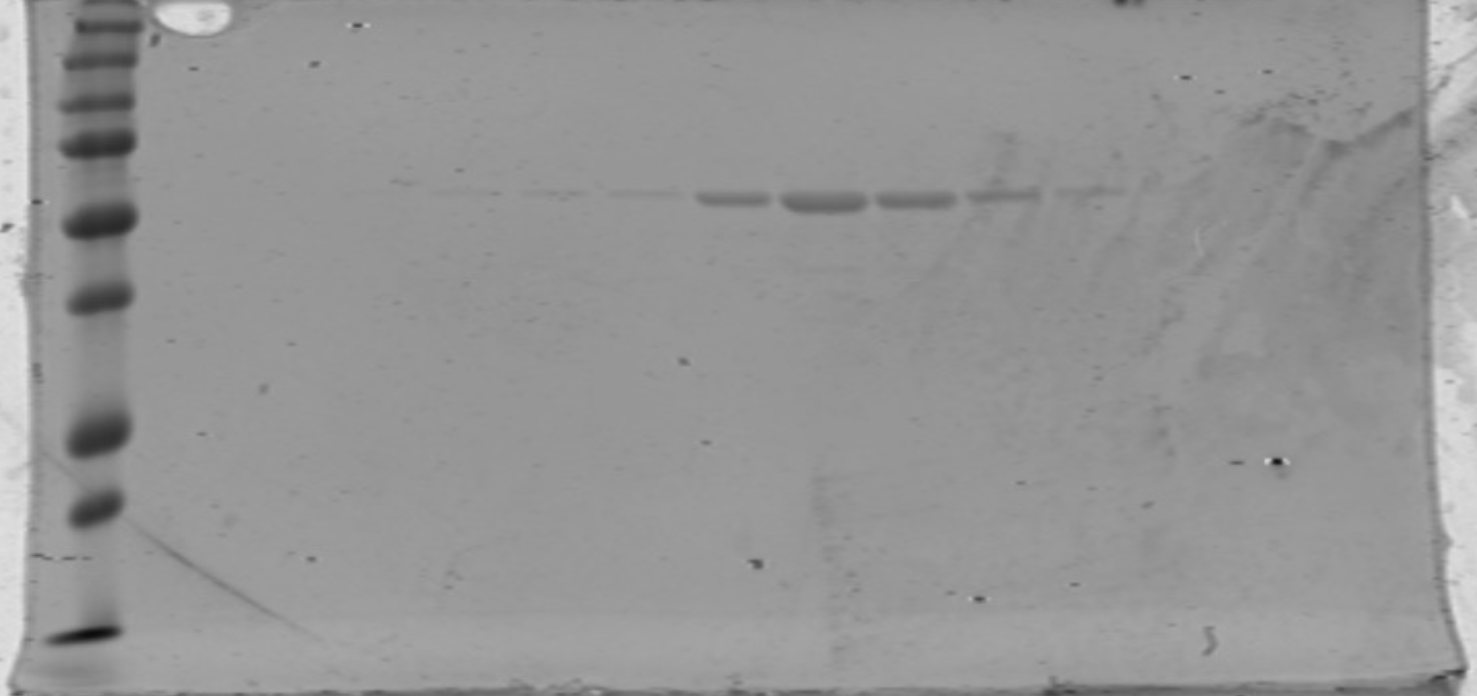

Supplement: Source data 1. [file elife-77424-data1.zip › Source data/Figure 5-figure supplemental 2/Figure 5-figure supplemental 1B-1.tif]

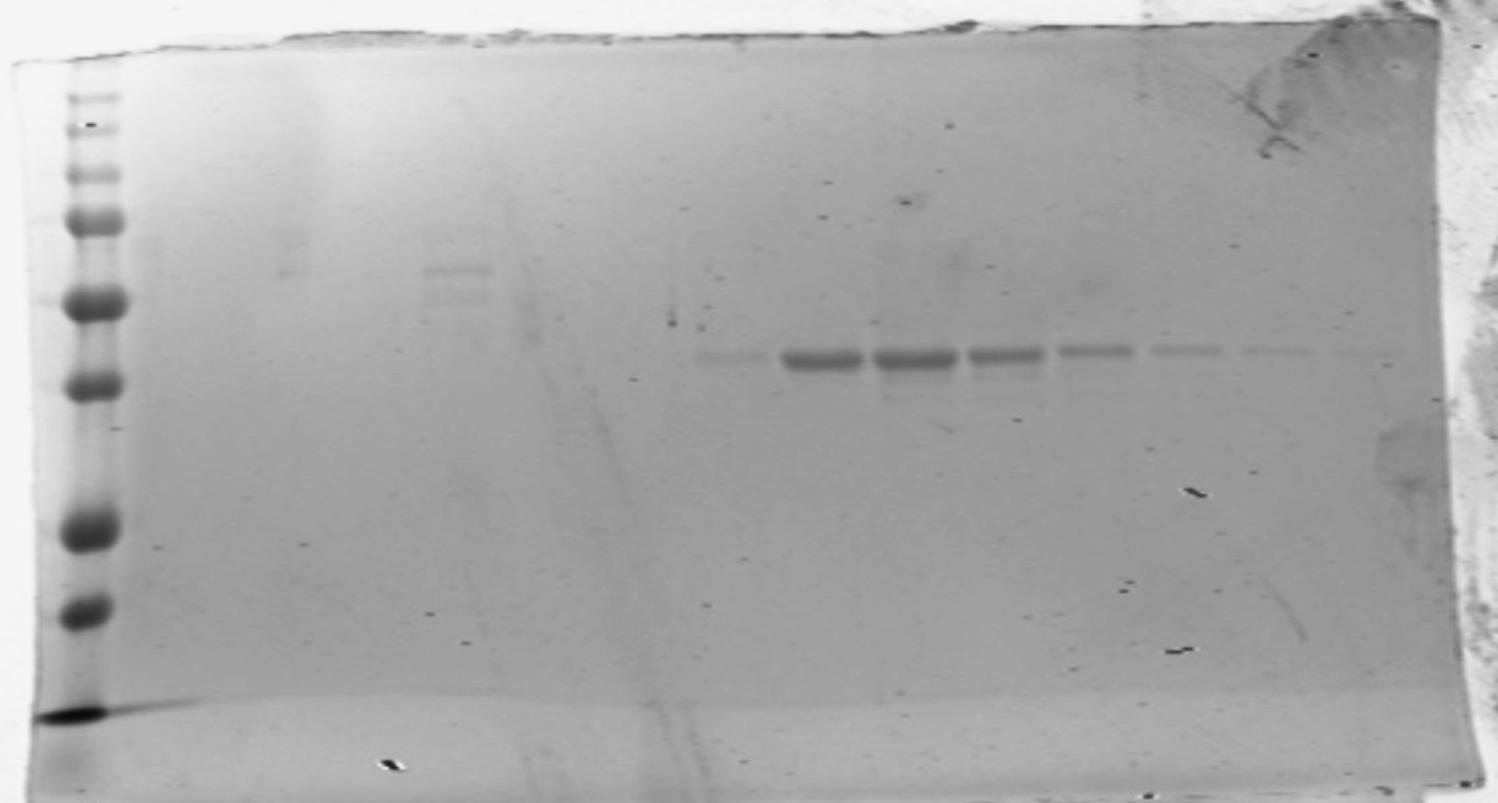

Supplement: Source data 1. [file elife-77424-data1.zip › Source data/Figure 5-figure supplemental 2/Figure 5-figure supplemental 1A.tif]

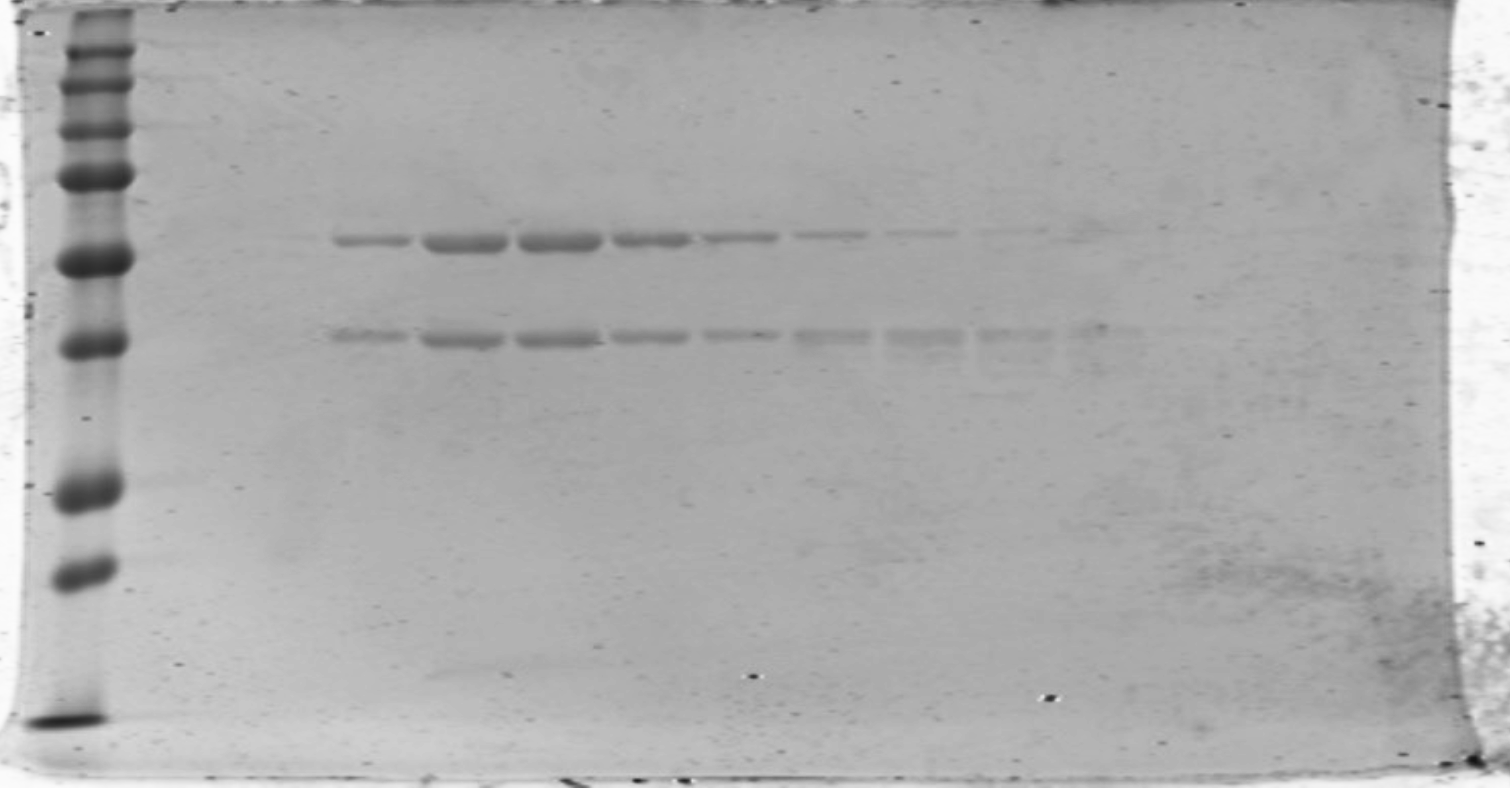

Supplement: Source data 1. [file elife-77424-data1.zip › Source data/Figure 5-figure supplemental 2/Figure 5-figure supplemental 1C-1.tif]

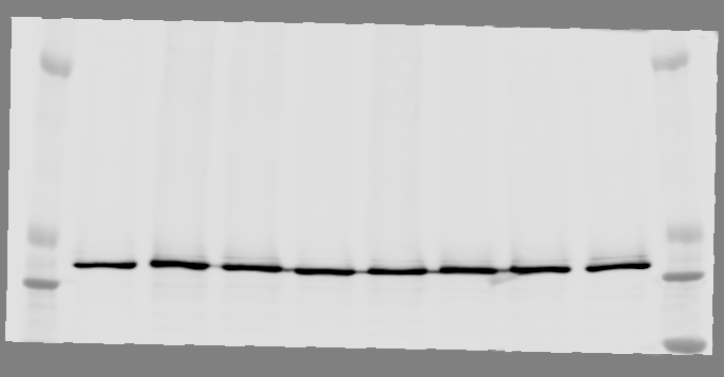

Supplement: Source data 1. [file elife-77424-data1.zip › Source data/Figure 6-figure supplemental 1/figure 6-figure supplemental 1A-G6PDH.tif]

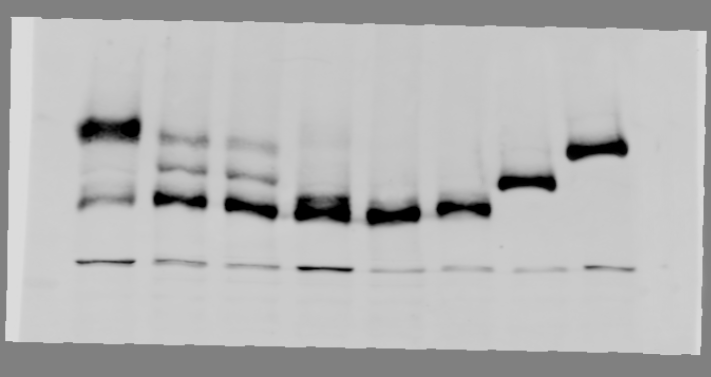

Supplement: Source data 1. [file elife-77424-data1.zip › Source data/Figure 6-figure supplemental 1/figure 6-figure supplemental 1A-FLAG.tif]

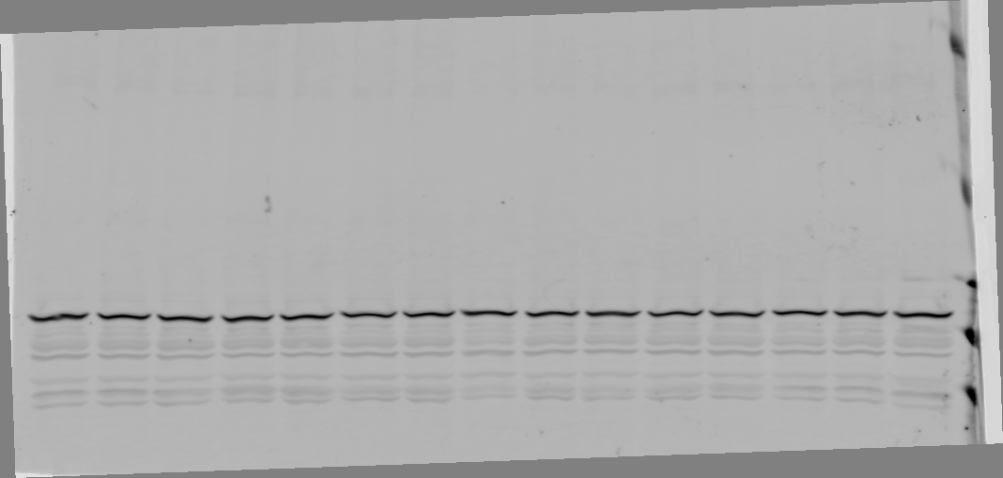

Supplement: Source data 1. [file elife-77424-data1.zip › Source data/Figure 6-figure supplemental 1/figure 6-figure supplemental 1B-G6PDH.tif]

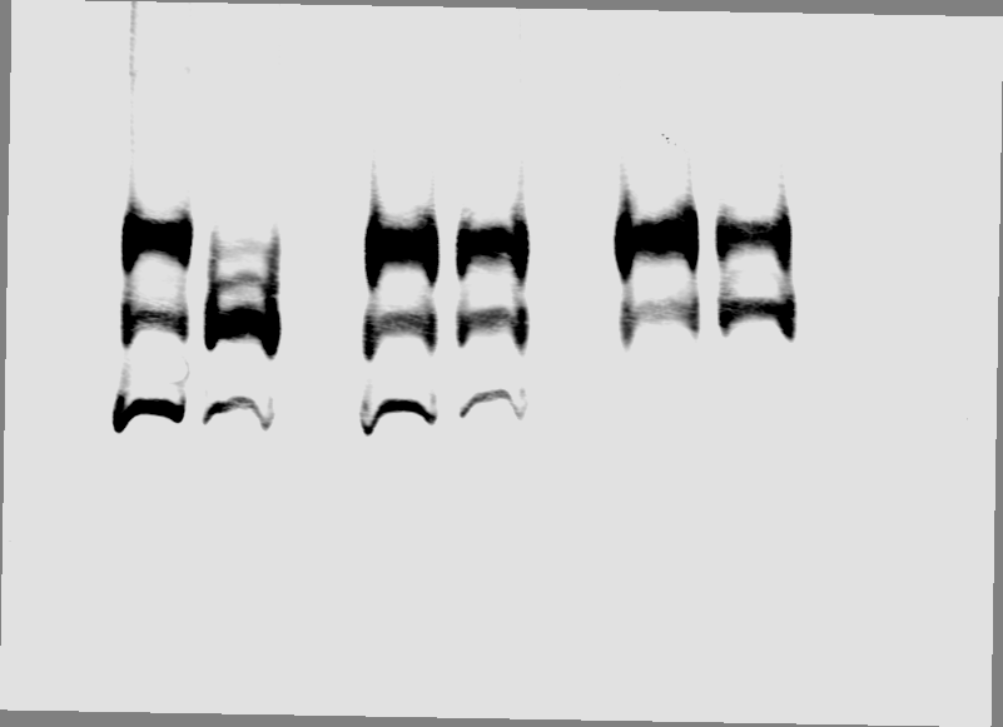

Supplement: Source data 1. [file elife-77424-data1.zip › Source data/Figure 6-figure supplemental 1/figure 6-figure supplemental 1D-FLAG.tif]

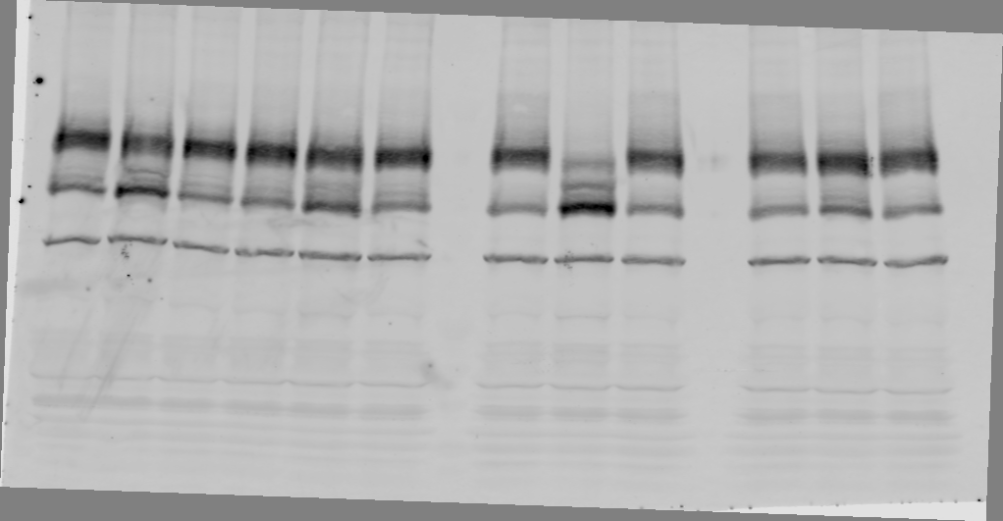

Supplement: Source data 1. [file elife-77424-data1.zip › Source data/Figure 6-figure supplemental 1/figure 6-figure supplemental 1E-FLAG.tif]

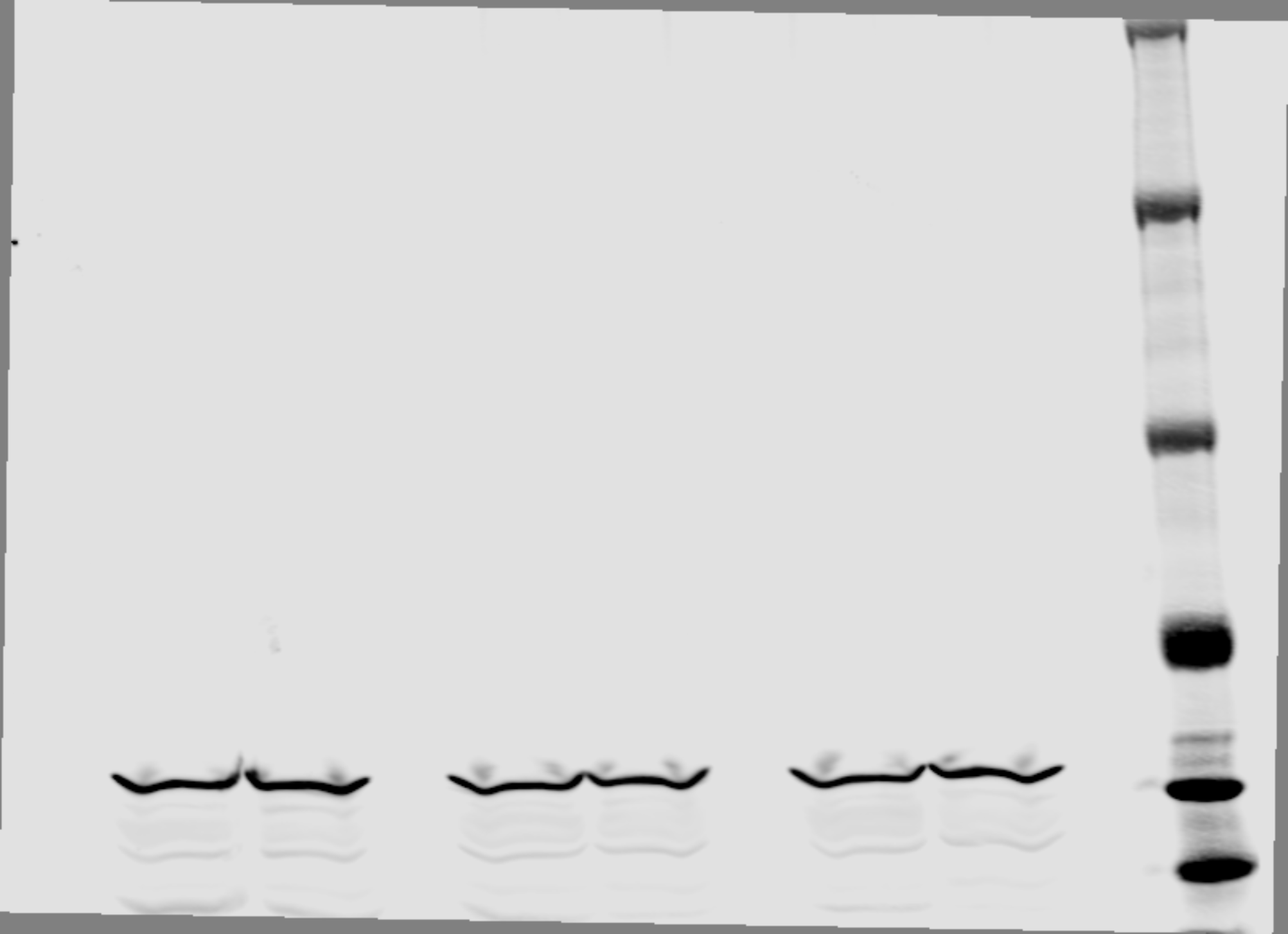

Supplement: Source data 1. [file elife-77424-data1.zip › Source data/Figure 6-figure supplemental 1/figure 6-figure supplemental 1D-G6PDH.tif]

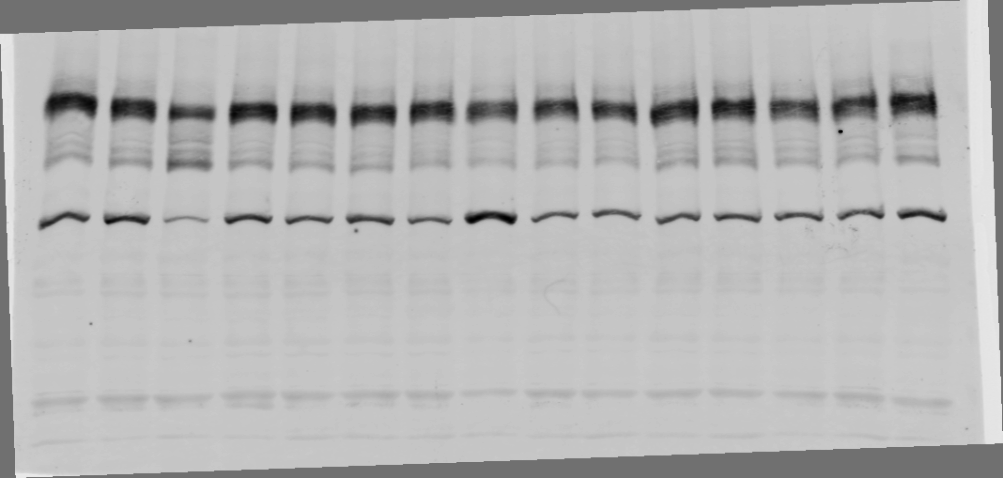

Supplement: Source data 1. [file elife-77424-data1.zip › Source data/Figure 6-figure supplemental 1/figure 6-figure supplemental 1B-FLAG.tif]

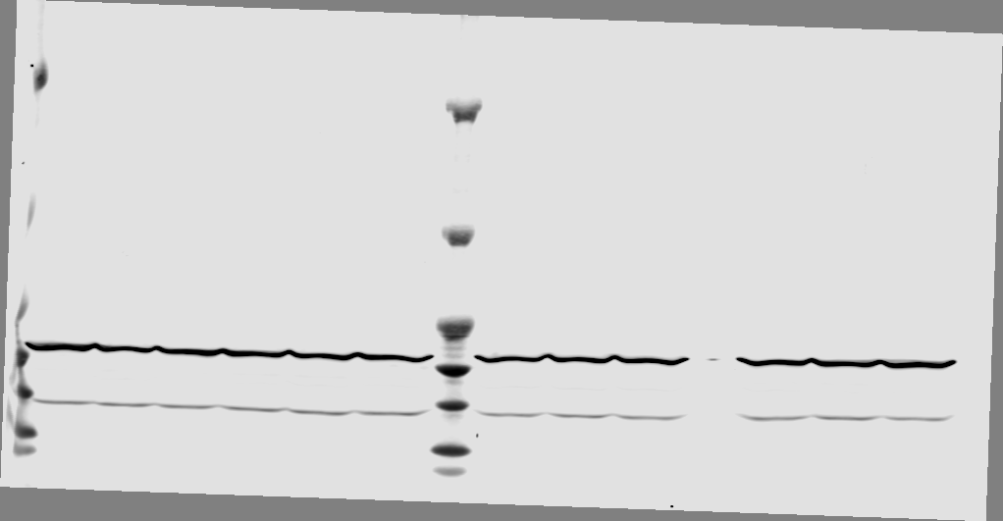

Supplement: Source data 1. [file elife-77424-data1.zip › Source data/Figure 6-figure supplemental 1/figure 6-figure supplemental 1E-G6PDH.tif]

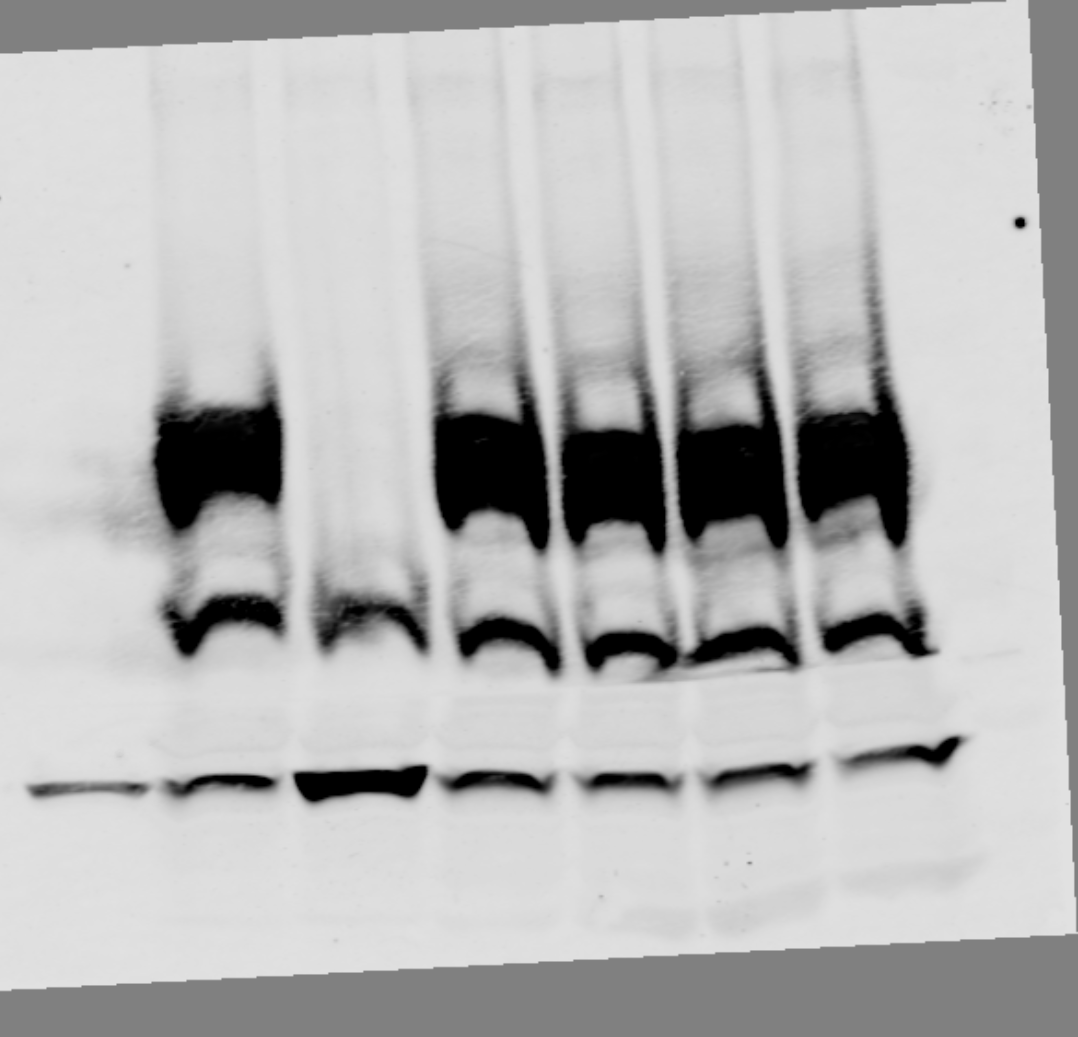

Supplement: Source data 1. [file elife-77424-data1.zip › Source data/Figure 6/figure 6A-HA.tif]

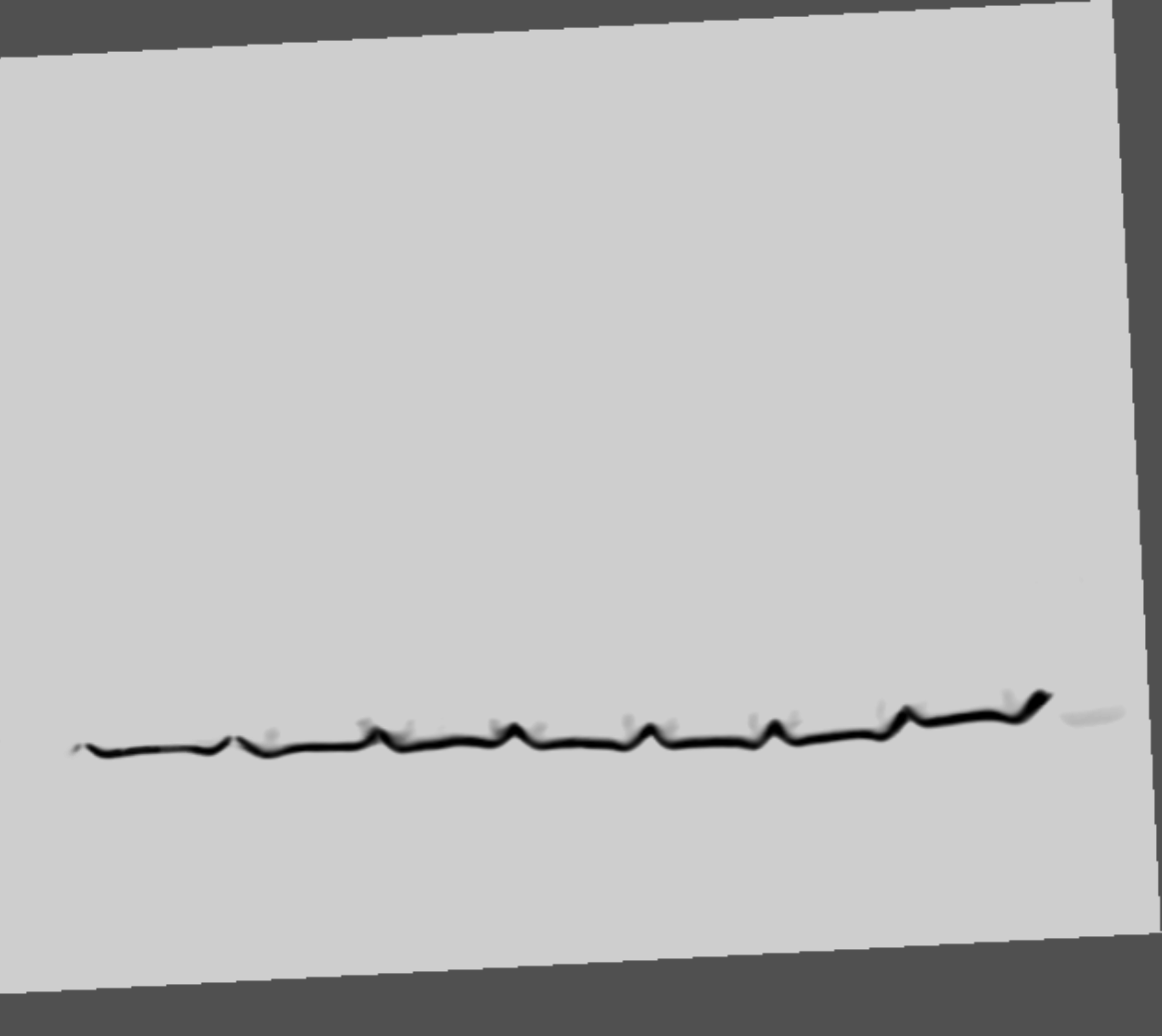

Supplement: Source data 1. [file elife-77424-data1.zip › Source data/Figure 6/figure 6A-G6PDH.tif]

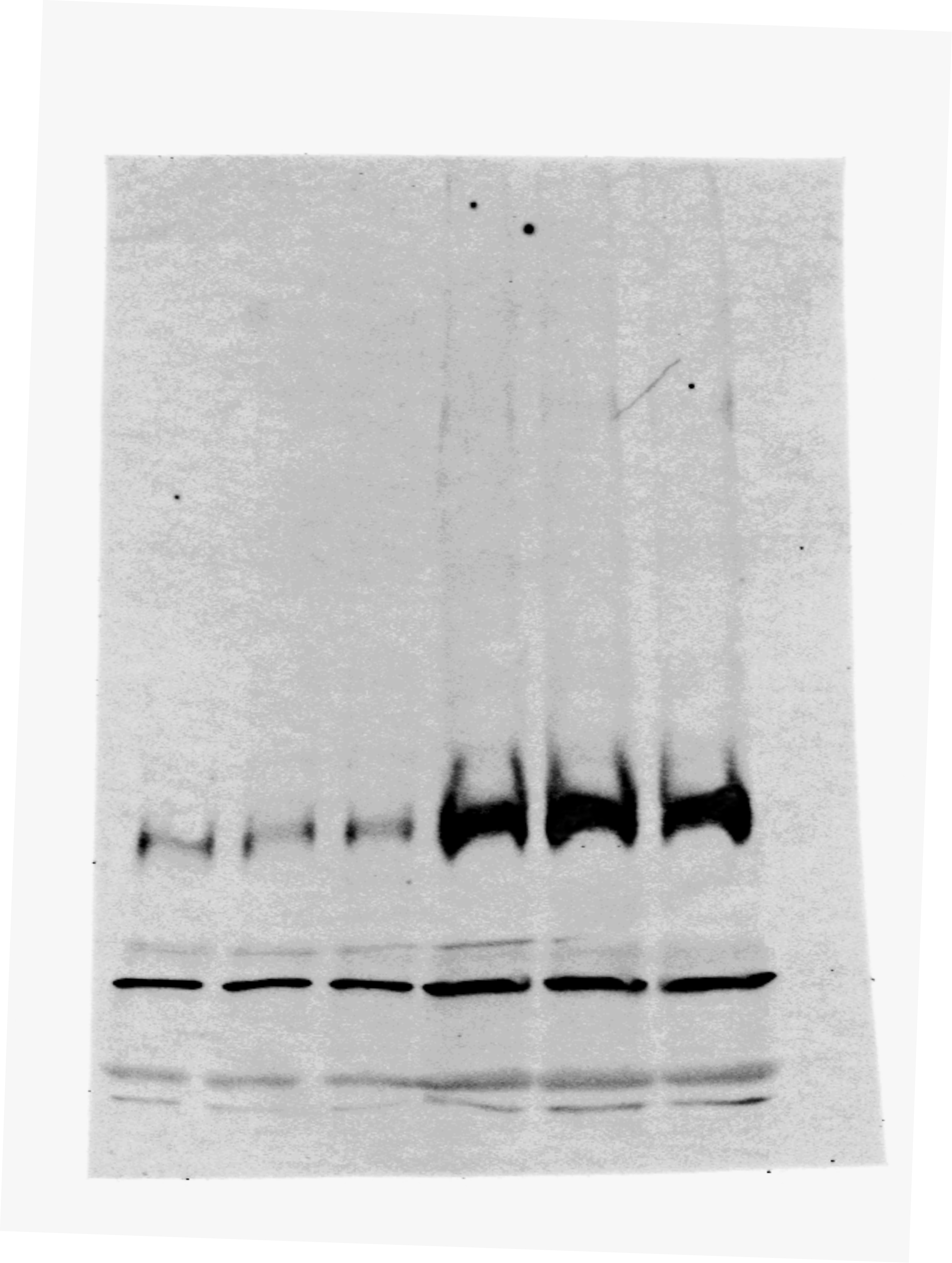

Supplement: Source data 1. [file elife-77424-data1.zip › Source data/Figure 6/Figure 6C-HA.tif]

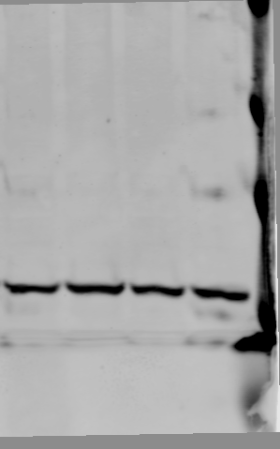

Supplement: Source data 1. [file elife-77424-data1.zip › Source data/Figure 6/figure 6D-G6PDH.tif]

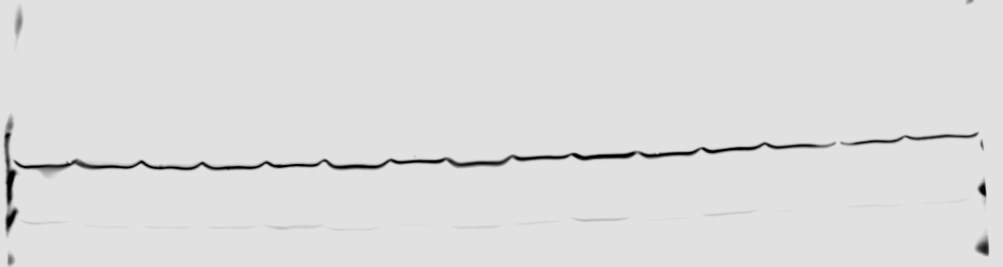

Supplement: Source data 1. [file elife-77424-data1.zip › Source data/Figure 6/figure 6B-G6PDH.tif]

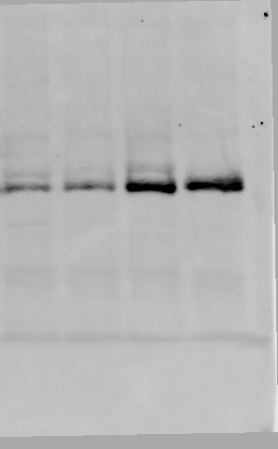

Supplement: Source data 1. [file elife-77424-data1.zip › Source data/Figure 6/figure 6D-HA.tif]

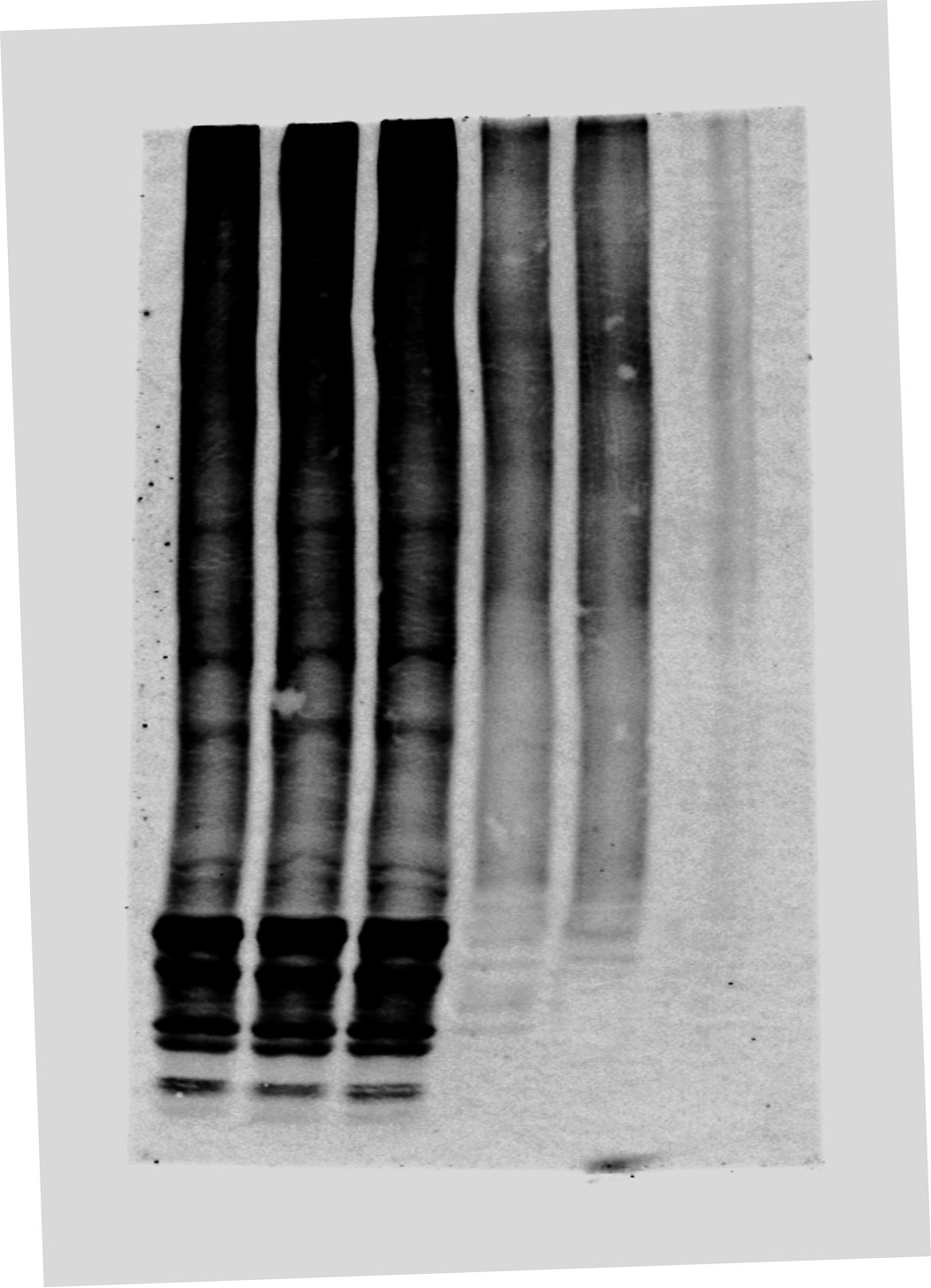

Supplement: Source data 1. [file elife-77424-data1.zip › Source data/Figure 6/Figure 6C-myc.tif]

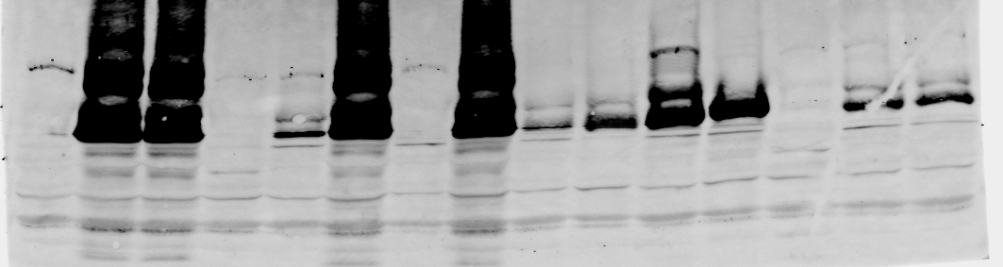

Supplement: Source data 1. [file elife-77424-data1.zip › Source data/Figure 6/figure 6B-HA.tif]

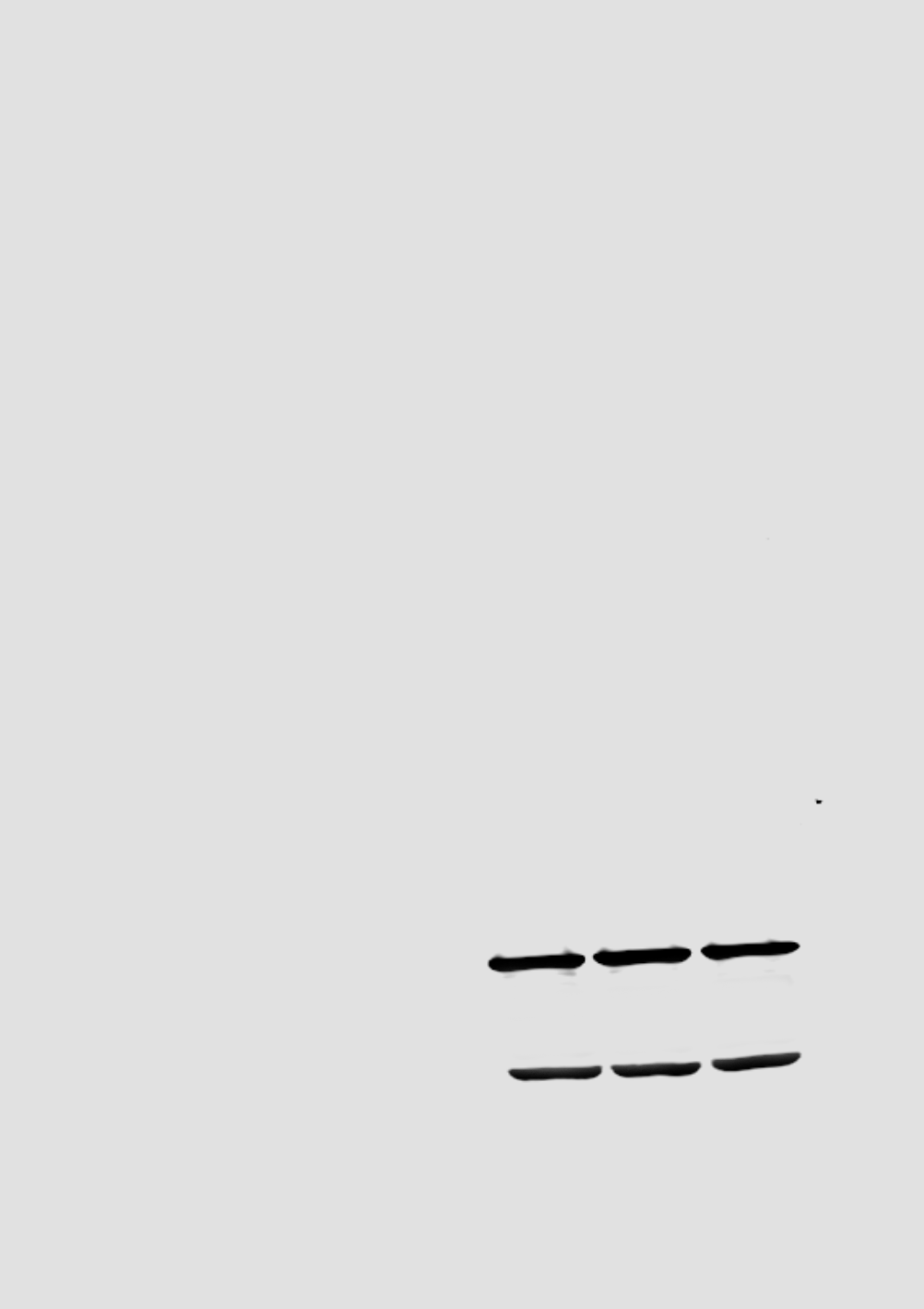

Supplement: Source data 1. [file elife-77424-data1.zip › Source data/Figure 6/Figure 6C-G6PDH.tif]

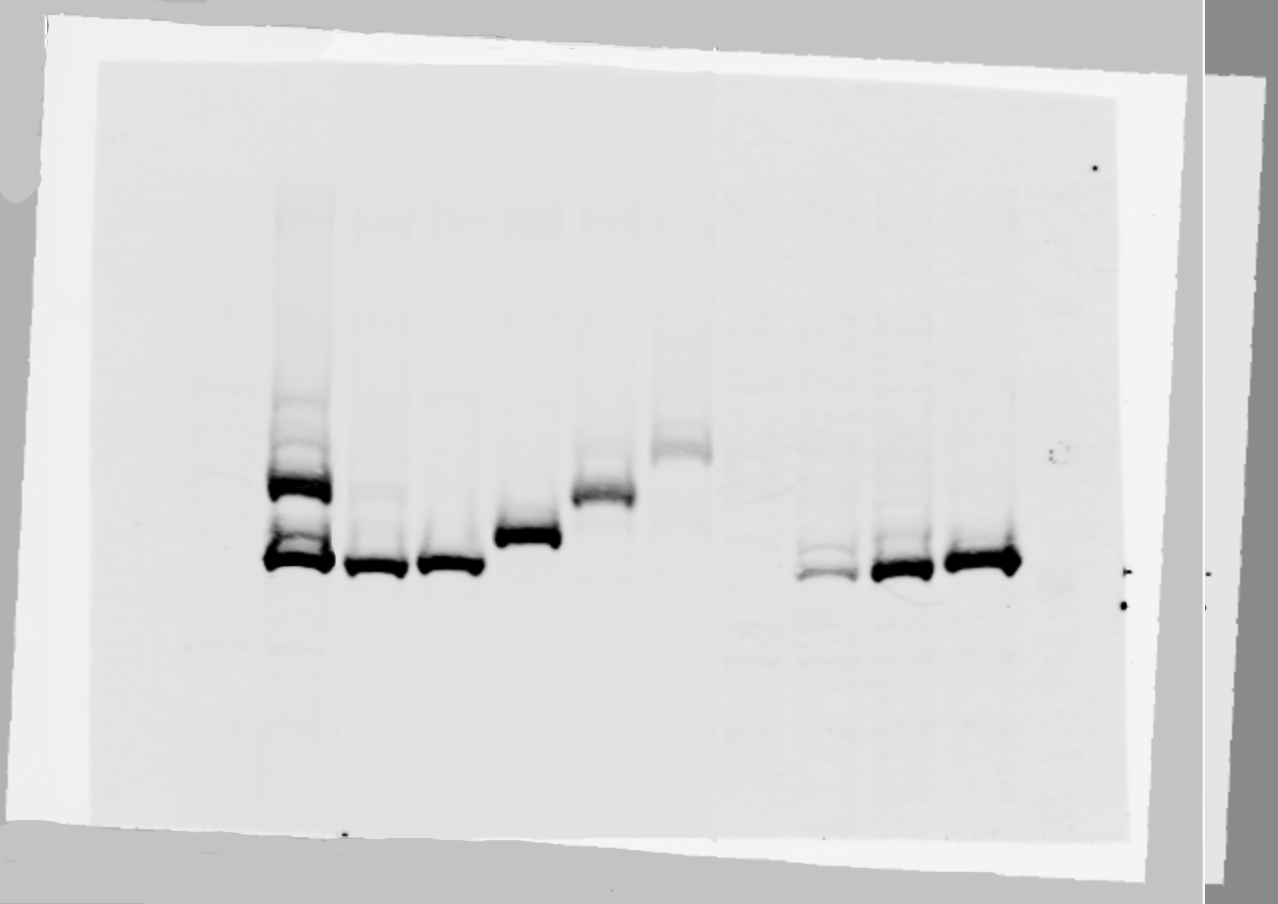

Supplement: Source data 1. [file elife-77424-data1.zip › Source data/Figure 1/Figure 1C_Art5_Ub_WT_K63R_FLAG.tif]

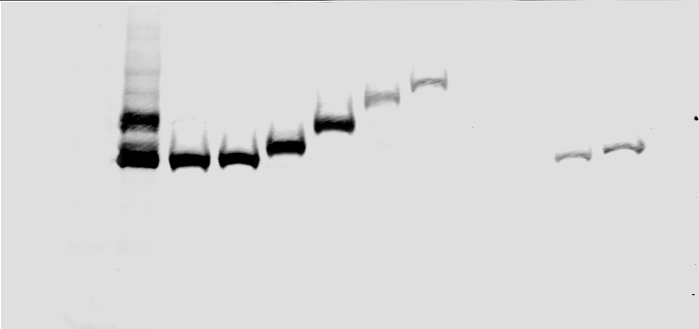

Supplement: Source data 1. [file elife-77424-data1.zip › Source data/Figure 1/Figure 1B_Art5-HTF.tif]

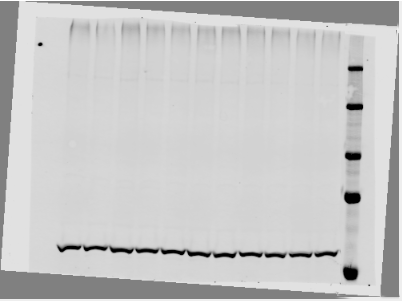

Supplement: Source data 1. [file elife-77424-data1.zip › Source data/Figure 1/Figure 1C_Art5_Ub_WT_K63R_G6PDHpng.png]

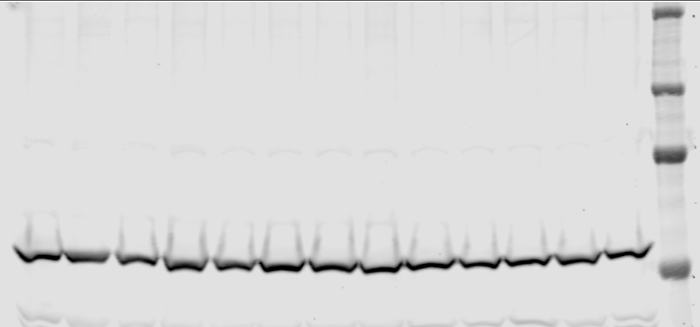

Supplement: Source data 1. [file elife-77424-data1.zip › Source data/Figure 1/Figure 1B_G6PDH.tif]

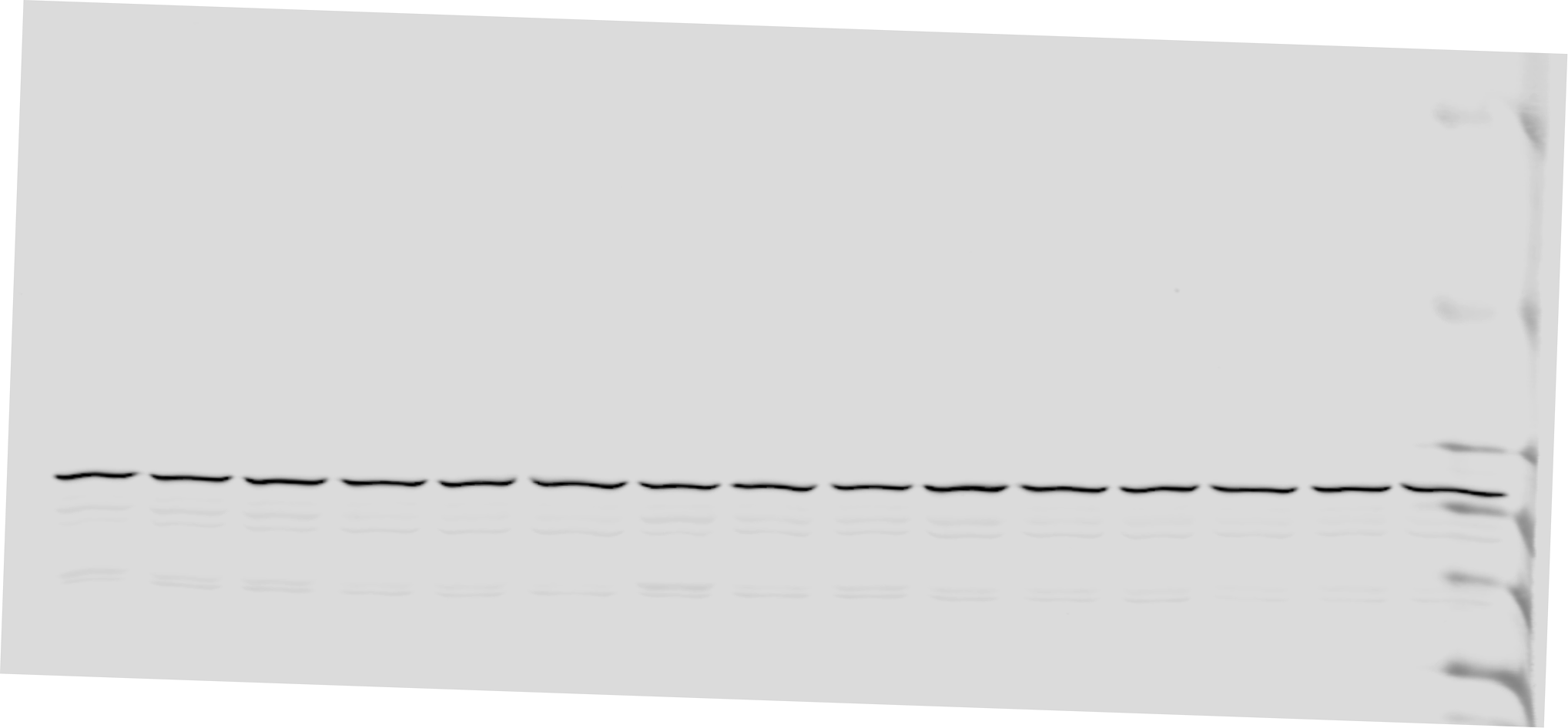

Supplement: Source data 1. [file elife-77424-data1.zip › Source data/Figure 1-figure supplemental 1/Figure 1-figure supplemental 1C_G6PDH.tif]

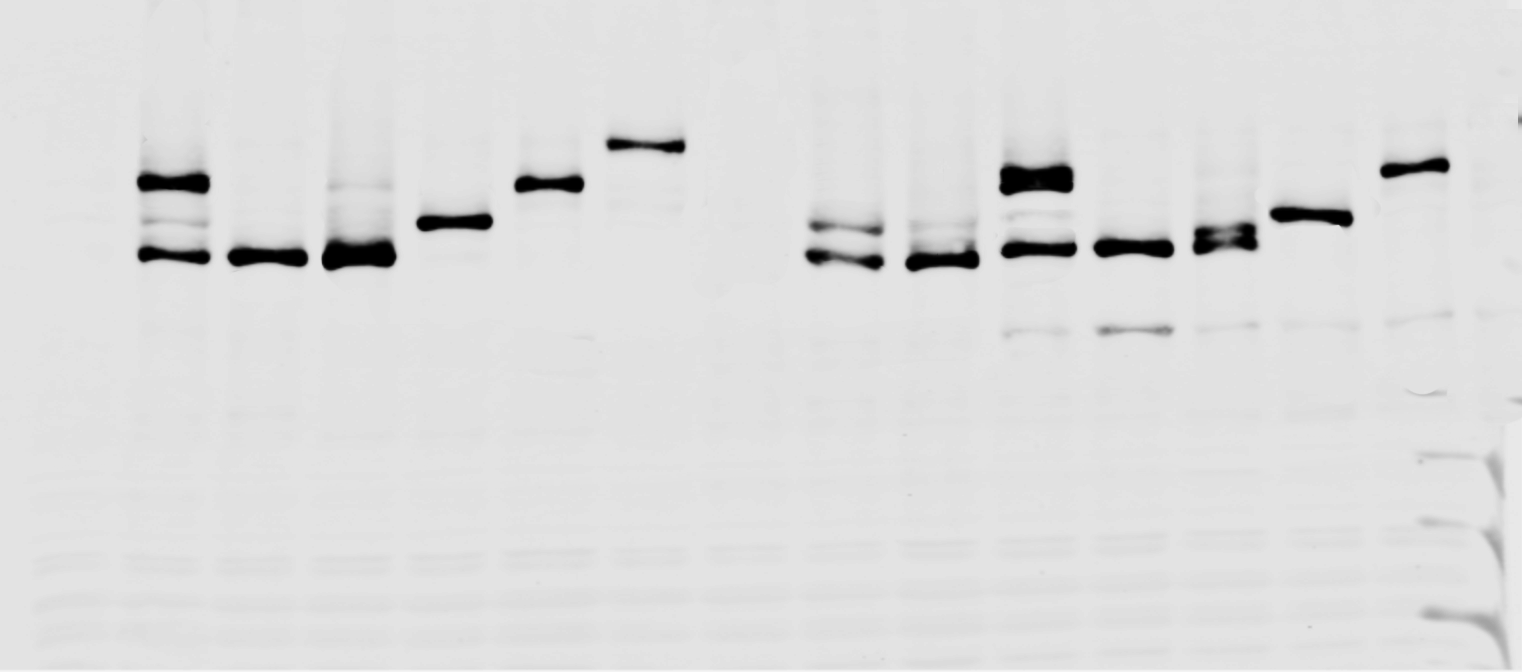

Supplement: Source data 1. [file elife-77424-data1.zip › Source data/Figure 1-figure supplemental 1/Figure 1-figure supplemental 1C_art1-diUb-k63linked-ub_new.tif]

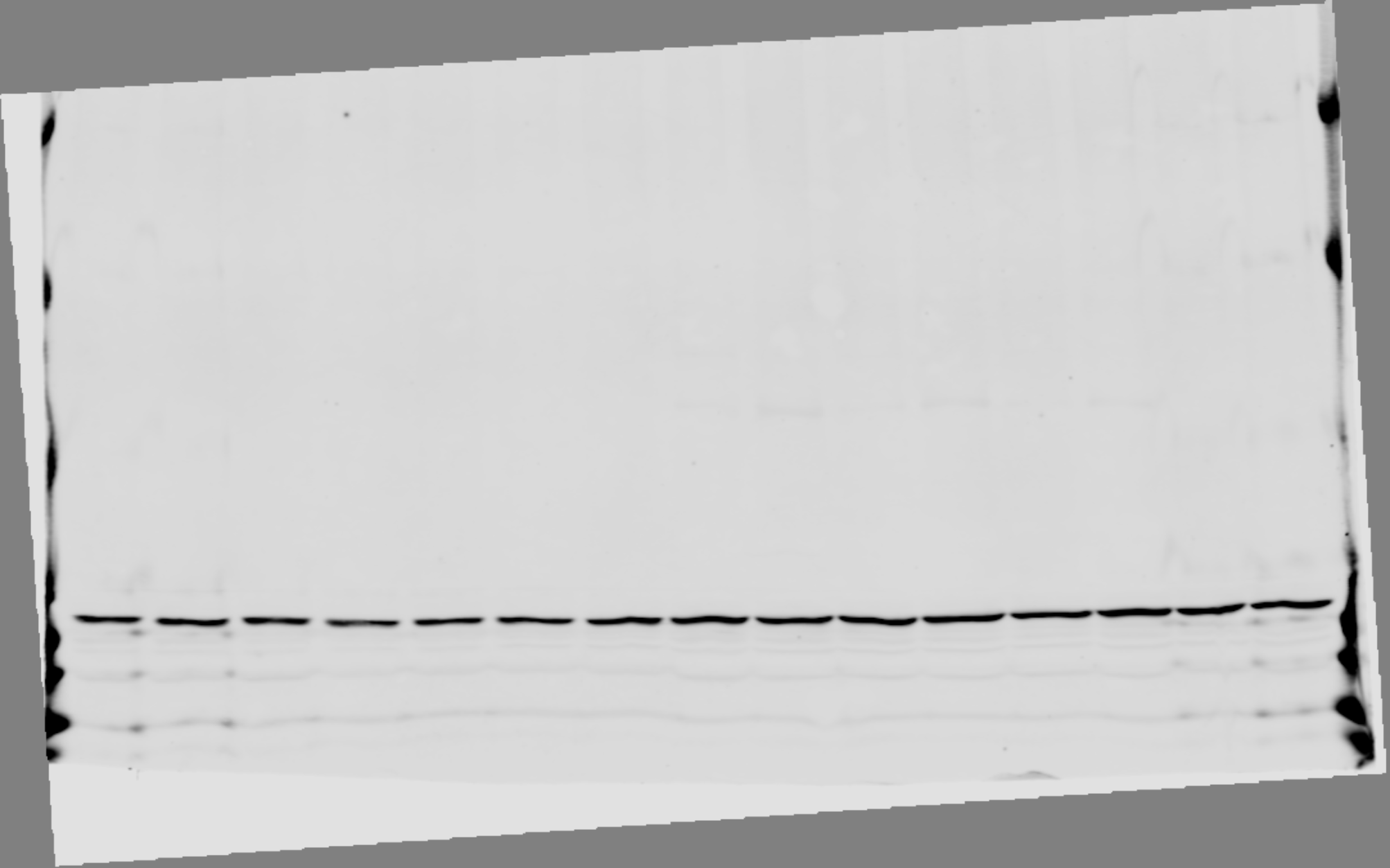

Supplement: Source data 1. [file elife-77424-data1.zip › Source data/Figure 1-figure supplemental 1/Figure 1-figure supplemental 1B_G6PDH.tif]

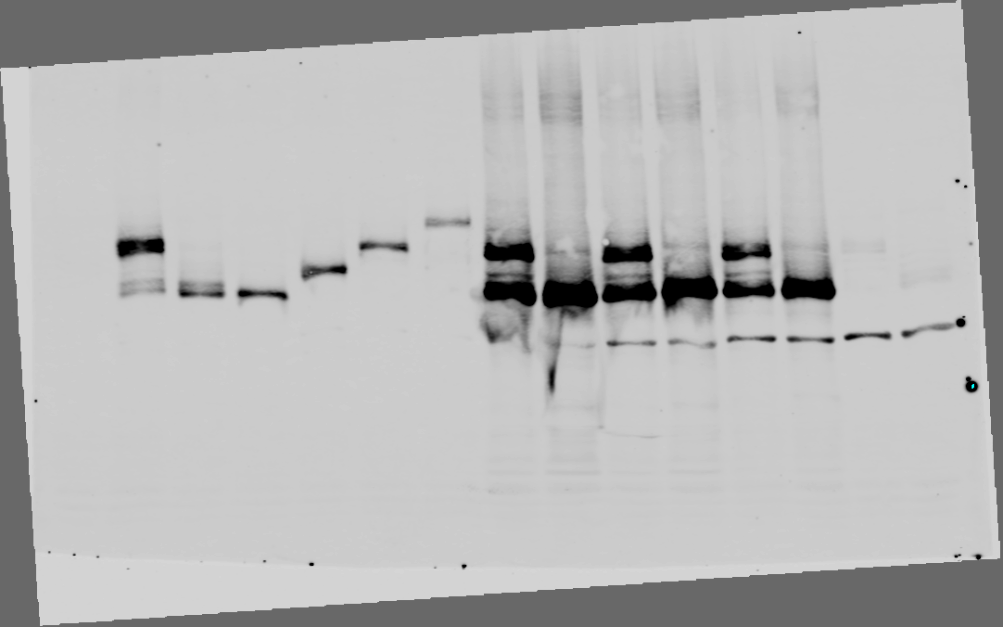

Supplement: Source data 1. [file elife-77424-data1.zip › Source data/Figure 1-figure supplemental 1/Figure 1-figure supplemental 1B_FLAG_.tif]

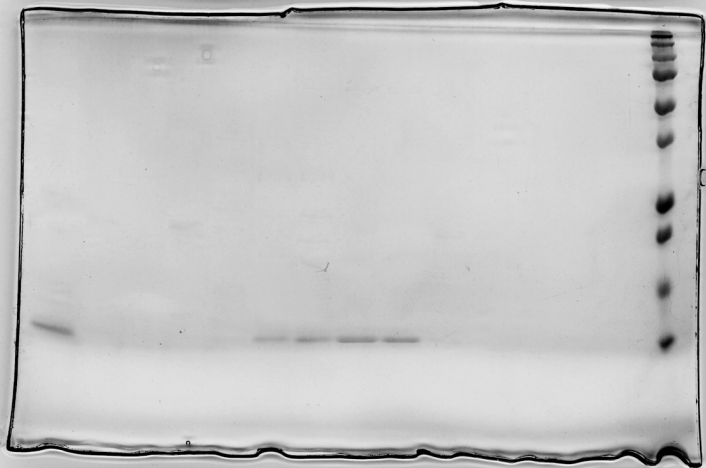

Supplement: Source data 1. [file elife-77424-data1.zip › Source data/Supplementary file 2/8-(A) Art1PY-purification.pdf]

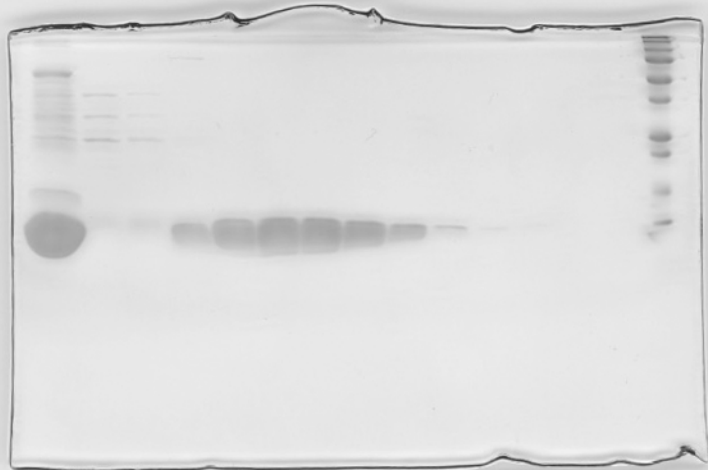

Supplement: Source data 1. [file elife-77424-data1.zip › Source data/Supplementary file 2/1-(A) Ub purification.pdf]

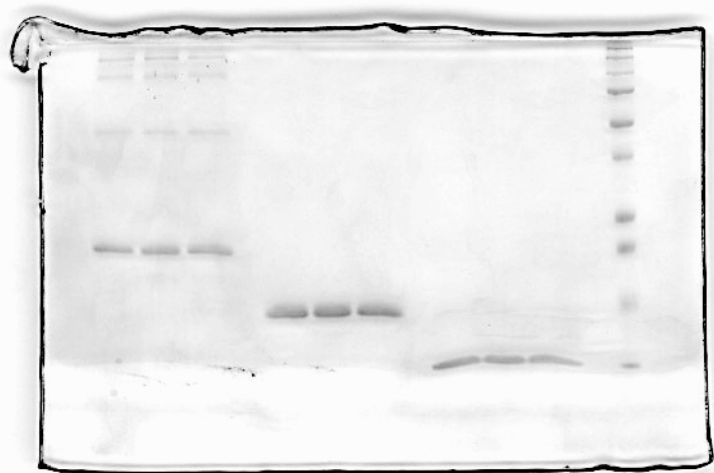

Supplement: Source data 1. [file elife-77424-data1.zip › Source data/Supplementary file 2/2-K63 linked di-Ub purification.pdf]

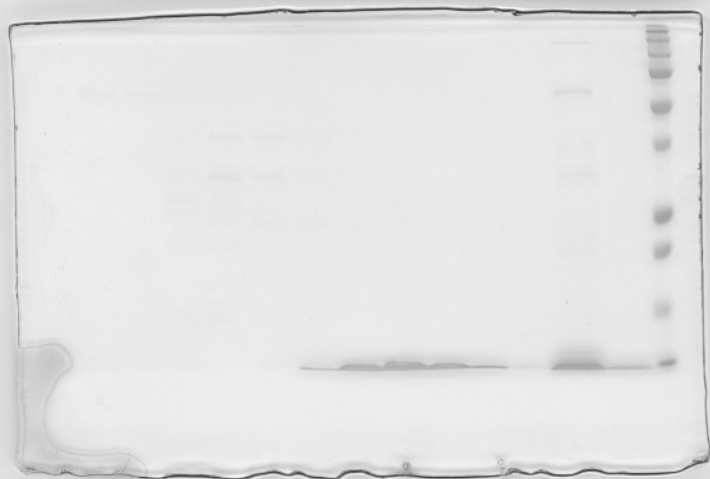

Supplement: Source data 1. [file elife-77424-data1.zip › Source data/Supplementary file 2/1-(B) Ub-I44A purification.pdf]

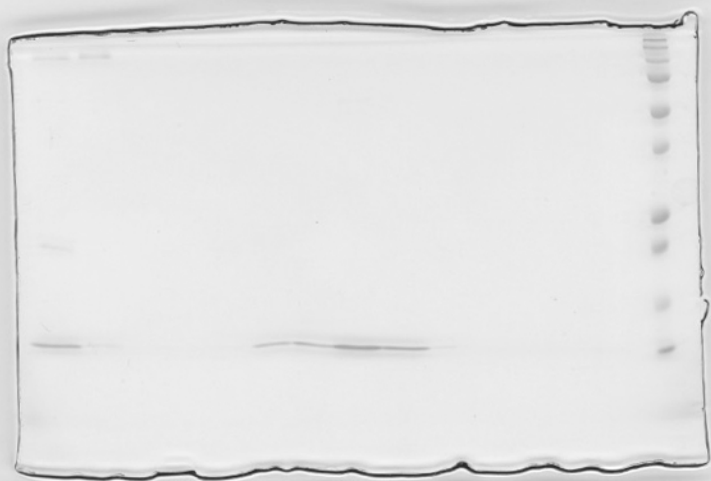

Supplement: Source data 1. [file elife-77424-data1.zip › Source data/Supplementary file 2/8-(B) Art1PY mutant-purification.pdf]

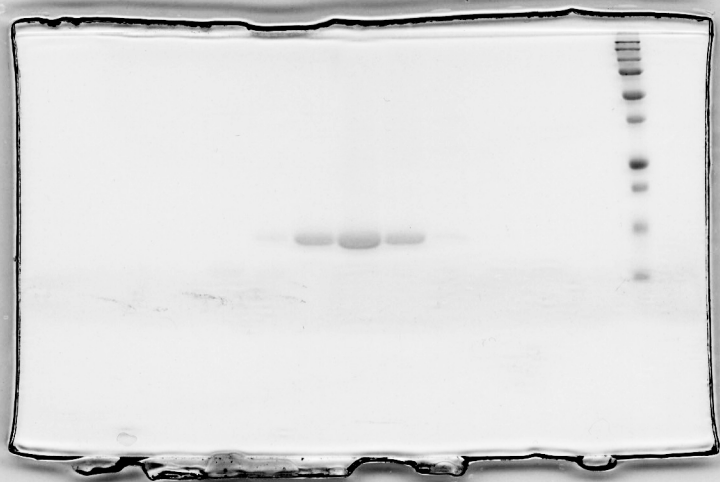

Supplement: Source data 1. [file elife-77424-data1.zip › Source data/Supplementary file 2/3-M1 linked di-Ub purification.pdf]

C12 D1

D12 E1

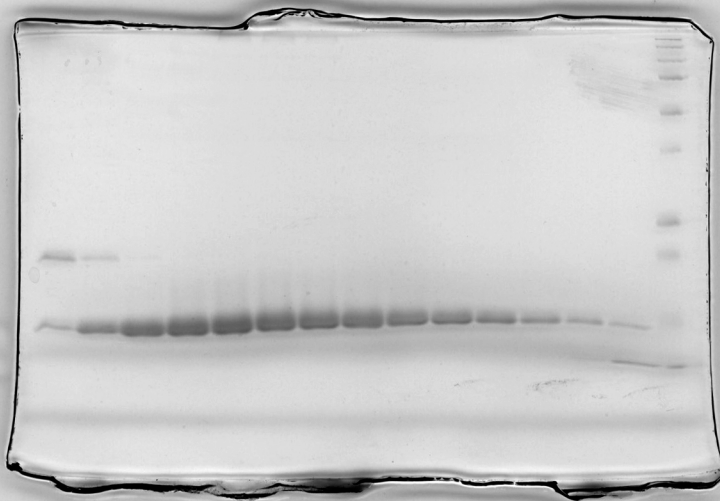

Supplement: Source data 1. [file elife-77424-data1.zip › Source data/Supplementary file 2/5-K63 linked di-Ub (Ubwt-UbI44A) purification.pdf]

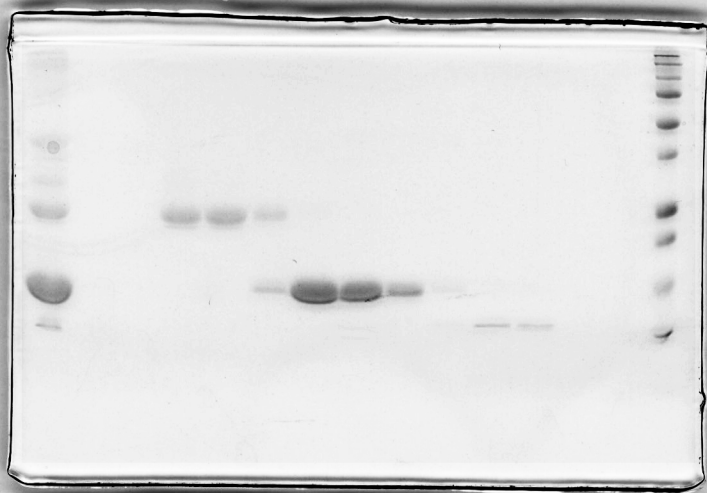

Supplement: Source data 1. [file elife-77424-data1.zip › Source data/Supplementary file 2/4-K48 linked di-Ub purification.pdf]

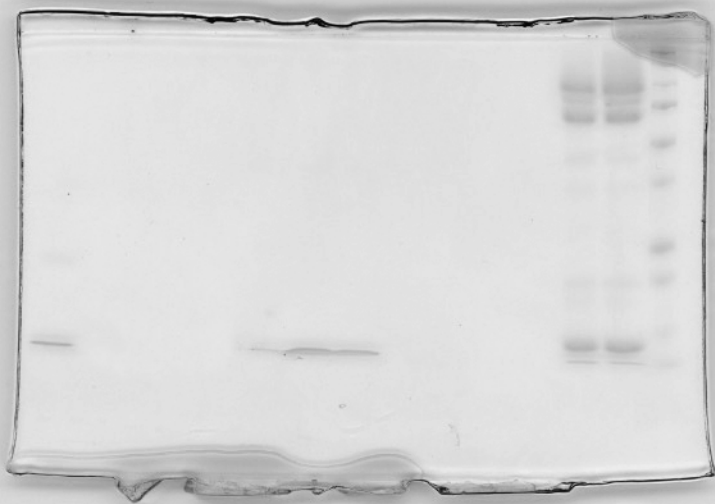

Supplement: Source data 1. [file elife-77424-data1.zip › Source data/Supplementary file 2/9-(B) Art5PY mutant-purification.pdf]

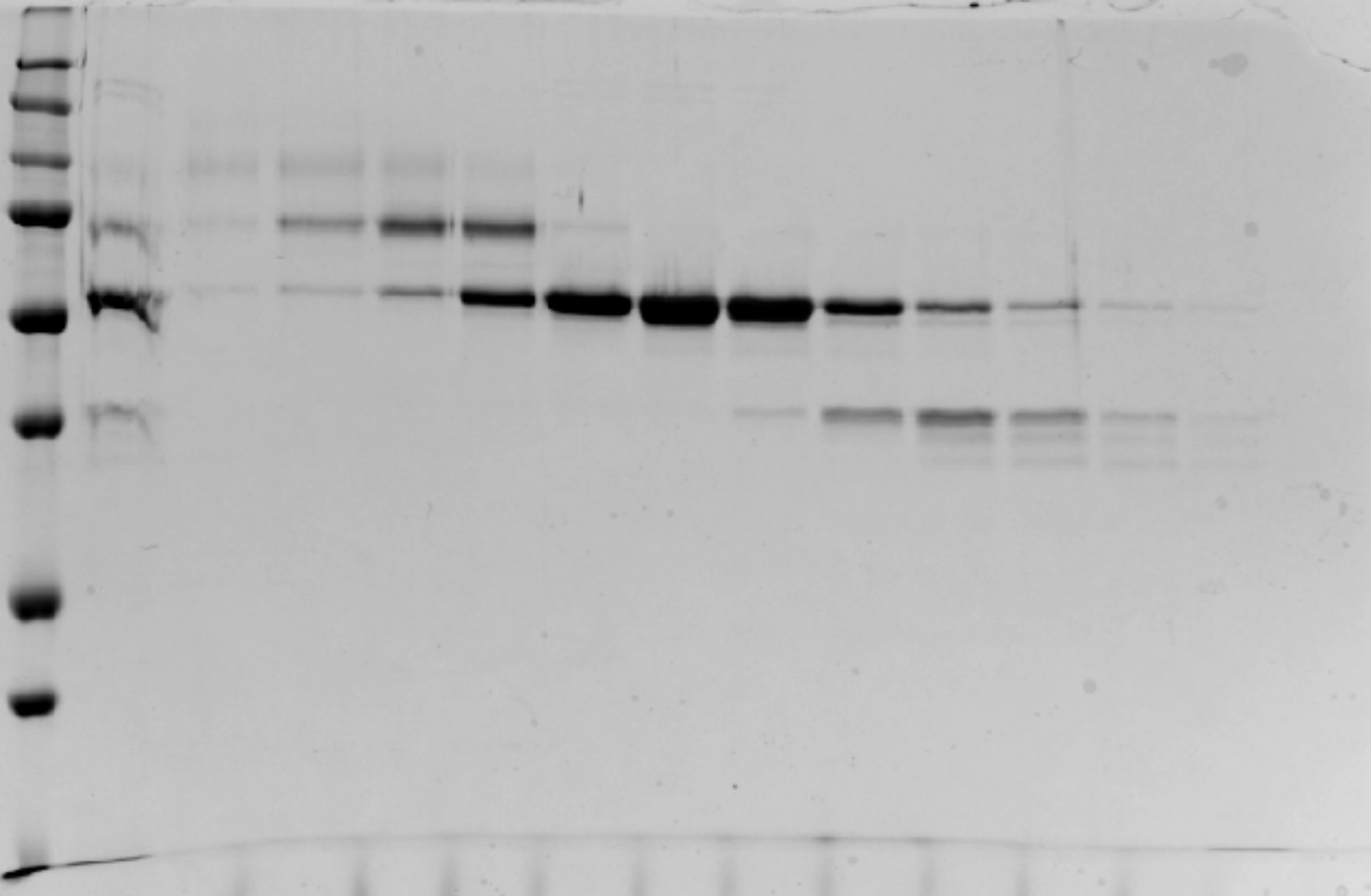

Supplement: Source data 1. [file elife-77424-data1.zip › Source data/Supplementary file 2/12-(B) Any1-diUb purification.pdf]

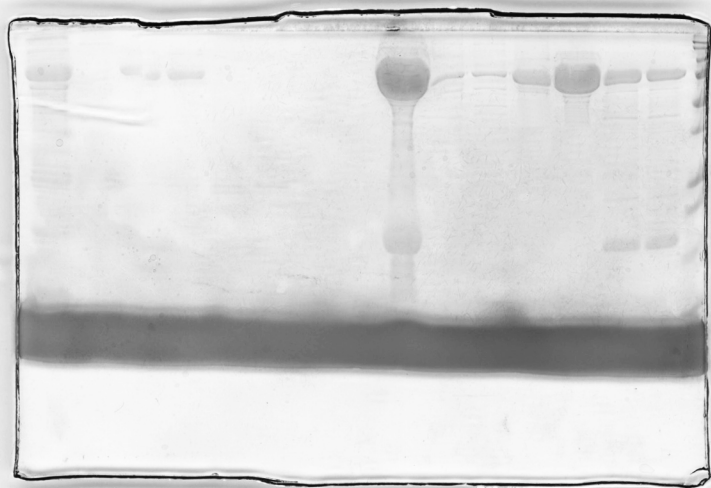

Supplement: Source data 1. [file elife-77424-data1.zip › Source data/Supplementary file 2/7-Rsp5 ww1-HECT domain purification.pdf]

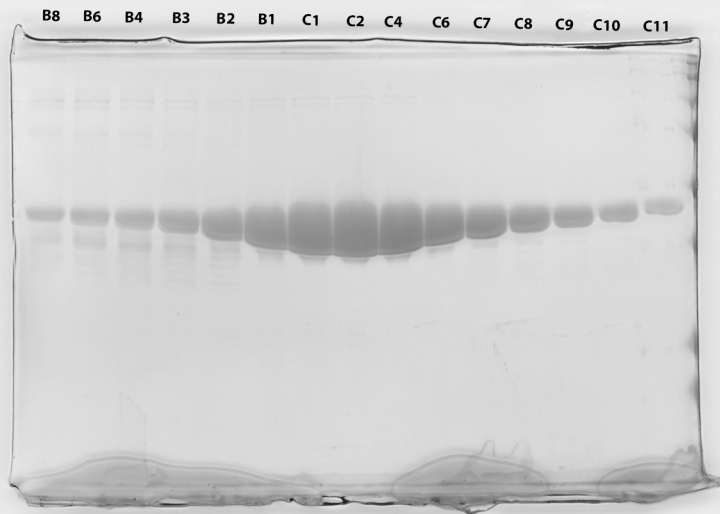

Supplement: Source data 1. [file elife-77424-data1.zip › Source data/Supplementary file 2/6-Rsp5 HECT domain purification.pdf]

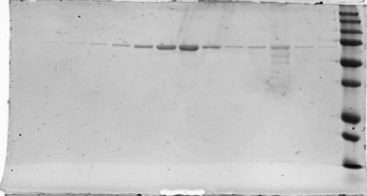

Supplement: Source data 1. [file elife-77424-data1.zip › Source data/Supplementary file 2/10-Pub1.pdf]

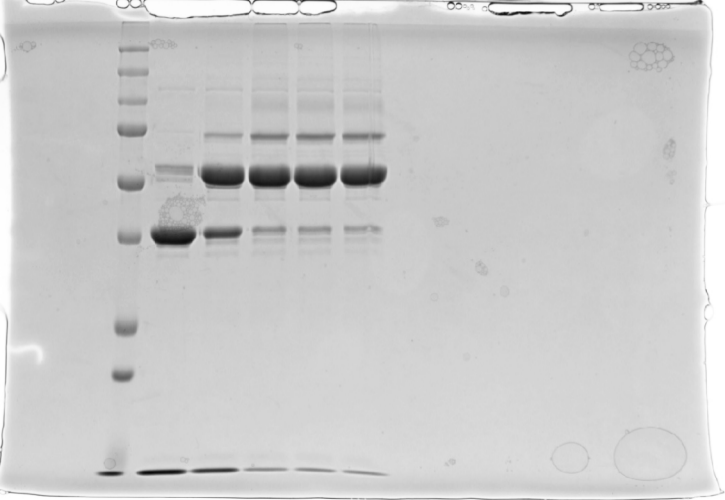

Supplement: Source data 1. [file elife-77424-data1.zip › Source data/Supplementary file 2/12-(A) Any1-diUb synthesis.pdf]

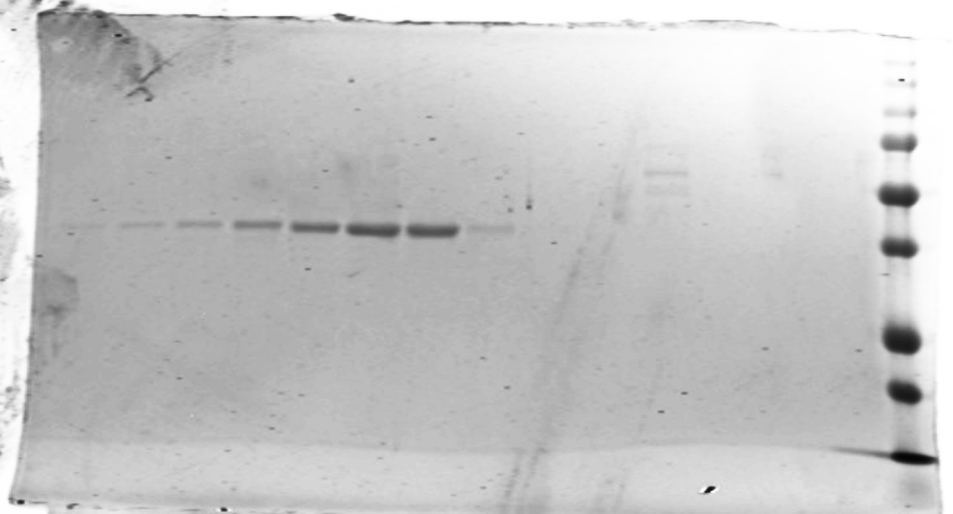

Supplement: Source data 1. [file elife-77424-data1.zip › Source data/Supplementary file 2/11-Any1.pdf]

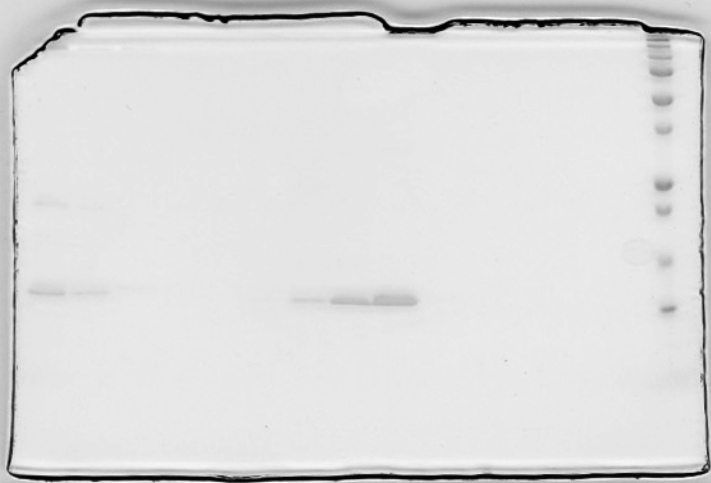

Supplement: Source data 1. [file elife-77424-data1.zip › Source data/Supplementary file 2/9-(A) Art5PY-purification.pdf]

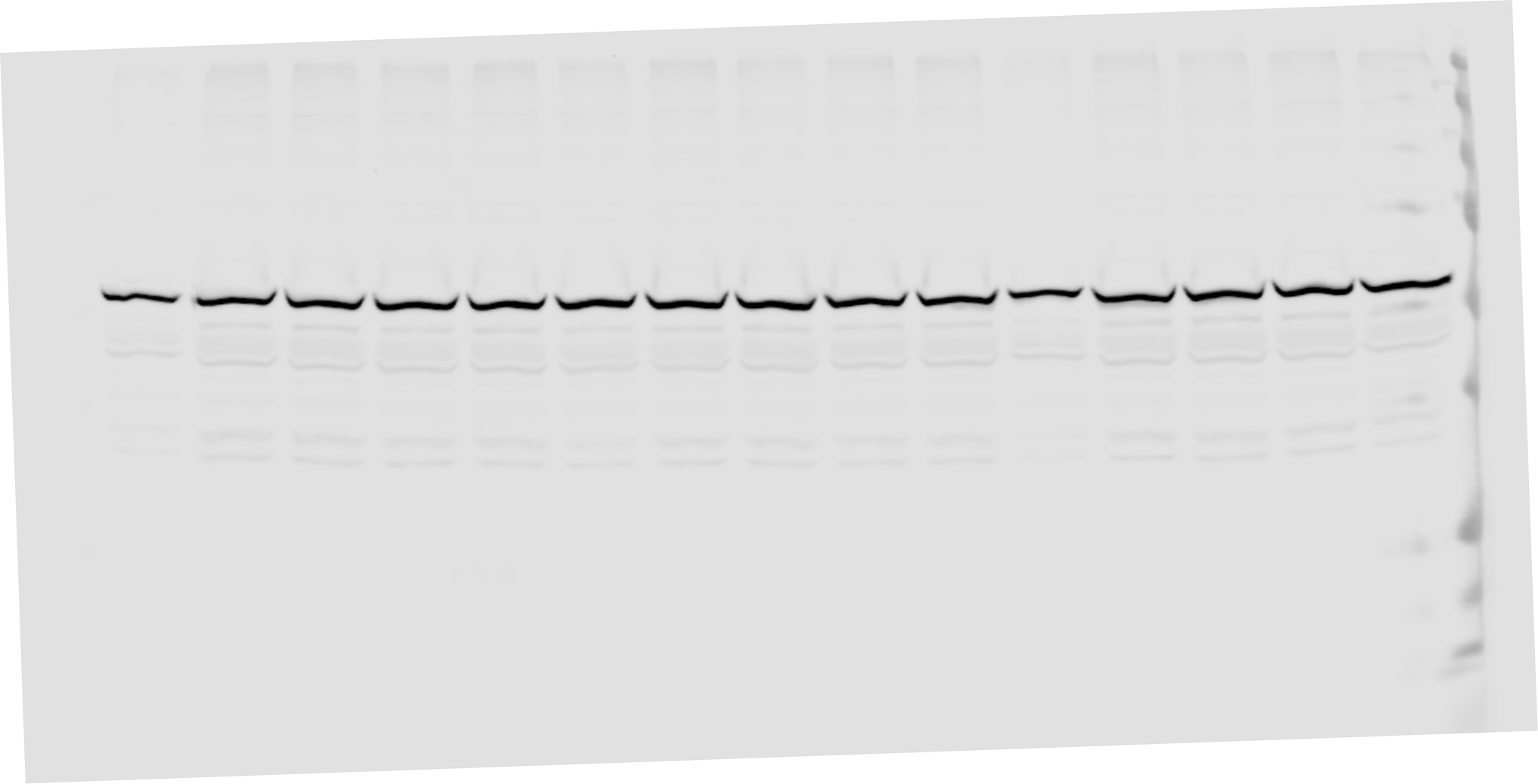

Supplement: Source data 1. [file elife-77424-data1.zip › Source data/Figure 2-figure supplemental 3/figure 2-figure supplemental 3A-G6PDH.tif]

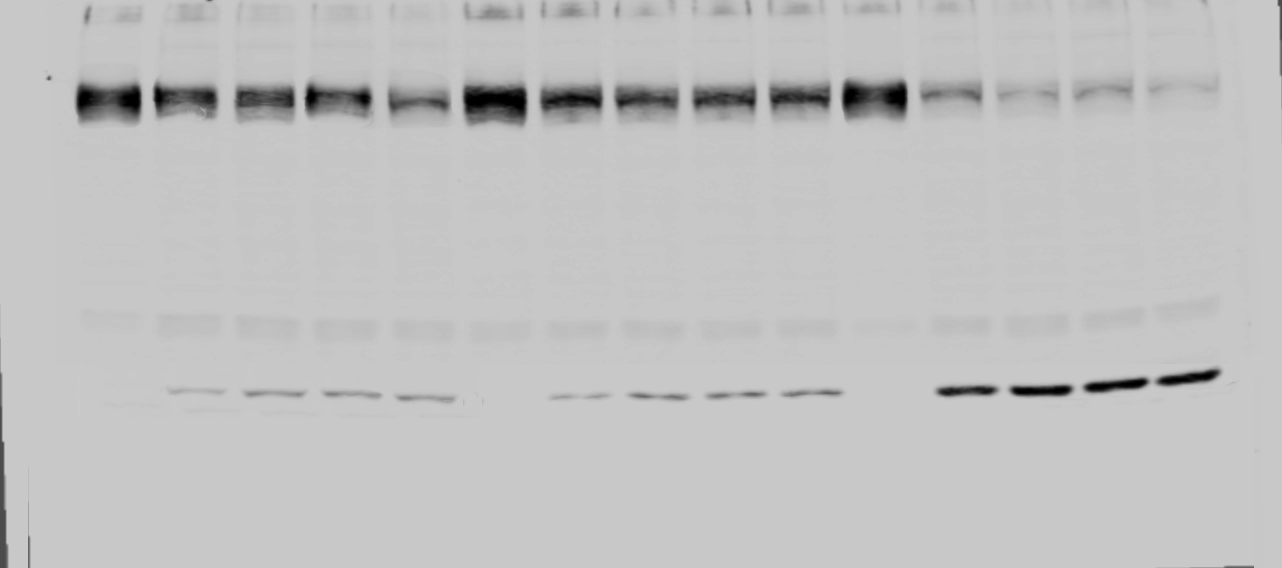

Supplement: Source data 1. [file elife-77424-data1.zip › Source data/Figure 2-figure supplemental 3/figure 2-figure supplemental 3A-GFP.tif]

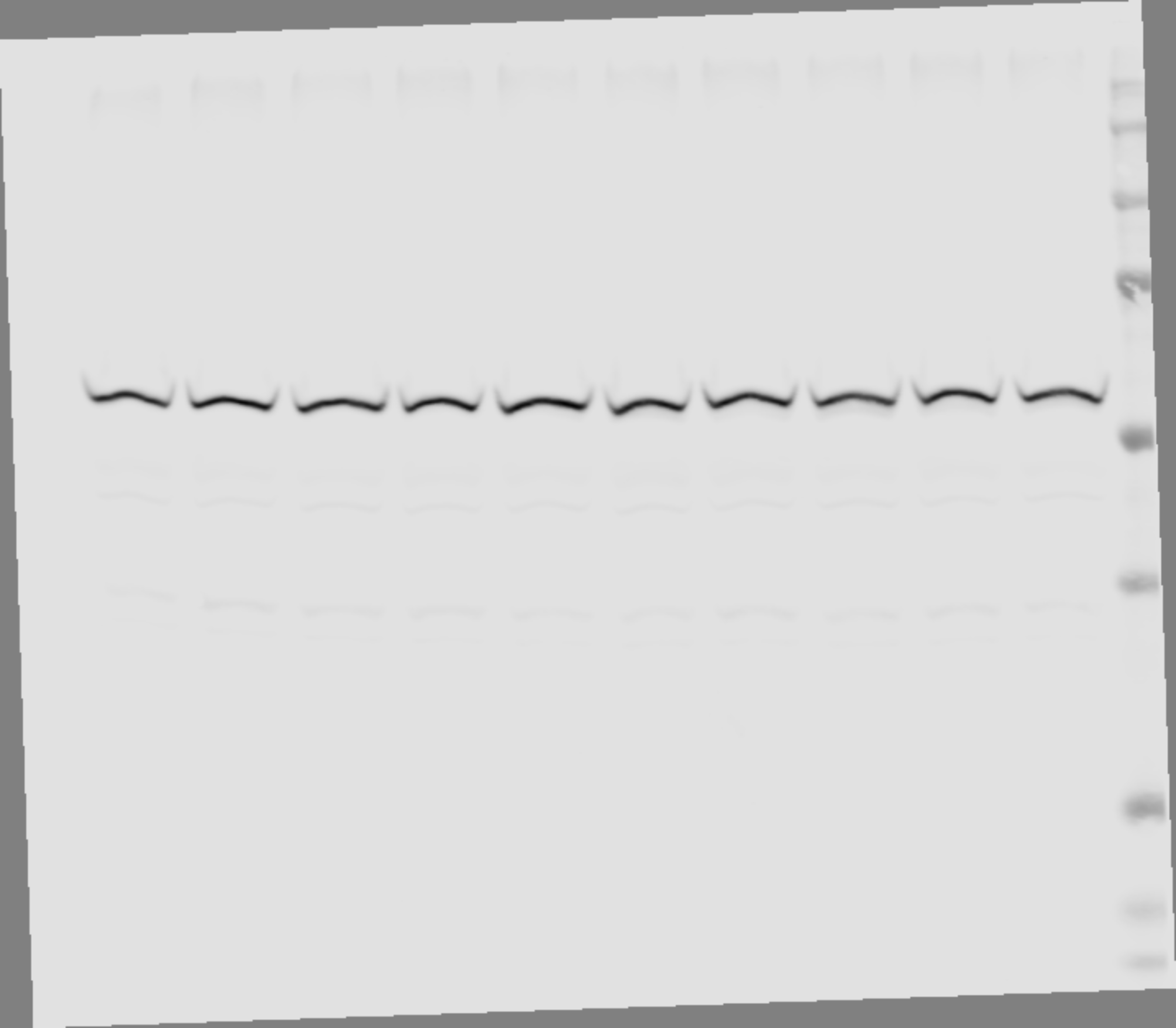

Supplement: Source data 1. [file elife-77424-data1.zip › Source data/Figure 2-figure supplemental 3/figure 2-figure supplemental 3C-G6PDH.tif]

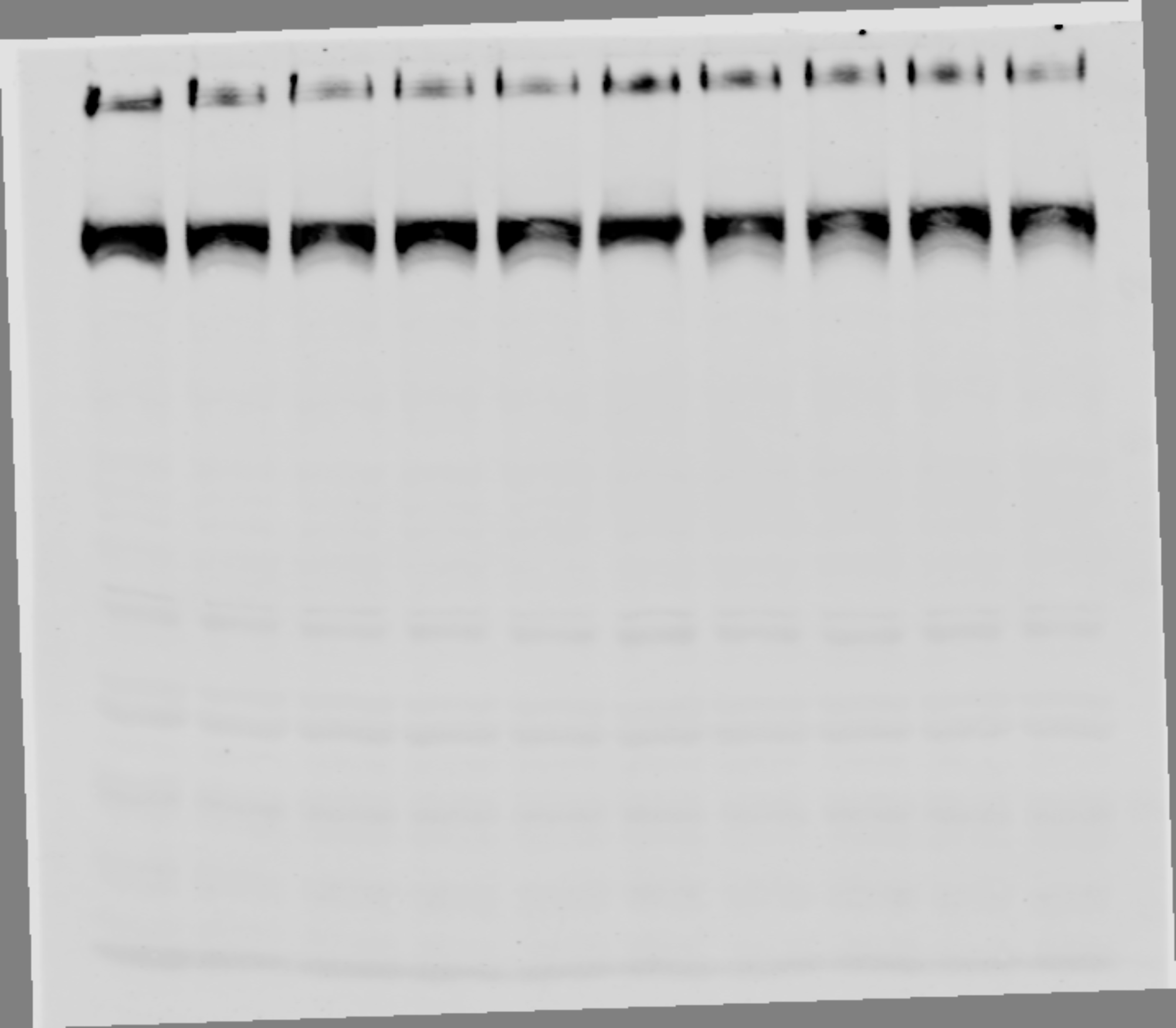

Supplement: Source data 1. [file elife-77424-data1.zip › Source data/Figure 2-figure supplemental 3/figure 2-figure supplemental 3C-GFP.tif]

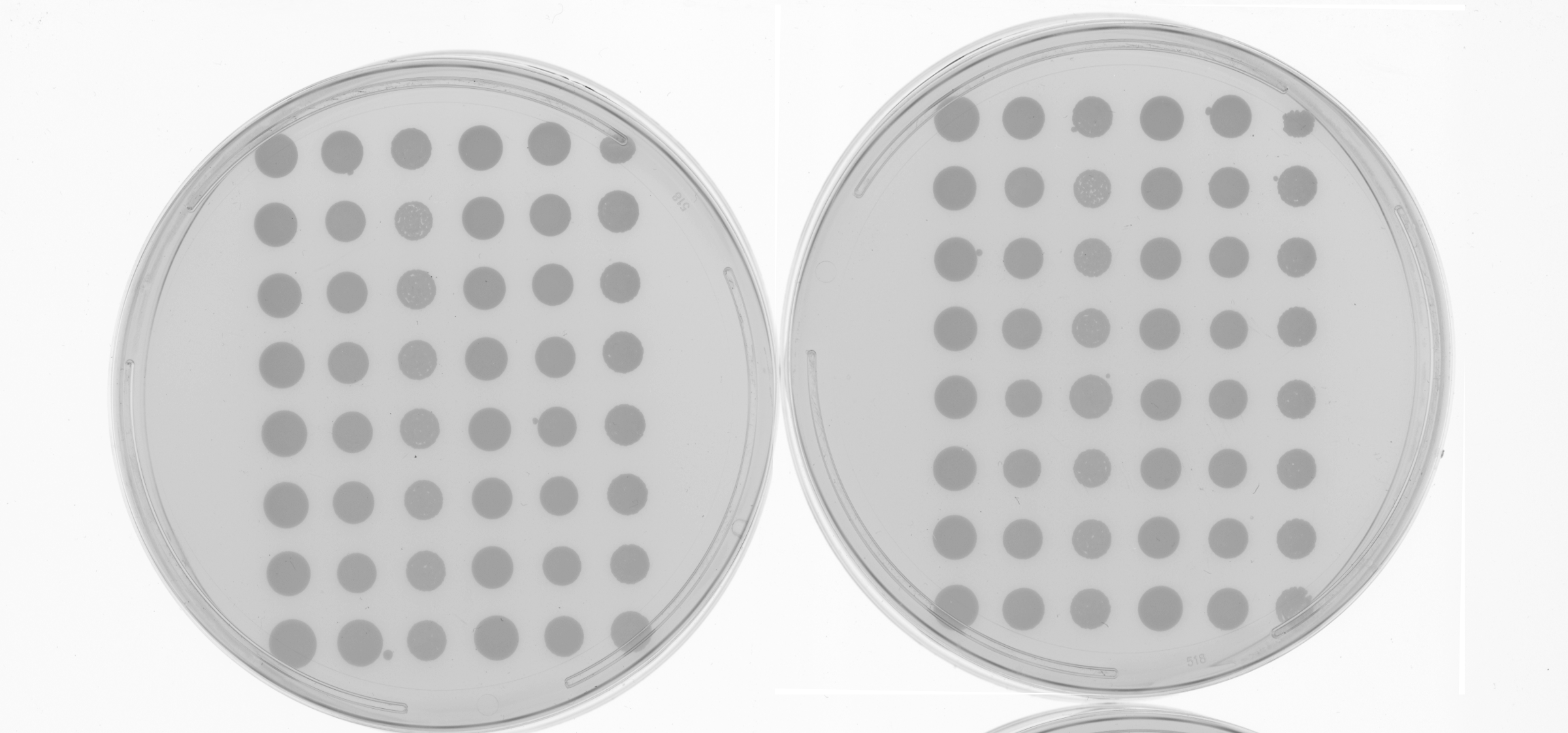

Supplement: Source data 1. [file elife-77424-data1.zip › Source data/Figure 2-figure supplemental 4/figure 2-figure supplemental 4A-0.tif]

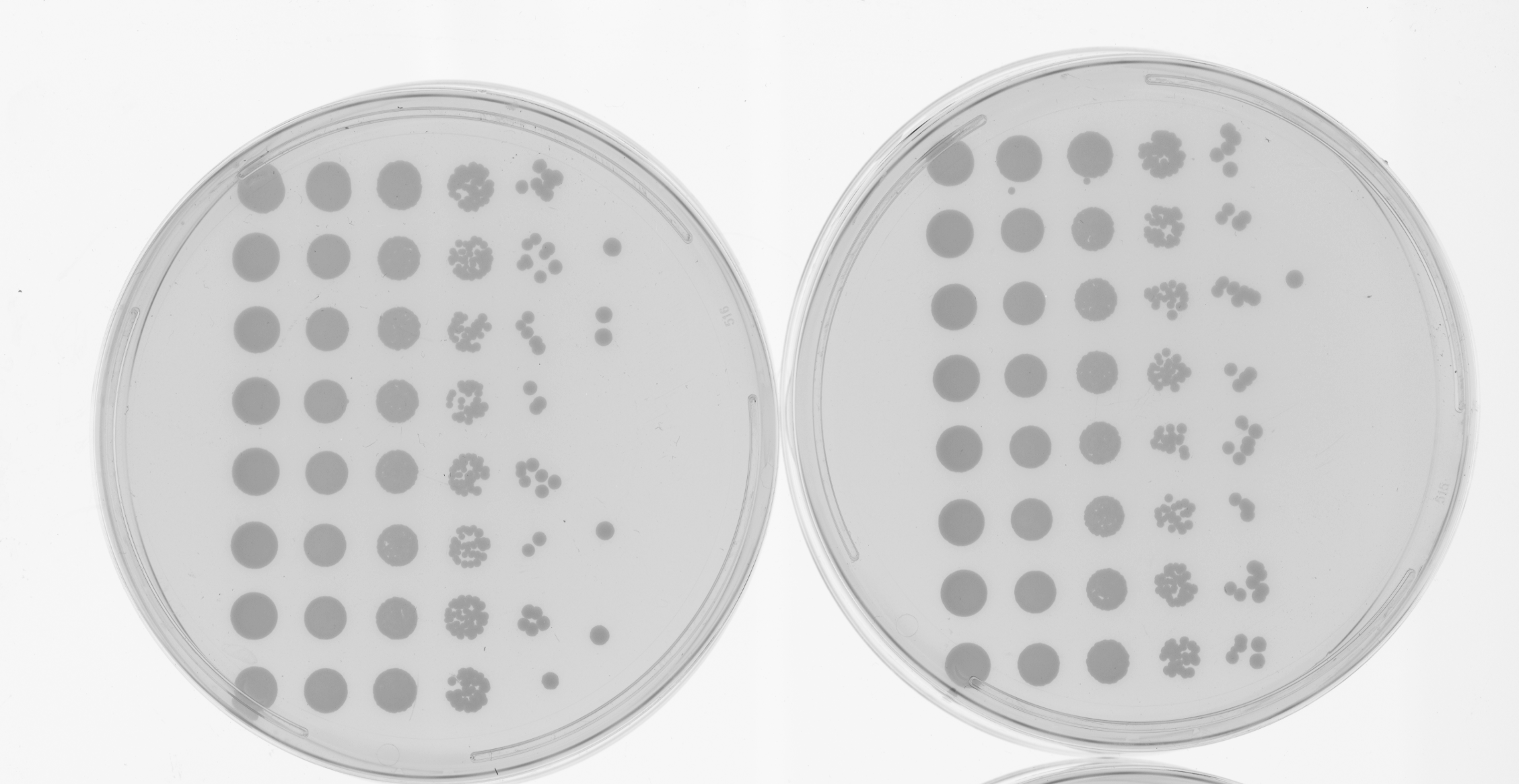

Supplement: Source data 1. [file elife-77424-data1.zip › Source data/Figure 2-figure supplemental 4/figure 2-figure supplemental 4B-0.tif]

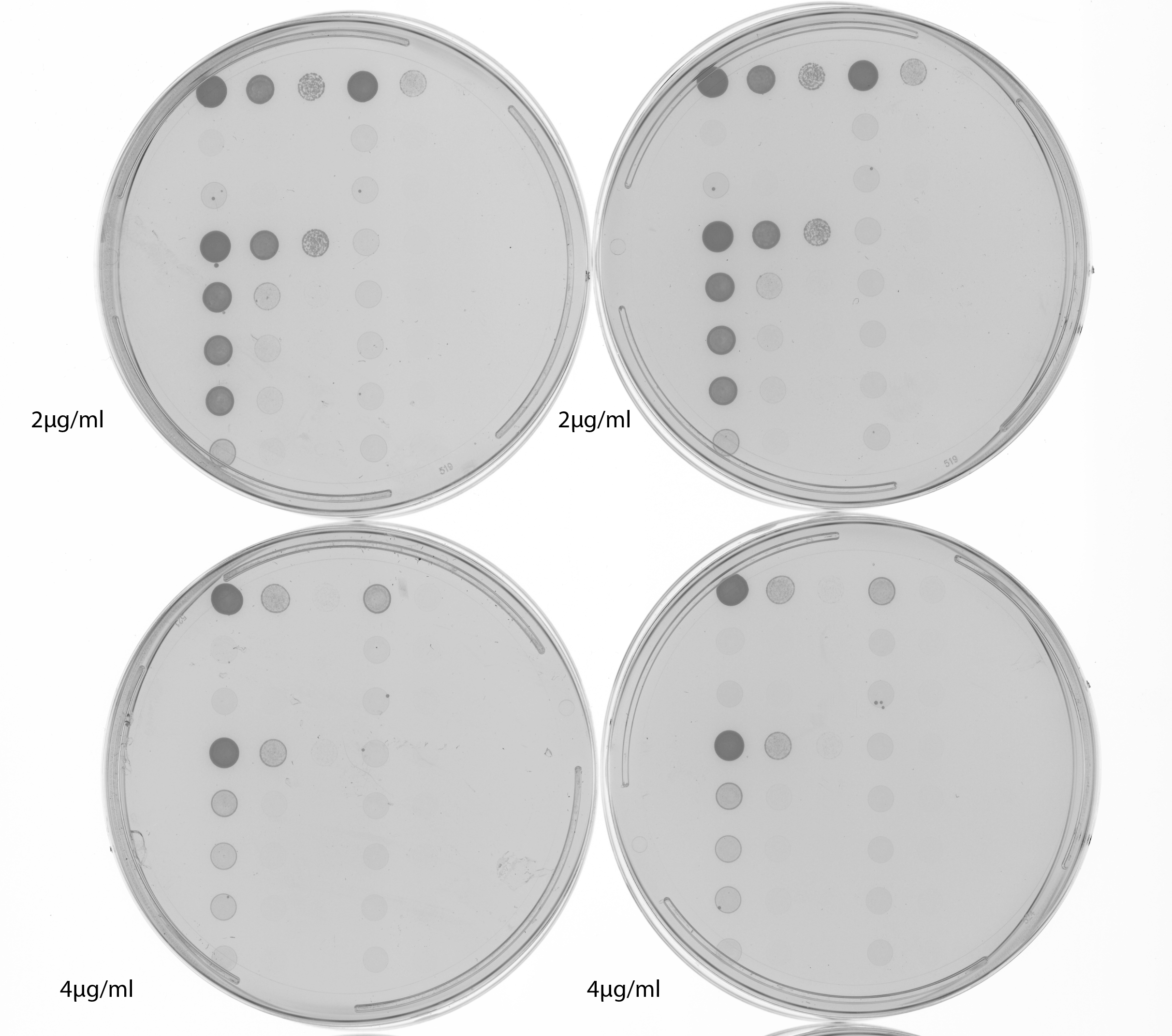

Supplement: Source data 1. [file elife-77424-data1.zip › Source data/Figure 2-figure supplemental 4/figure 2-figure supplemental 4A-2-4.tif]

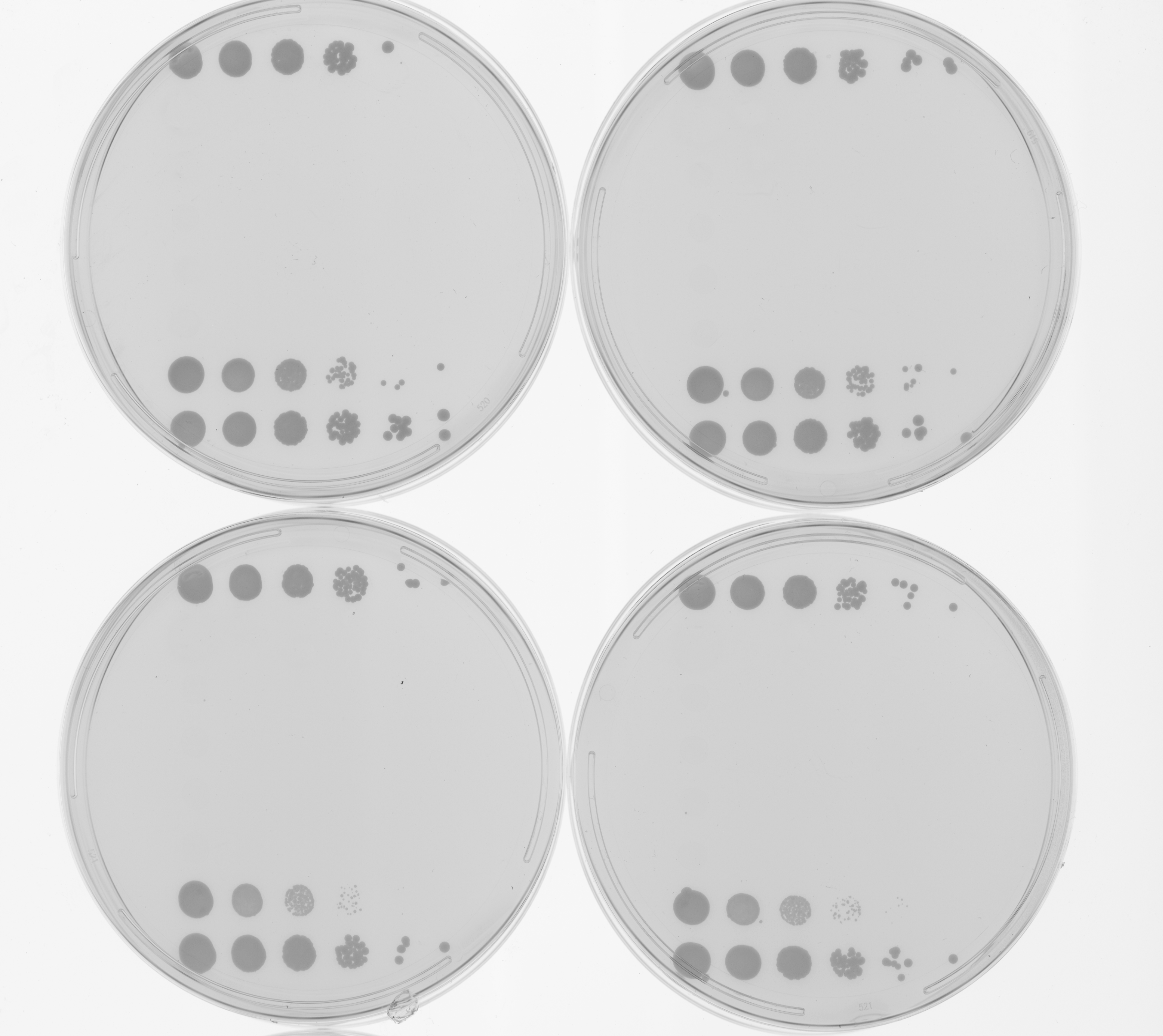

Supplement: Source data 1. [file elife-77424-data1.zip › Source data/Figure 2-figure supplemental 4/figure 2-figure supplemental 4B-2-4.tif]

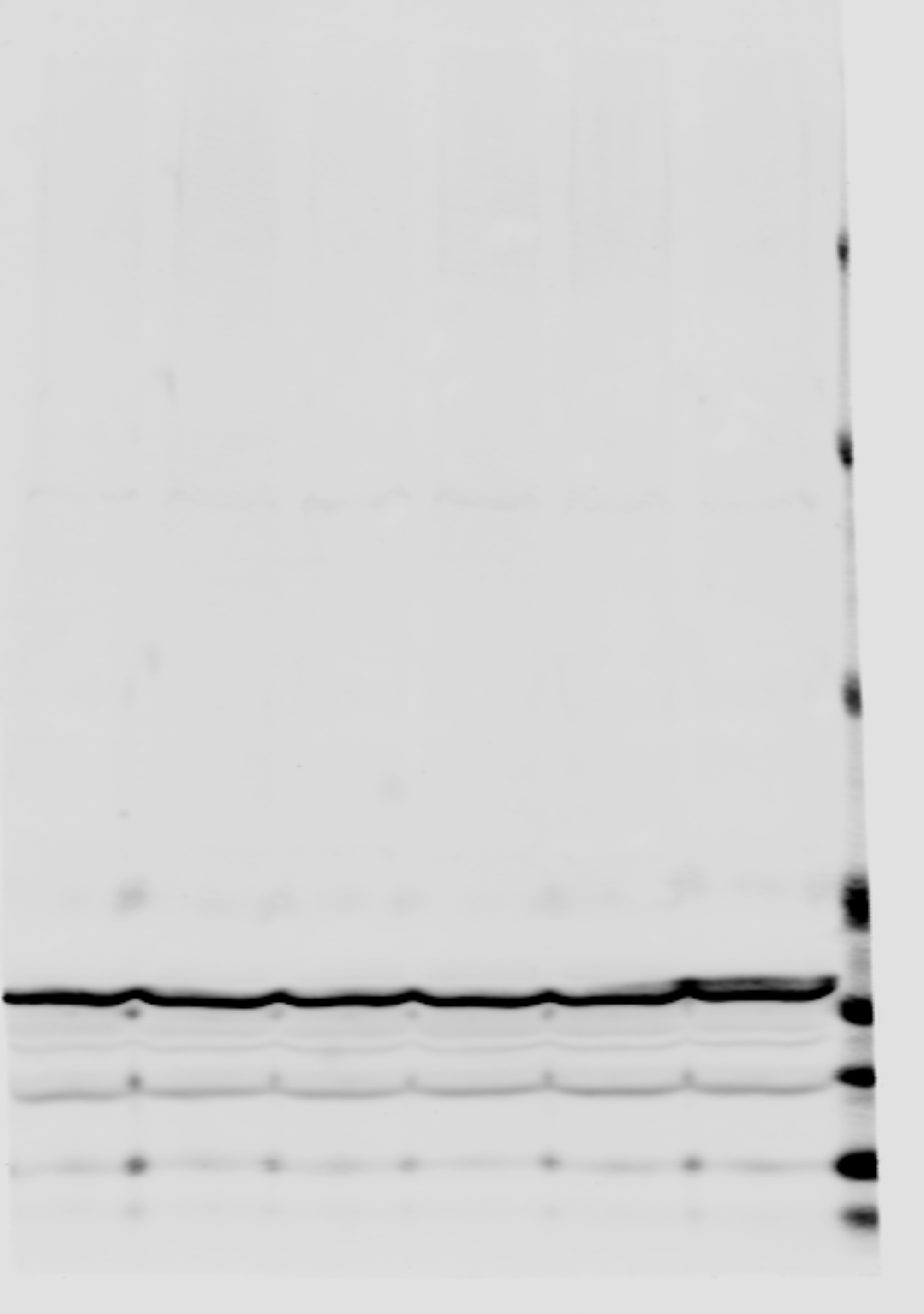

Supplement: Source data 1. [file elife-77424-data1.zip › Source data/Figure 2-figure supplemental 2/figure 2-figure supplemental 2B-G6PDH.tif]

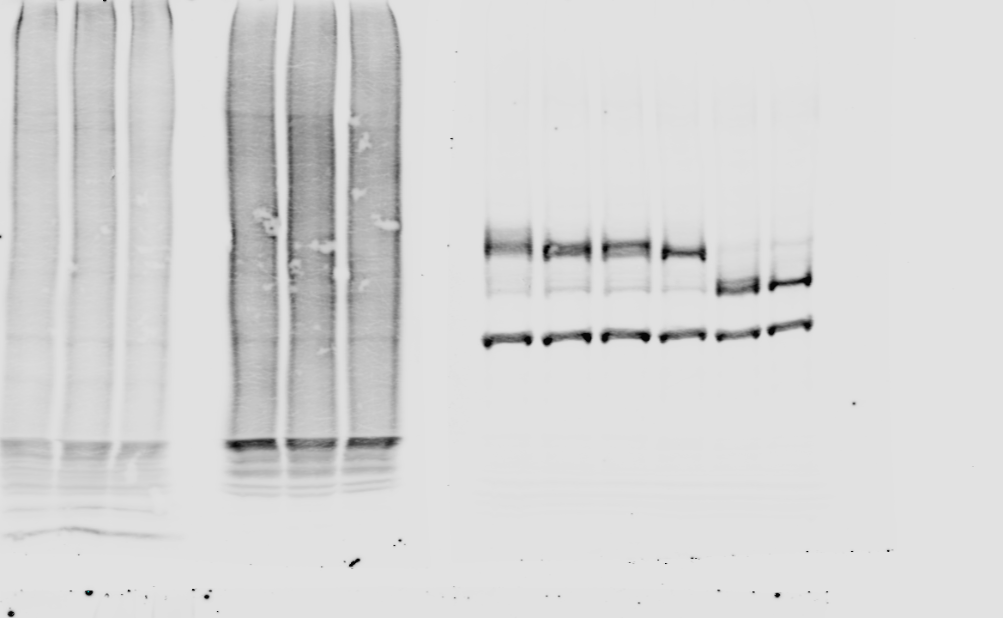

Supplement: Source data 1. [file elife-77424-data1.zip › Source data/Figure 2-figure supplemental 2/figure 2-figure supplemental 2A-FLAG.tif]

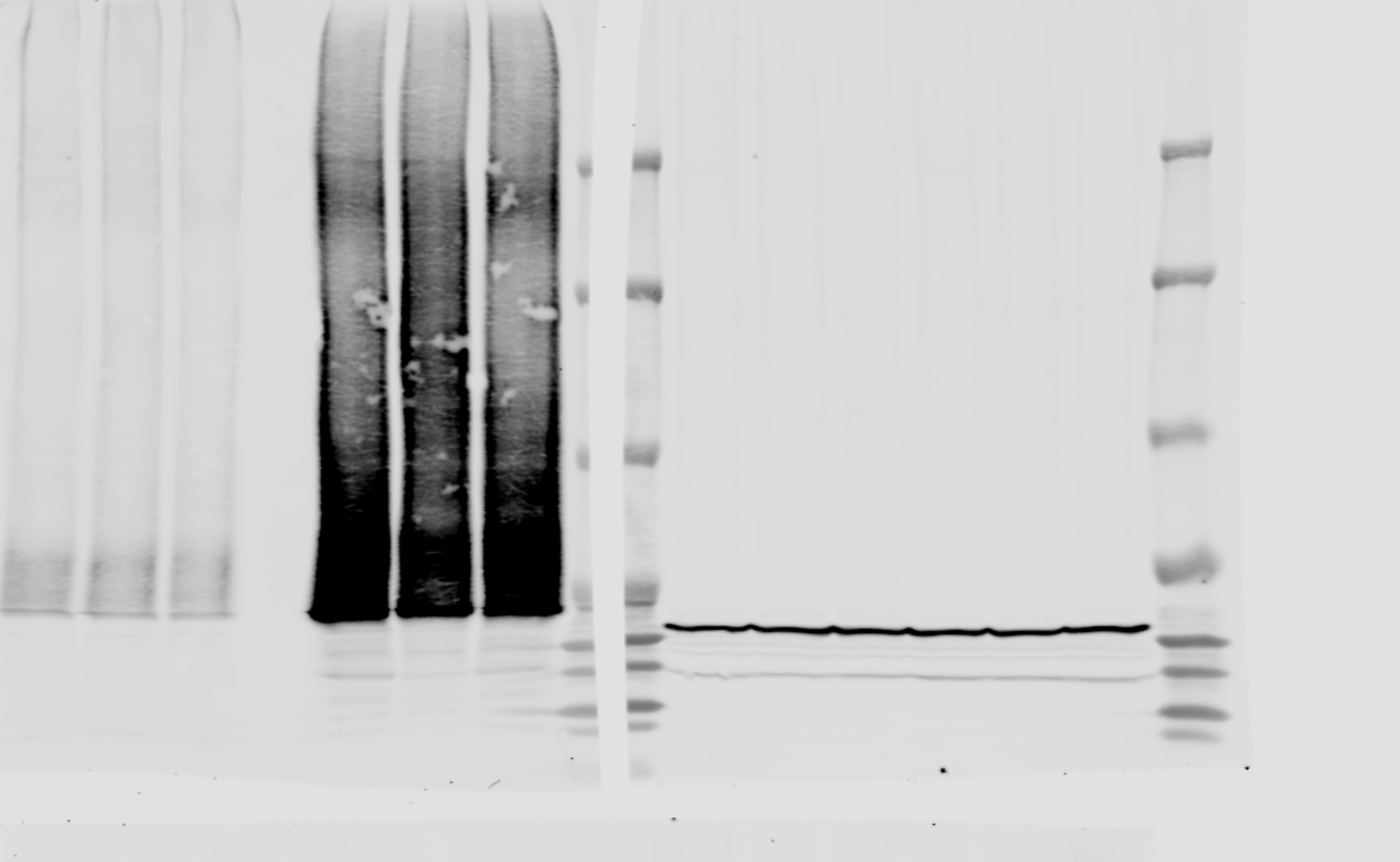

Supplement: Source data 1. [file elife-77424-data1.zip › Source data/Figure 2-figure supplemental 2/figure 2-figure supplemental 2A-G6PDH.tif]

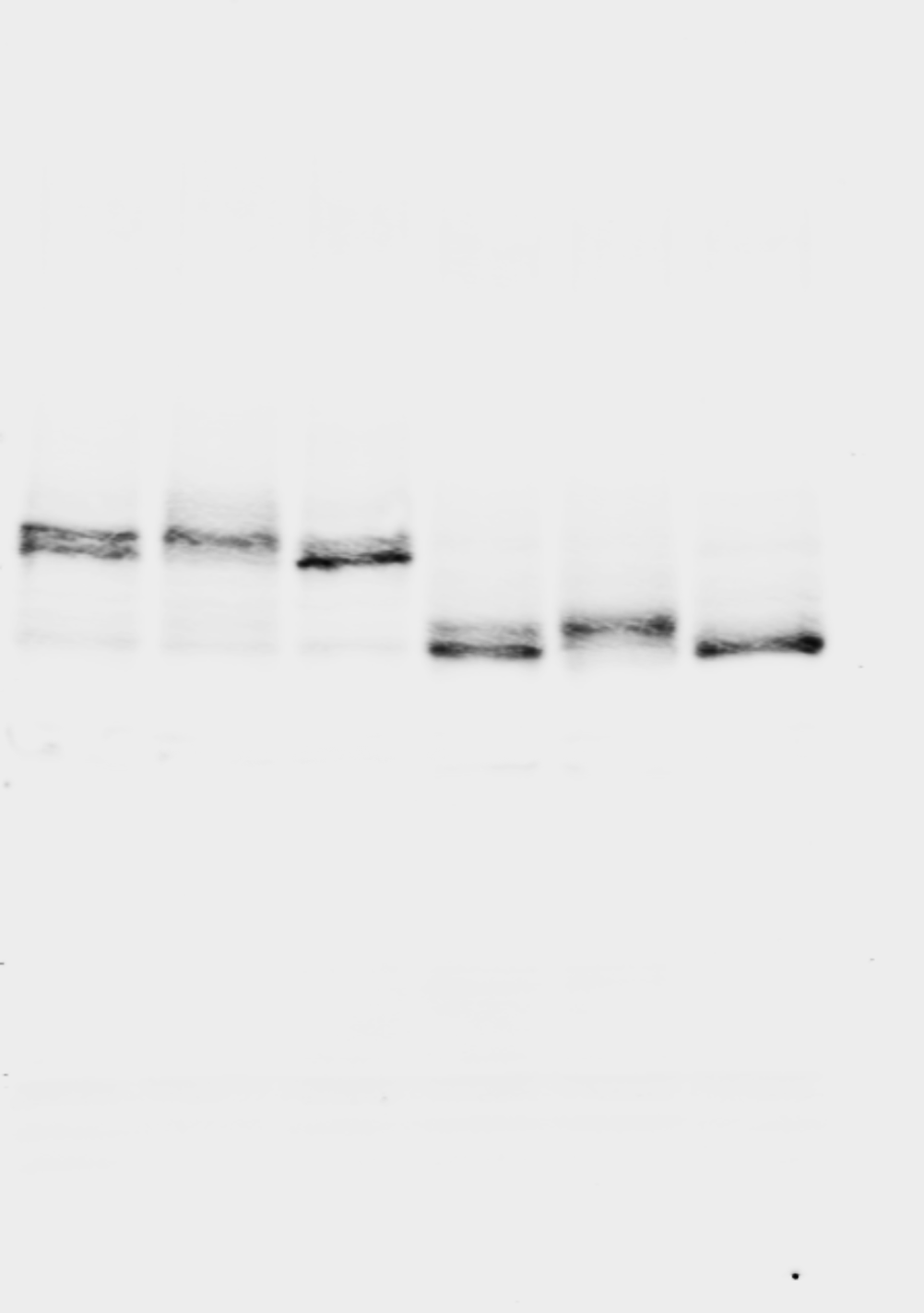

Supplement: Source data 1. [file elife-77424-data1.zip › Source data/Figure 2-figure supplemental 2/figure 2-figure supplemental 2B-FLAG.tif]

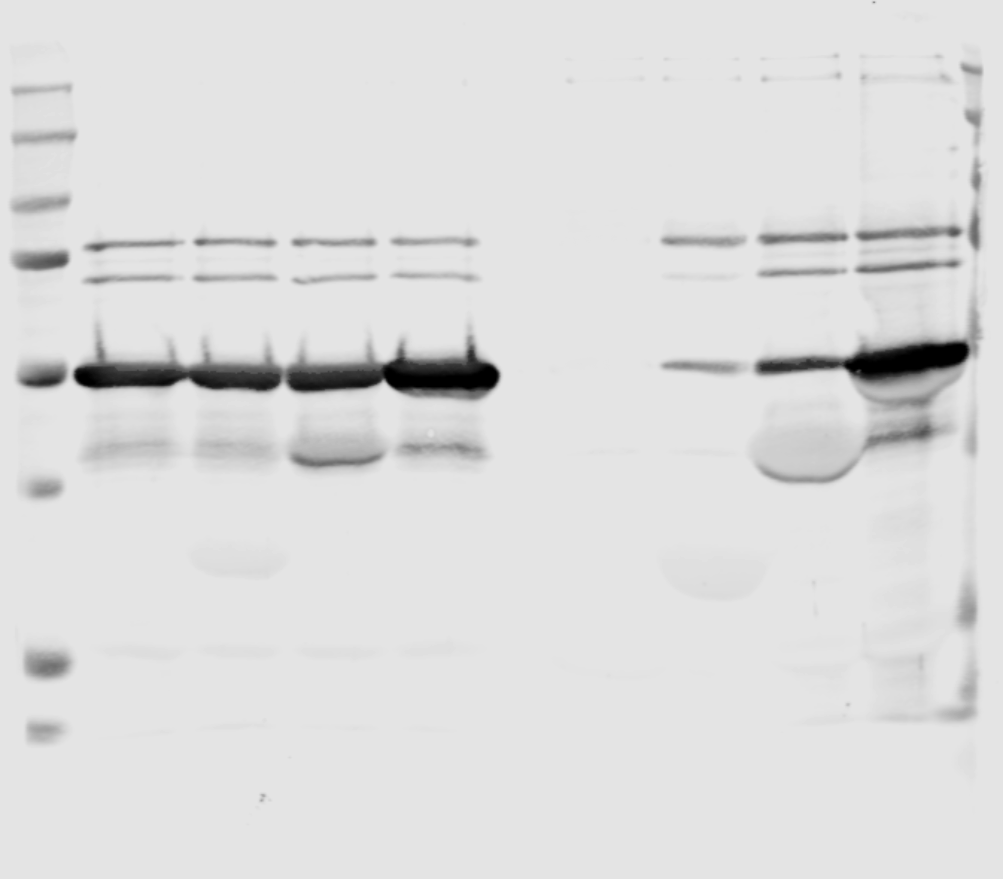

Supplement: Source data 1. [file elife-77424-data1.zip › Source data/Figure 4/Figure 4E-Rsp5.tif]

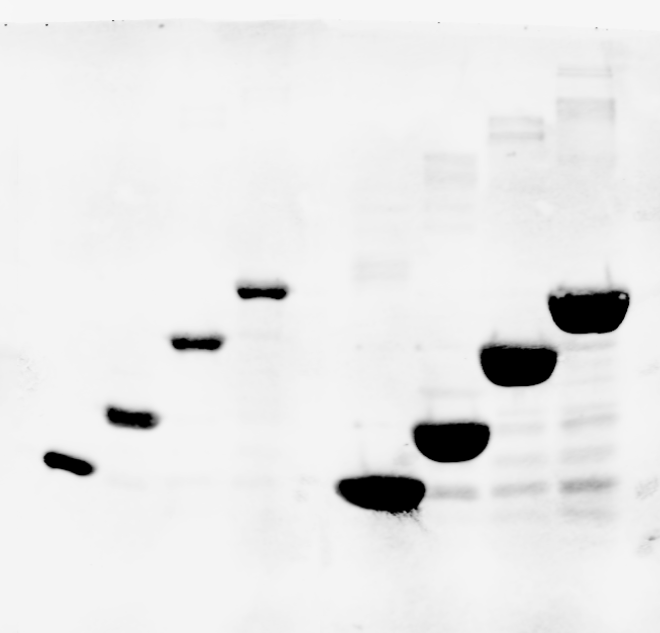

Supplement: Source data 1. [file elife-77424-data1.zip › Source data/Figure 4/Figure 4E-GST.tif]

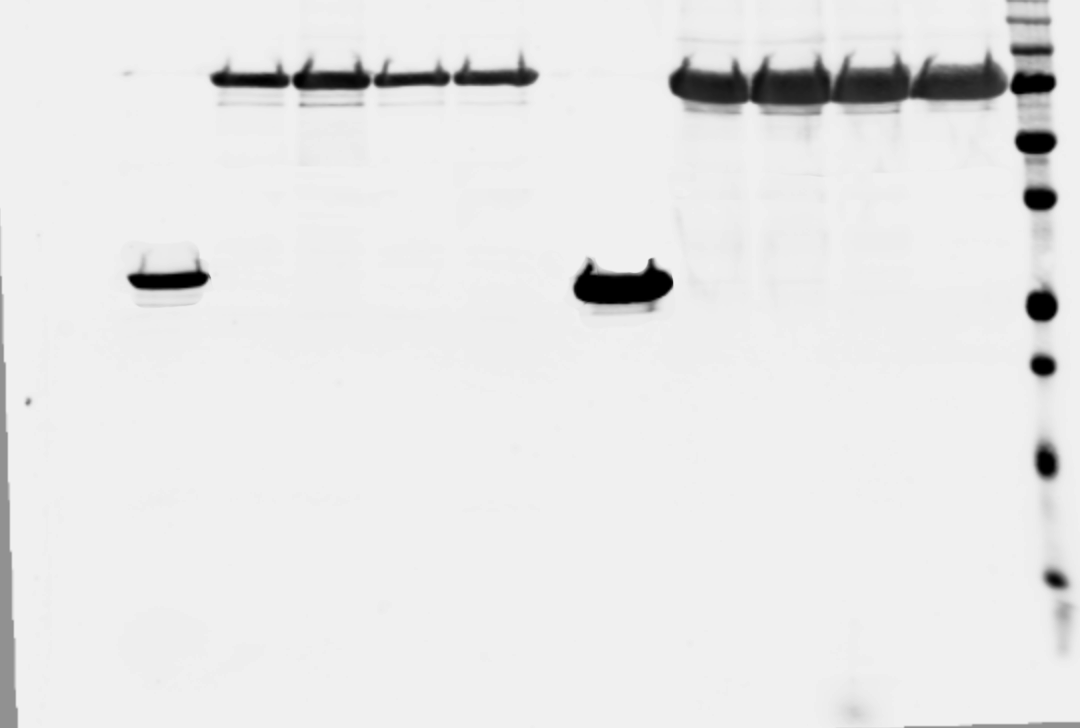

Supplement: Source data 1. [file elife-77424-data1.zip › Source data/Figure 4/Figure 4A-GST.tif]

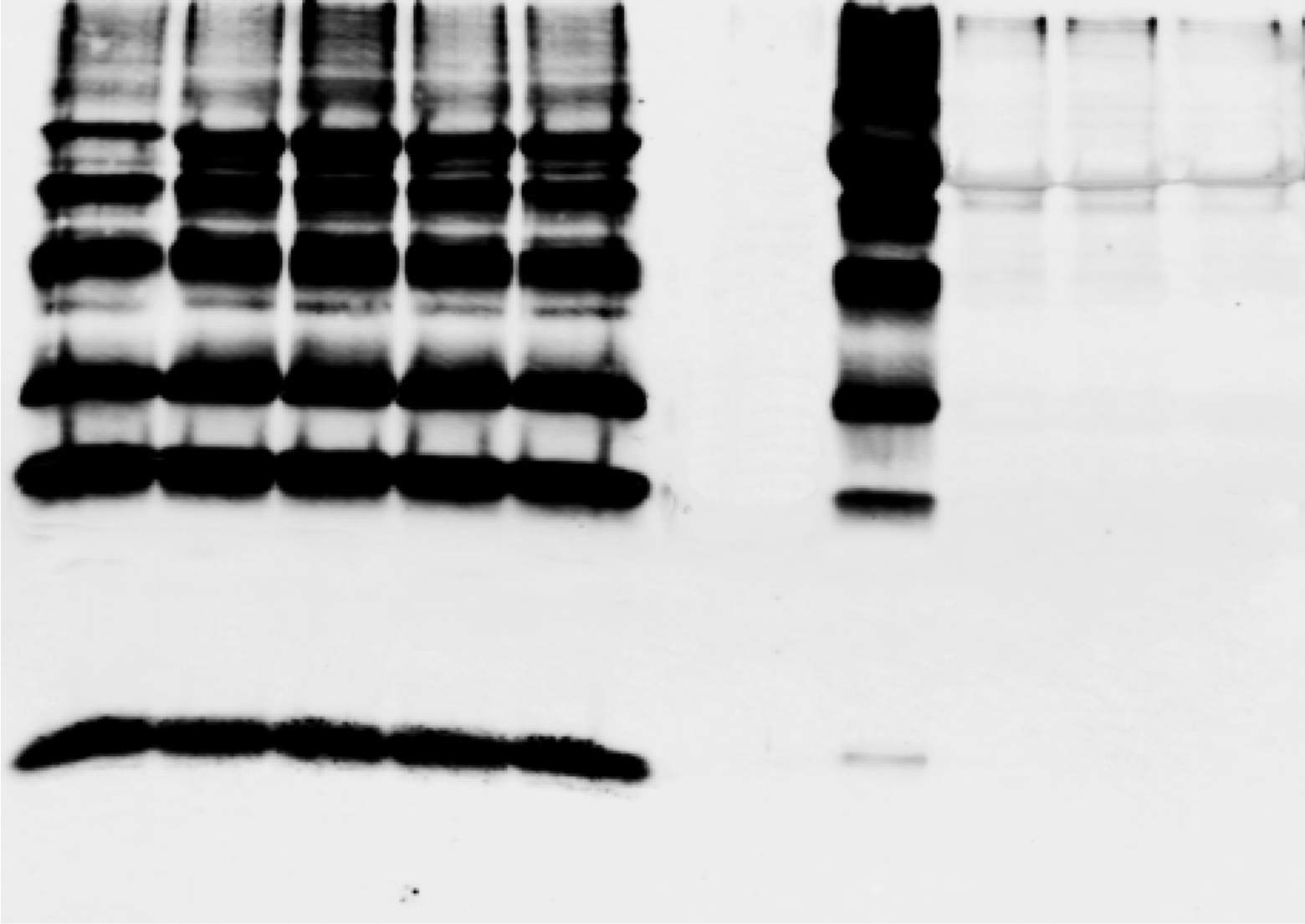

Supplement: Source data 1. [file elife-77424-data1.zip › Source data/Figure 4/Figure 4A-6xHis.tif]

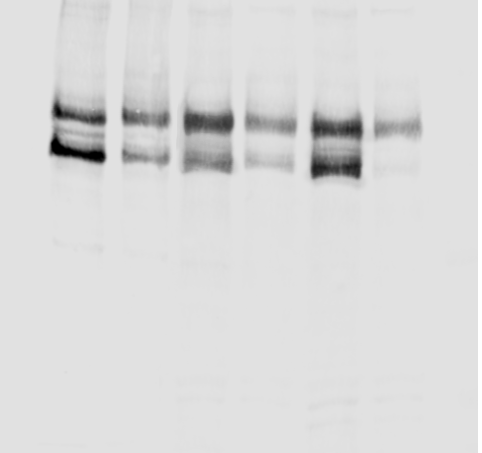

Supplement: Source data 1. [file elife-77424-data1.zip › Source data/Figure 6-figure supplemental 2/figure 6-figure supplemental 2B-FLAG.tif]

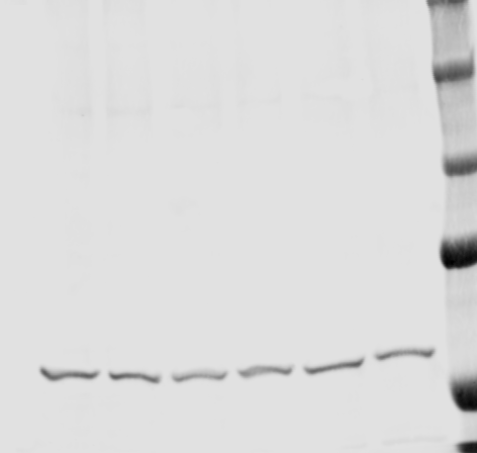

Supplement: Source data 1. [file elife-77424-data1.zip › Source data/Figure 6-figure supplemental 2/figure 6-figure supplemental 2B-G6PDH.tif]

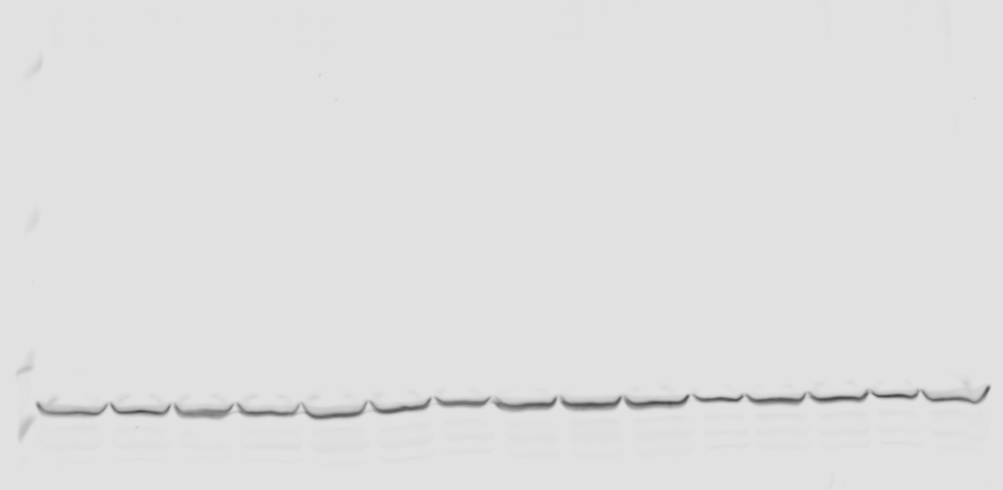

Supplement: Source data 1. [file elife-77424-data1.zip › Source data/Figure 6-figure supplemental 2/figure 6-figure supplemental 2A-G6PDH.tif]

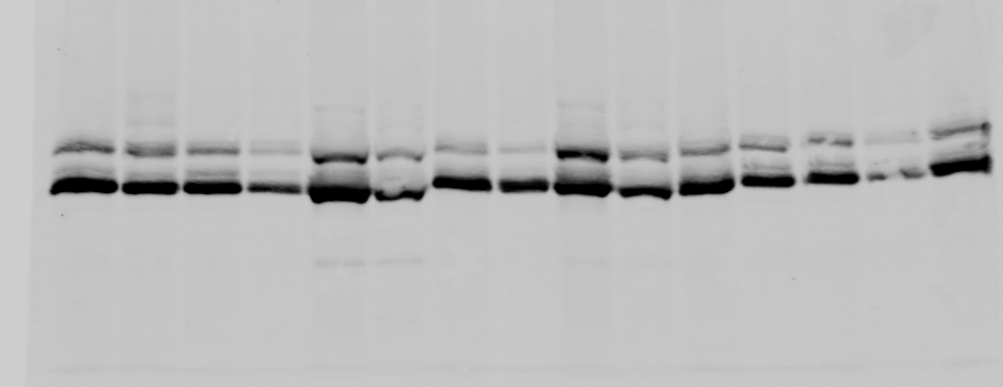

Supplement: Source data 1. [file elife-77424-data1.zip › Source data/Figure 6-figure supplemental 2/figure 6-figure supplemental 2A-FLAG.tif]

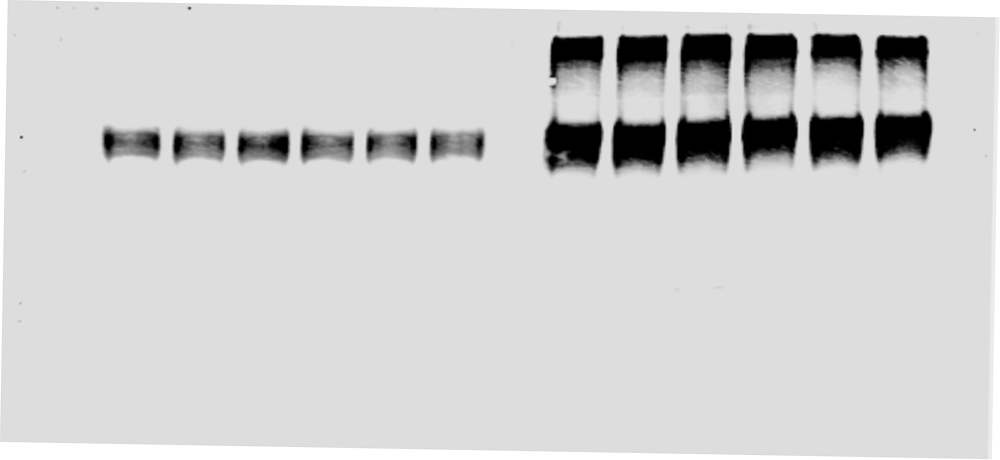

Supplement: Source data 1. [file elife-77424-data1.zip › Source data/Figure 2/figure 2E-GFP_new_v3.tif]

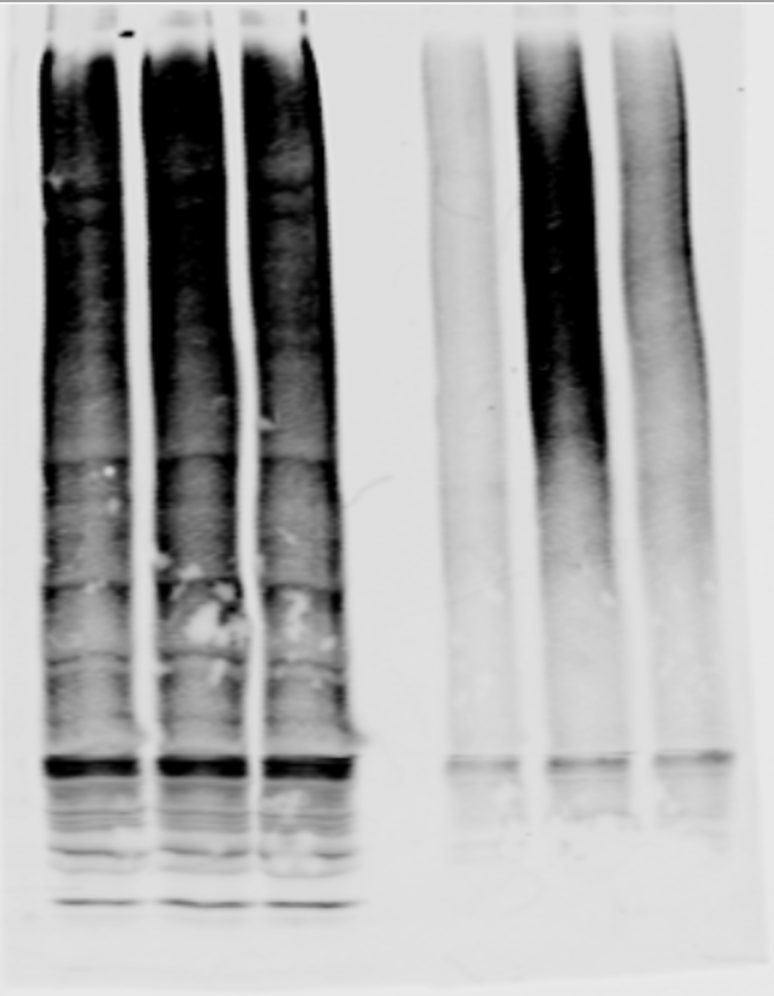

Supplement: Source data 1. [file elife-77424-data1.zip › Source data/Figure 2/figure 2D-myc_new.tif]

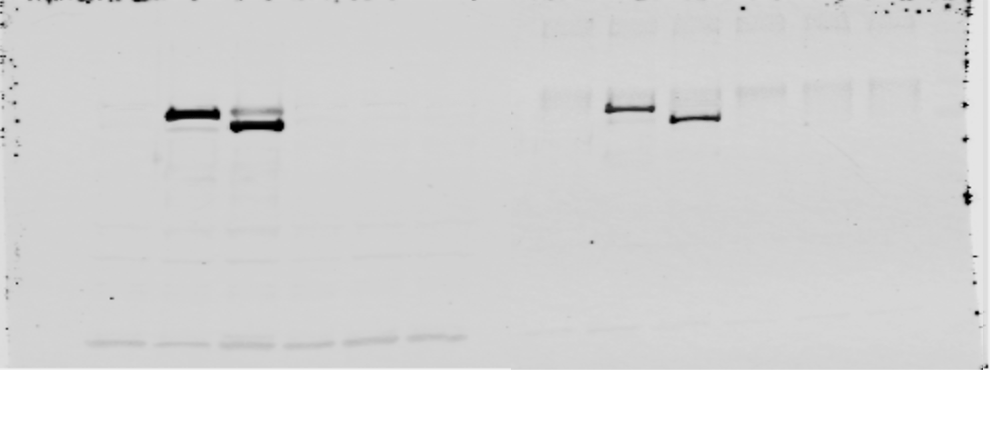

Supplement: Source data 1. [file elife-77424-data1.zip › Source data/Figure 2/figure 2E-HA-new.tif]

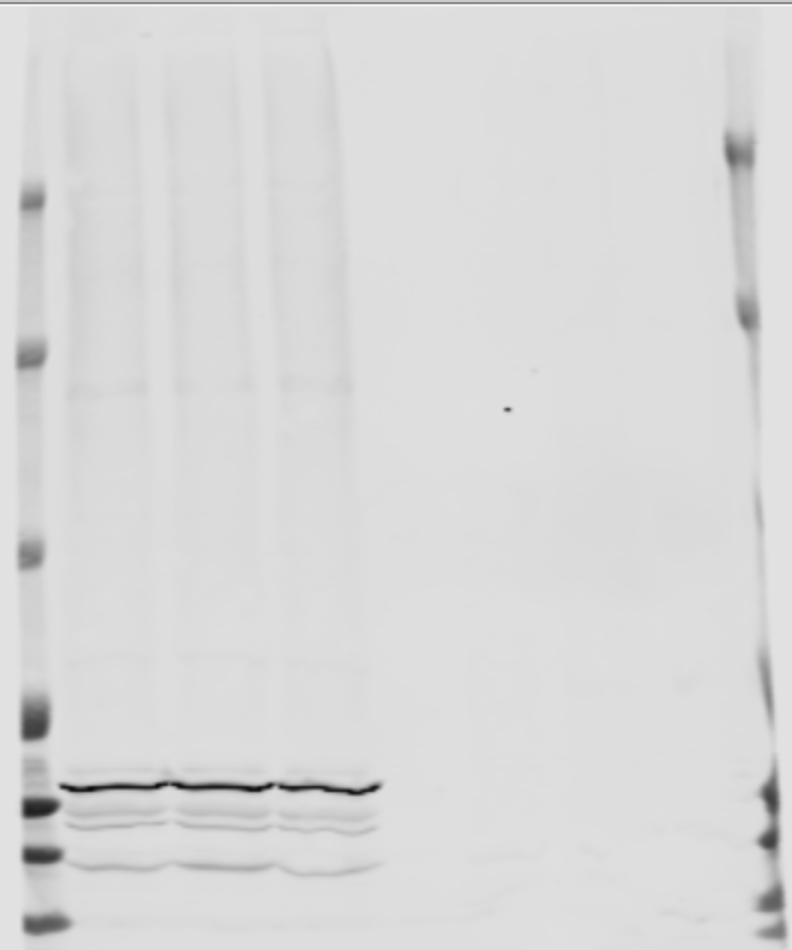

Supplement: Source data 1. [file elife-77424-data1.zip › Source data/Figure 2/figure 2D-G6PDH_new.tif]

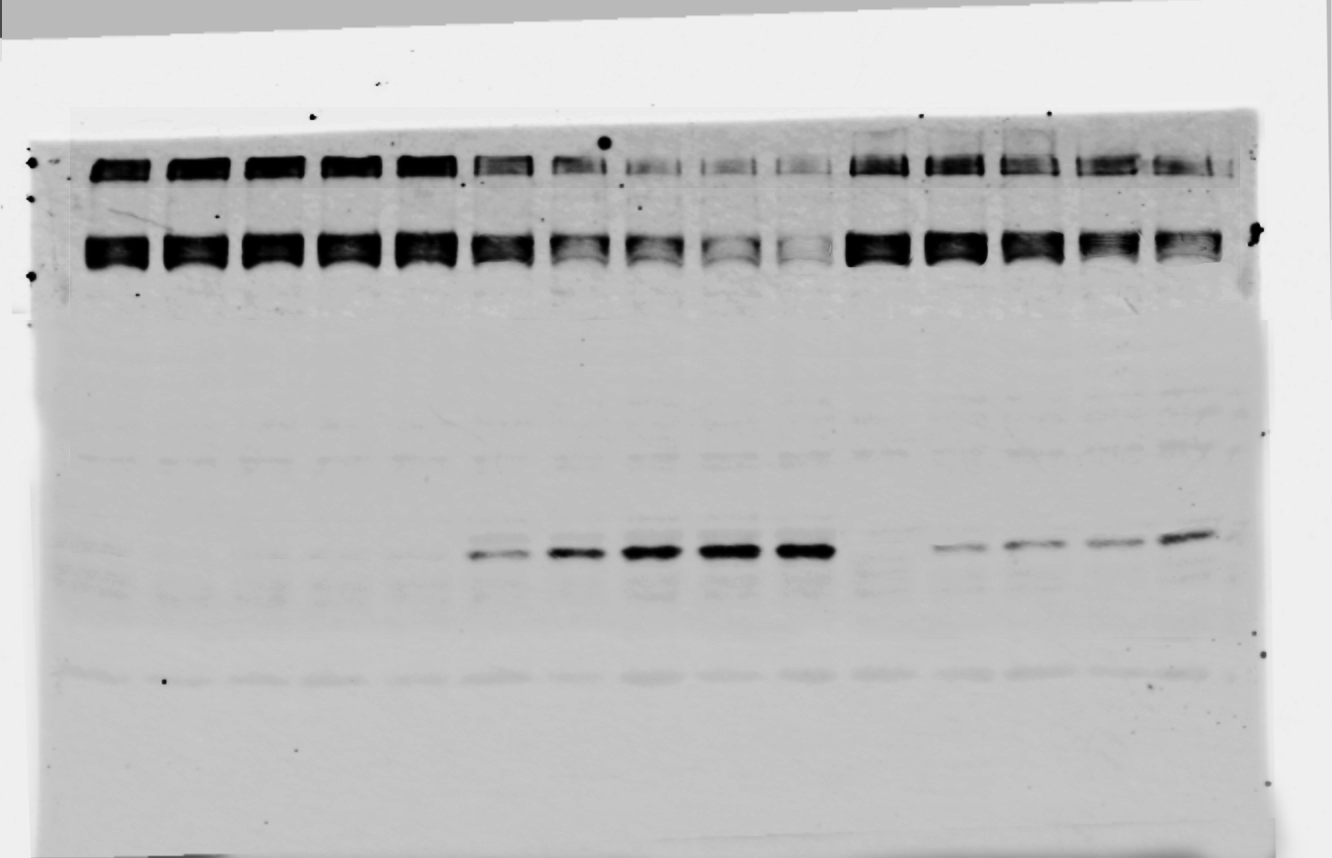

Supplement: Source data 1. [file elife-77424-data1.zip › Source data/Figure 2/Figure 2A_GFP.tif]

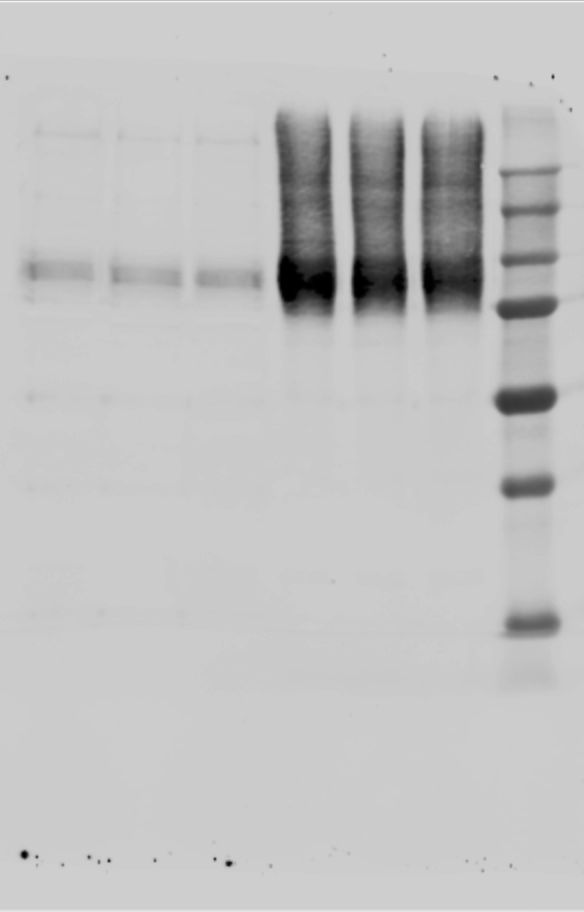

Supplement: Source data 1. [file elife-77424-data1.zip › Source data/Figure 2/figure 2D-GFP_new.tif]

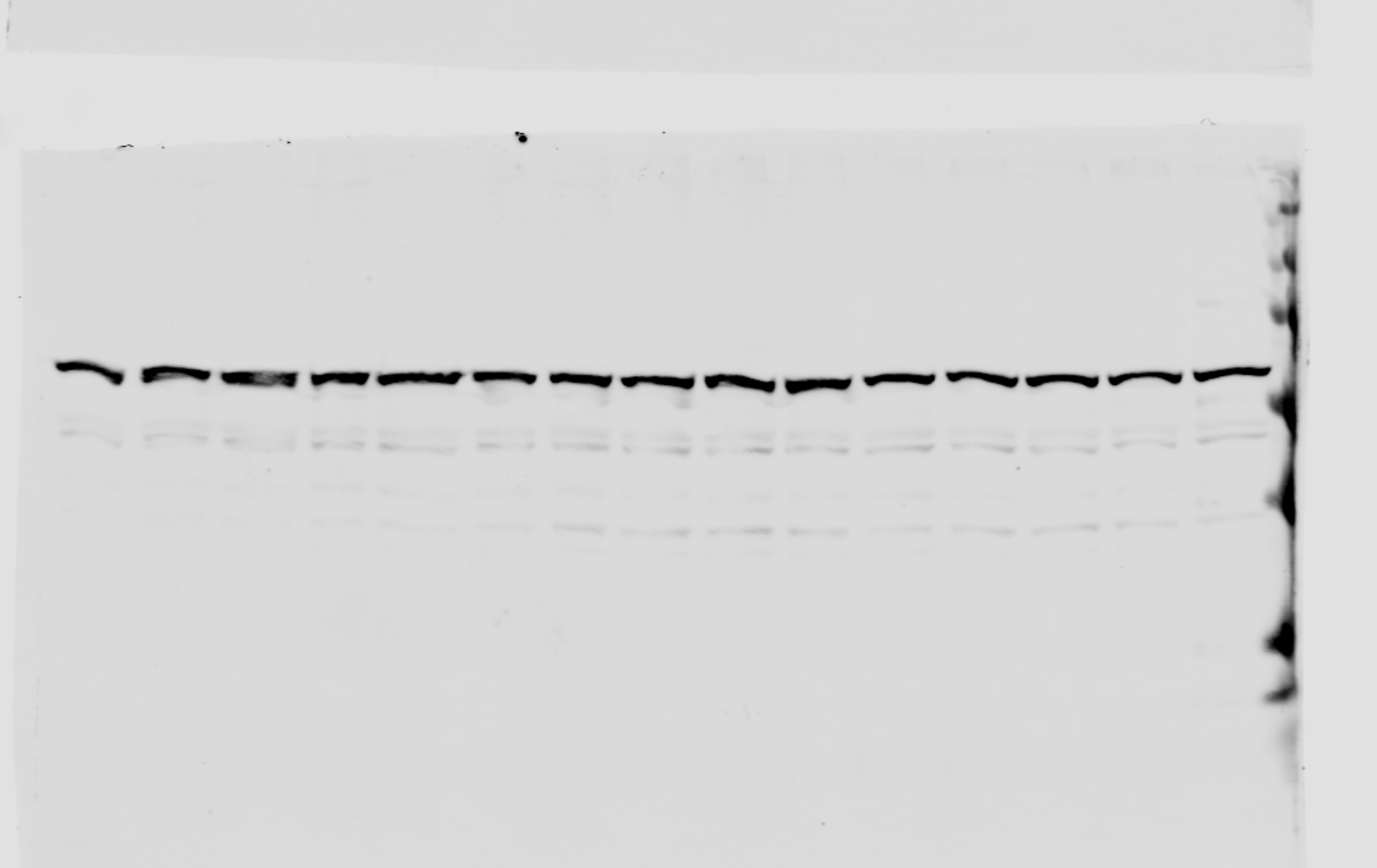

Supplement: Source data 1. [file elife-77424-data1.zip › Source data/Figure 2/Figure 2A_G6PDH.tif]

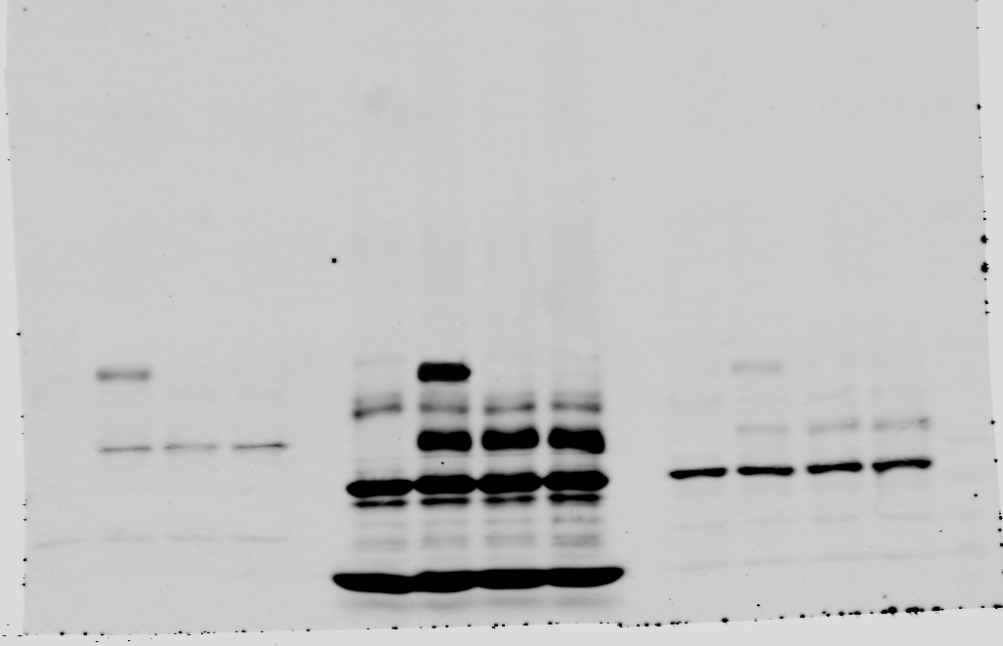

Supplement: Source data 1. [file elife-77424-data1.zip › Source data/Figure 5/figure 5A-FLAG.tif]

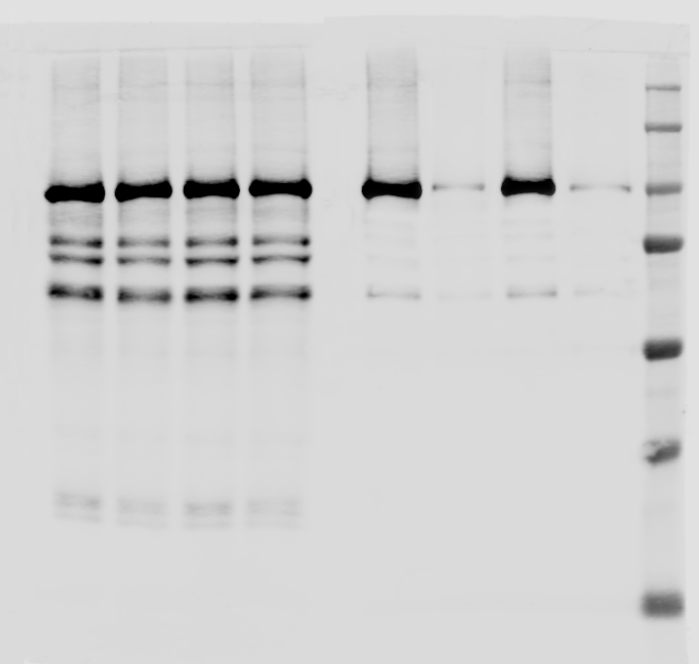

Supplement: Source data 1. [file elife-77424-data1.zip › Source data/Figure 5/figure 5B-Rsp5.tif]

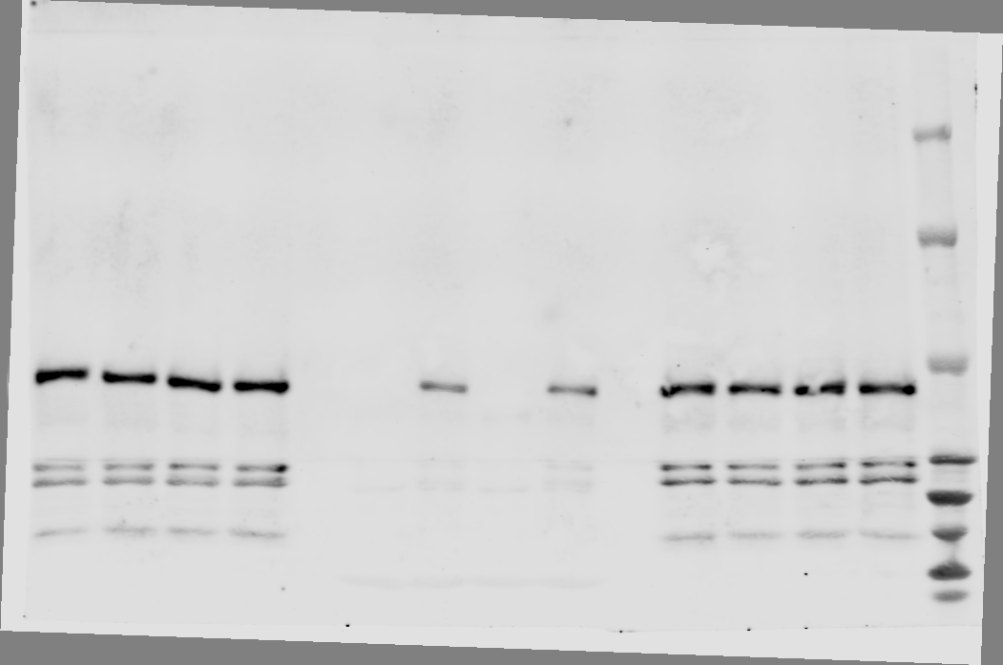

Supplement: Source data 1. [file elife-77424-data1.zip › Source data/Figure 5/Figure 5A-Rsp5.tif]

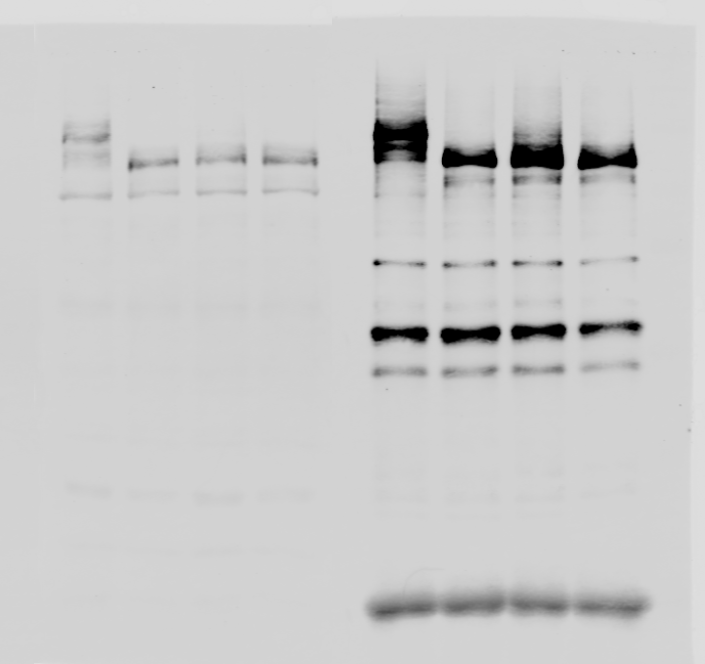

Supplement: Source data 1. [file elife-77424-data1.zip › Source data/Figure 5/figure 5B-FLAG.tif]
