# Supplementary material for: Adaptor linked K63 di-ubiquitin activates Nedd4/Rsp5 E3 ligase
Source: eLife. 2022 Jun 30;11:e77424. doi: 10.7554/eLife.77424 (PMC9282857; doi:10.7554/eLife.77424)
Supplement: Figure 2—figure supplement 1—source data 1. [file elife-77424-fig2-figsupp1-data1.pdf]

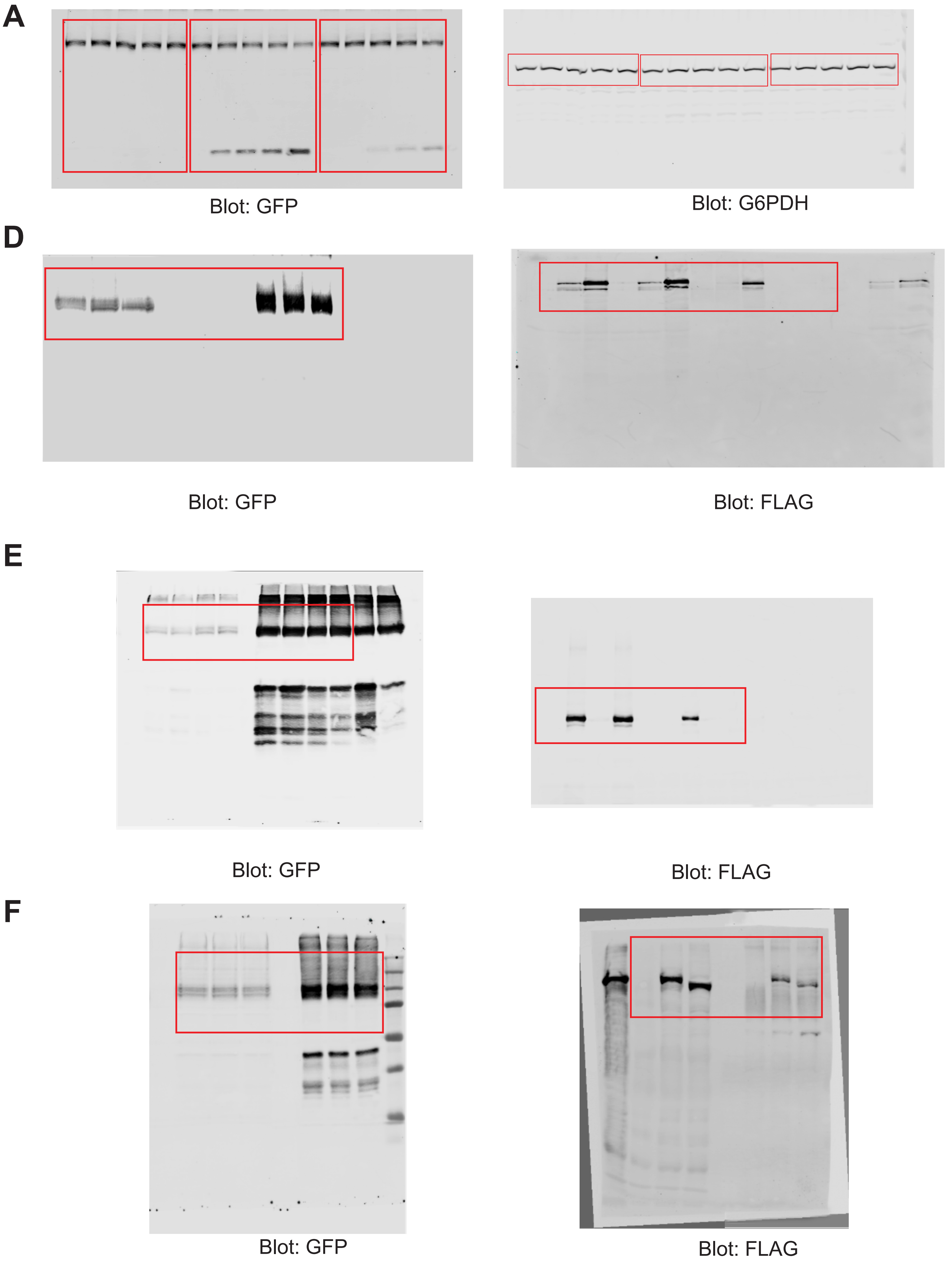

Figure 2-figure supplement 1
