# Supplementary material for: Adaptor linked K63 di-ubiquitin activates Nedd4/Rsp5 E3 ligase
Source: eLife. 2022 Jun 30;11:e77424. doi: 10.7554/eLife.77424 (PMC9282857; doi:10.7554/eLife.77424)
Supplement: Supplementary file 1. [file elife-77424-supp1.docx]

Supplemental file 1: Supplementary Experimental Materials

Table 1: Yeast strains used in this study

| **Strains** | **Genotype** | **Use** | **Source** |
| --- | --- | --- | --- |
| SEY6210 | *MATα, leu2-3,112 ura3-52 his3-Δ200 trp1-Δ901 lys2-801 suc2-Δ9* | Throughout | (Robinson et al., 1988) |
| SEY6210.1 | *MATa, leu2-3,112 ura3-52 his3-Δ200 trp1-Δ901 lys2-801 suc2-Δ9* | Throughout | (Robinson et al., 1988) |
| SUB280 | *MATa, lys2-801 leu2-3,112 ura3-52 his3-D200 trp1-1[am]ubi1-D1::TRP1 ubi2-D2::ura3 ubi3-Dub-2 ubi4-D2::LEU2[pCUP1-Ub-WT::LYS2] [pUB100]* | Figure 1C,  Figure 1-figure supplemental 1C, 2B. | (Spence et al., 1995) |
| SUB413 | *MATa, lys2-801 leu2-3,112 ura3-52 his3-D200 trp1-1[am]ubi1-D1::TRP1 ubi2-D2::ura3 ubi3-Dub-2 ubi4-D2::LEU2[pCUP1-Ub-K63R::LYS2] [pUB100]* | Figure 1C,  Figure 1-figure supplemental 1C, 2B. | (Spence et al., 1995) |
| YMB1005 | *SEY6210, MATα, art1∆::KAN, URA3::pRS306-ART1-HTF* | Figure 1-figure supplemental 3A; Figure 2-figure supplemental 2B. | (Baile et al., 2019) |
| LZY2346 | *SEY6210, MATα, art5∆::NAT, ITR1-GFP::TRP, VPH1-mCherry::KAN* | Figure 2A, 2B, 2C; Figure 2-figure supplemental 3A-3D. | This study |
| LZY2364 | *SEY6210, MATα, art5∆::NAT, ITR1-GFP::TRP, pep4∆::KAN, doa4∆::LEU* | Figure 2D. | This study |
| LZY2348 | *SEY6210, MATα, art5∆::NAT, ITR1-GFP::TRP, pep4∆::KAN* | Figure 2E. | This study |
| YMB1026 | *SEY6210, MATα, art1∆::KAN, MUP1-GFP::HPH, VPH1-mCherry::TRP1* | Figure 2-figure supplemental 1A, 1B, 1C. | Lab stock |
| LZY1544 | *SEY6210.1, MATa, pep4∆::LEU, prb1∆::LEU, art1∆::KAN* | Figure 2-figure supplemental 1D, 1F; Figure 5B. | This study |
| LZY1551 | *SEY6210.1, MATa, pep4∆::LEU, prb1∆::LEU, art1∆::KAN, MUP1-GFP::TRP* | Figure 2-figure supplemental 1D, 1F. | This study |
| LZY1869 | *SEY6210.1, MATa, pep4∆::LEU, prb1∆::LEU, art1∆::KAN, mup1∆::TRP* | Figure 2-figure supplemental 1E. | This study |
| JMY527 | *SEY6210, MATα, npr1∆::HISMX6* | Figure 2-figure supplemental 2A. | (MacGurn et al., 2011) |
| YMB1436 | *SEY6210, MATα, art1∆::KAN, URA3::pRS306-art1K486R-HTF* | Figure 2-figure supplemental 2B. | (Baile et al., 2019) |
| HHY588 | *SEY6210.1, MATa, art1∆::KAN* | Figure 2-figure supplemental 4A, 4B. | (Ho et al., 2017) |
| LZY1196 | *SEY6210, MATα, SEC7-mCherry::HYG* | Figure 3A. | This study |
| HHY867 | *SEY6210, MATα, art5∆::NAT* | Figure 3B, 3C. | Lab stock |
| LZY2405 | *SEY6210, MATα, rsp5∆::HIS, LEU2::pRS305-GFP-linker-RSP5, art5∆::HYG* | Figure 3D­, 3E. | This study |
| CLY364.1 | *BY4742, MATα, ART5-GFP::HIS* | Figure 3F, 3G. | Lab stock |
| LZY1576 | *BY4742, MATα, ART5-GFP::HIS, itr1∆::HYG* | Figure 3F, 3G. | This study |
| YEG280 | *SEY6210, MATα, LEU2::pRS305-SEC7-mCherry, art1∆::KAN, URA3:: pRS306-ART1-mNeonGreen* | Figure 3-figure supplemental 1A, 1D. | (Baile et al., 2019) |
| YMB1796 | *SEY6210, MATα, LEU2::pRS305-SEC7-mCherry, art1∆::KAN, URA3:: pRS306-art1-K486R-mNeonGreen* | Figure 3-figure supplemental 1B, 1D. | Lab stock |
| YMB1798 | *SEY6210, MATα, LEU2::pRS305-SEC7-mCherry,, art1∆::KAN, URA3:: pRS306-art1-∆PY-mNeonGreen* | Figure 3-figure supplemental 1C, 1D. | Lab stock |
| YMB1438 | *SEY6210, MATα, LEU2::pRS305-GFP-linker-RSP5, art1∆::KAN* | Figure 3-figure supplemental 1E, 1F. | Lab stock |
| YMB1362 | *SEY6210, MATα, LEU2::pRS305-GFP-linker-RSP5* | Figure 3-figure supplemental 1G, 1H. | Lab stock |
| YMB1782 | *SEY6210, MATα, art1∆::KAN, URA3:: pRS306-ART1-mNeonGreen, mup1∆::TRP, LEU2::pRS305* | Figure 3-figure supplemental 2A, 2B. | Lab stock |
| YMB1784 | *SEY6210, MATα, art1∆::KAN, URA3:: pRS306-ART1-mNeonGreen, mup1∆::TRP, LEU2::pRS305-MUP1-HTF* | Figure 3-figure supplemental 2A, 2B. | Lab stock |
| YMB1786 | *SEY6210, MATα, art1∆::KAN, URA3:: pRS306-ART1-mNeonGreen, mup1∆::TRP, LEU2::pRS305-mup1-Q49R-HTF* | Figure 3-figure supplemental 2A, 2B. | Lab stock |
| GOY24 | *SEY6210.1, MATa, pep4∆::LEU, prb1∆::LEU* | Figure 5A. | Lab stock |
| LZY1591 | *SEY6210, MATα, art1∆::KAN, rsp5Δ::NAT, LYS2::pRS307-ART1-HTF, pRS414-RSP5* | Figure 6A; Figure 6-figure supplemental 1A, 1D. | This study |
| LZY1594 | *SEY6210, MATα, art1∆::KAN, rsp5Δ::NAT, LYS2::pRS307-ART1-HTF, pRS414-rsp5-Y516A* | Figure 6A; Figure 6-figure supplemental 1A. | This study |
| LZY1595 | *SEY6210, MATα, art1∆::KAN, rsp5Δ::NAT, LYS2::pRS307-ART1-HTF, pRS414-rsp5-F618A* | Figure 6A, 6B, 6C; Figure 6-figure supplemental 1A, 1D. | This study |
| LZY2328 | *SEY6210, MATα, art1∆::KAN, rsp5Δ::NAT, LYS2::pRS307-ART1-HTF, pRS414-RSP5, ubp2∆::HYG* | Figure 6A; Figure 6-figure supplemental 1D, 1E. | This study |
| LZY2329 | *SEY6210, MATα, art1∆::KAN, rsp5Δ::NAT, LYS2::pRS307-ART1-HTF, pRS414-rsp5-F618A, ubp2∆::HYG* | Figure 6A; Figure 6-figure supplemental 1D, 1E. | This study |
| LZY2428 | *MATa, lys2-801 leu2-3,112 ura3-52 his3-D200 trp1-1[am]ubi1-D1::TRP1 ubi2-D2::ura3 ubi3-Dub-2 ubi4-D2::LEU2[pCUP1-Ub-K63R::LYS2] [pUB100], ubp2∆::HYG* | Figure 6D. | This study |
| HHY504 | *SEY6210.1, MATa, ART1-HTF::TRP* | Figure 6-figure supplemental 1B, 1C. | (Ho et al., 2017) |
| HHY514 | *SEY6210, MATα, ubp2∆::KAN* | Figure 6-figure supplemental 2A, 2B. | (Ho et al., 2017) |

Table 2: Plasmids used in this study

| **Name** | **Description** | **Use** | **Source** |
| --- | --- | --- | --- |
| pLZ1831 | pRS416-Art5-HTF | Figure 1B, 1C, 2A, 2D, 3D, 5A; Figure 2-figure supplemental 3A, 3B. | This study |
| pLZ1922 | pRS416-art5-K364R-HTF | Figure 1B, 1C, 2A, 2D, 3D, 5A; Figure 2-figure supplemental 3A, 3B. | This study |
| pLZ1943 | pRS416-art5-∆PY-HTF (P532A, Y535A, P560A, Y562A, P580A, Y582A) | Figure 1B, 1C, 2A, 3D, 5A; Figure 2-figure supplemental 3C, 3D. | This study |
| pLZ2005 | pRS416-Art5-GFP | Figure 2B, 2C. | This study |
| pLZ2044 | pRS416-art5-K364R-GFP | Figure 2B, 2C. | This study |
| pLZ2045 | pRS416-art5-∆PY-GFP (P532A, Y535A, P560A, Y562A, P580A, Y582A) | Figure 2B, 2C. | This study |
| pLZ555 | pRS426-pCUP1-Myc-Ub | Figure 2D, 6C | This study |
| pHH63 | pRS415-Art5-3HA | Figure 2E, 6A, 6B, 6C. | Lab stock |
| pLZ2035 | pRS415-art5-K364R-3HA | Figure 2E, 6B, 6D. | This study |
| pLZ2055 | pRS416-art5-K364R-HTF-2xUb | Figure 2-figure supplemental 3A, 3B. | This study |
| pLZ1955 | pRS416-art5-∆PY-HTF-1xUb | Figure 1B, 1C. | This study |
| pLZ1956 | pRS416-art5-∆PY-HTF-2xUb | Figure 1B, 1C, Figure 2-figure supplemental 3C, 3D. | This study |
| pLZ1957 | pRS416-art5-∆PY-HTF-3xUb | Figure 1B, 1C. | This study |
| pLZ1960 | pRS416-art5-∆PY-HTF-4xUb | Figure 1B, 1C. | This study |
| pMB198 | pRS306-Art1-HTF (WT) | Figure 1-figure supplemental 1B, 1C; Figure 5B; Figure 2-figure supplemental 2A; Figure 2-figure supplemental 4A, 4B; Figure 3-figure supplemental 1E, 1F. | (Baile et al., 2019) |
| pMB355 | pRS306-art1-K486R-HTF | Figure 1-figure supplemental 1B, 1C; Figure 5B; Figure 2-figure supplemental 2A; Figure 2-figure supplemental 4A, 4B; Figure 3-figure supplemental 1E, 1F. | (Baile et al., 2019) |
| pMB322 | pRS306-art1-∆PY-HTF (P678A, Y681A, P690A, Y692A) | Figure 1-figure supplemental 1B, 1C; Figure 5B; Figure 2-figure supplemental 4A, 4B; Figure 3-figure supplemental 1E, 1F. | (Baile et al., 2019) |
| pLZ1849 | pRS306-ART1-HTF-1xUb | Figure 2-figure supplemental 4A. | This study |
| pLZ1850 | pRS306-ART1-HTF-2xUb | Figure 2-figure supplemental 4A. | This study |
| pLZ1851 | pRS306-ART1-HTF-3xUb | Figure 2-figure supplemental 4A. | This study |
| pLZ1852 | pRS306-art1-K486R-HTF-1xUb | Figure 2-figure supplemental 4A. | This study |
| pLZ1853 | pRS306-art1-K486R-HTF-2xUb | Figure 2-figure supplemental 4A. | This study |
| pLZ1854 | pRS306-art1-K486R-HTF-3xUb | Figure 2-figure supplemental 4A. | This study |
| pLZ1835 | pRS306-art1-∆PY-HTF-1xUb | Figure 1-figure supplemental 1B, 1C; Figure 2-figure supplemental 4B. | This study |
| pLZ1841 | pRS306-art1-∆PY-HTF-2xUb | Figure 1-figure supplemental 1B, 1C; Figure 2-figure supplemental 4B; Figure 6-figure supplemental 1A. | This study |
| pLZ1836 | pRS306-art1-∆PY-HTF-3xUb | Figure 1-figure supplemental 1B, 1C; Figure 2-figure supplemental 4B; Figure 6-figure supplemental 1A. | This study |
| pHH139 | pRS416-Art4-HTF | Figure 1-figure supplemental 2B. | Lab stock |
| pHH168 | pRS416-art4-HTF (K235,245,264,267R) | Figure 1-figure supplemental 2B. | Lab stock |
| pHH153 | pRS416-art4-∆PY-HTF (P415A, Y417A, P488A, Y490A, P640A, Y642A, P657A, Y659A) | Figure 1-figure supplemental 2B. | Lab stock |
| pLZ1866 | pRS416-art4-∆PY-HTF-1xUb | Figure 1-figure supplemental 2B. | This study |
| pLZ1867 | pRS416-art4-∆PY-HTF-2xUb | Figure 1-figure supplemental 2B. | This study |
| pLZ1868 | pRS416-art4-∆PY-HTF-3xUb | Figure 1-figure supplemental 2B. | This study |
| pTK138 | pCM189-Art1-HTF (TetOff regulated Art1) | Figure 2-figure supplemental 1A, Figure 6-figure supplemental 2C. | (Guiney et al., 2016) |
| pLZ2034 | pCM189-art1-K486R-HTF (TetOff regulated Art1) | Figure 2-figure supplemental 1A. | This study |
| pMB299 | pRS416-p(GPD)-ART1-mNG | Figure 2-figure supplemental 1D. | Lab stock |
| pLZ211 | pRS416-p(GPD)-ART1-HTF (WT) | Figure 2-figure supplemental 1D, Figure 3-figure supplemental 1G, 1H. | Lab stock |
| pEG149 | pRS305-Mup1-GFP (WT) | Figure 2-figure supplemental 1E. | (Guiney et al., 2016) |
| pEG150 | pRS305-mup1-Q49R-GFP | Figure 2-figure supplemental 1E. | (Guiney et al., 2016) |
| pMB438 | pRS416-p(GPD)-art1-K486R-HTF | Figure 2-figure supplemental 1F, Figure 3-figure supplemental 1G, 1H. | Lab stock |
| pMB437 | pRS416-p(GPD)-art1-∆PY-HTF | Figure 3-figure supplemental 1G, 1H. |  |
| pGEX-6p-1 | Empty vector for GST fusion protein expression | Figure 4A. | Lab stock |
| pLZ1968 | pGEX-6p-1-HECT (WT) | Figure 4A. | This study |
| pLZ1977 | pGEX-6p-1-HECT (Y516A) | Figure 4A. | This study |
| pLZ1978 | pGEX-6p-1-HECT (F618A) | Figure 4A. | This study |
| pLZ1979 | pGEX-6p-1-HECT (Y516A/F618A) | Figure 4A. | This study |
| pLZ1926 | pET28b-Mms2 | Figure 1-figure supplemental 3B; Figure 4A, 4C, 4G; Figure 4-figure supplemental 1C, 1F; Figure 5F, 5G. | This study |
| pLZ1931 | pGEX-6p-1-Ubc13 | Figure 1-figure supplemental 3B; Figure 4A, 4C, 4G; Figure 4-figure supplemental 1C, 1F; Figure 5F, 5G. | This study |
| pLZ1971 | pET21a-Ub (WT) | Figure 4A, 4B. | This study |
| pLZ1970 | pET21a-Ub (D77), proximal end Ub, for K48 or K63 linked diUb synthesis | Figure 1-figure supplemental 3B; Figure 4C, 4D, 4F, 4G; Figure 4-figure supplemental 1C, 1E, 1F; Figure 5F, 5G. | This study |
| pLZ1972 | pET21a-Ub (K63R), for distal end Ub conjugation of K63 linked diUb synthesis | Figure 1-figure supplemental 3B; Figure 4C. | This study |
| pLZ1913 | pGEX-6p-1-2xUb | Figure 4D, 4E. | This study |
| pLZ1912 | pGEX-6p-1-1xUb | Figure 4E. | This study |
| pLZ1914 | pGEX-6p-1-3xUb | Figure 4E. | This study |
| YM83 | pGEX-6p-1-E2-25K, for K48 linked diUb synthesis | Figure 4F. | Lab stock |
| pLZ2036 | pET21a-Ub (K48R), for K48 linked diUb synthesis | Figure 4F; Figure 4-figure supplemental 1E. | This study |
| pLZ2021 | pET21a-Ub (K63R/I44A), for K63 linked Ub^WT^-Ub^I44A^ synthesis | Figure 4G. | This study |
|  |  |  |  |
| pLZ2006 | pET28a-6xHIS-sumo-HECT (383-809 of Rsp5) | Figure 4B, 4C, 4D, 4F, 4G. | This study |
| pLZ2056 | pET21a-Ub (I44A) | Figure 4-figure supplemental 1A. | This study |
| pLZ2012 | pET28a-6xHIS-sumo-HECT (383-809 of Rsp5, F618A mutant) | Figure 4-figure supplemental 1B, 1C, 1D, 1E, 1F. | This study |
| pLZ2024 | pGEX-6p-Art5-PY-motif (521-586 of Art5) | Figure 5C. | This study |
| pLZ2025 | pGEX-6p-Art5-PY-motif (521-586 of Art5, P532A, Y535A, P560A, Y562A, P580A, Y582A) | Figure 5C. | This study |
| pLZ2022 | pGEX-6p-Art1-PY-motif (661-710 of Art1) | Figure 5D. | This study |
| pLZ2023 | pGEX-6p-Art1-PY-motif (661-710 of Art1, P678A, Y681A, P690A, Y692A) | Figure 5D. | This study |
| pLZ1898 | pET28a-6xHIS-sumo-WW1-HECT (217-809 of Rsp5) | Figure 5C, 5D. | This study |
| YM1320 | pET28a-6xHis-Sumo-Any1(17-361) | Figure 5E, 5F, 5G; Figure 5-figure supplemental 2A | This study |
| YM84 | pGEX-6p-1-YUH1, to deblock the proximal Ub-D77 of K63 linked diUb | Figure 5F, 5G | Lab stock |
| YM1920 | pET28a-6xHis-sumo-Pub1 (WW2-C of Pub1) | Figure 5E, 5F; Figure 5-figure supplemental 2B, 2C | This study |
| YM1921 | pET28a-6xHis-sumo-Pub1 (WW2-C of Pub1, F576A mutant) | Figure 5G | This study |
| pMB176 | pRS414-Rsp5 | Figure 6A, Figure 6-figure supplemental 1A, 1D, 1E. | Lab stock |
| pMB417 | pRS414-rsp5-Y516A | Figure 6-figure supplemental 1A. | Lab stock |
| pMB430 | pRS414-rsp5-F618A | Figure 6A, Figure 6-figure supplemental 1A, 1D, 1E. | Lab stock |
| pHH304 | pRS426-pCUP1-Myc-Ub (K63R) | Figure 6C | Lab stock |
| pHH303 | pRS426-pCUP1-Myc-Ub (K48R) | Figure 6C | Lab stock |
| pRS425 | Empty vector as a control. | Figure 6-figure supplemental 1B. | Lab stock |
| pLZ151 | pRS425-Ubp1 | Figure 6-figure supplemental 1B. | This study |
| pLZ153 | pRS425-Ubp2 | Figure 6-figure supplemental 1B. | This study |
| pLZ154 | pRS425-Ubp3 | Figure 6-figure supplemental 1B. | This study |
| pLZ156 | pRS425-Doa4 | Figure 6-figure supplemental 1B. | This study |
| pLZ158 | pRS425-Ubp5 | Figure 6-figure supplemental 1B. | This study |
| pLZ160 | pRS425-Ubp6 | Figure 6-figure supplemental 1B. | This study |
| pLZ161 | pRS425-Ubp7 | Figure 6-figure supplemental 1B. | This study |
| pLZ162 | pRS425-Ubp9 | Figure 6-figure supplemental 1B. | This study |
| pLZ163 | pRS425-Ubp11 | Figure 6-figure supplemental 1B. | This study |
| pLZ165 | pRS425-Ubp12 | Figure 6-figure supplemental 1B. | This study |
| pLZ168 | pRS425-Ubp13 | Figure 6-figure supplemental 1B. | This study |
| pLZ169 | pRS425-Ubp15 | Figure 6-figure supplemental 1B. | This study |
| pHH27 | pSR56-Ubp2 (WT) | Figure 6-figure supplemental 1C, 1E. | Lab stock |
| pHH28 | pSR56-ubp2 (C745V) | Figure 6-figure supplemental 1C, 1E. | Lab stock |
| pLZ2050 | pCM189-Art5-HTF (TetOff regulated Art5) | Figure 6-figure supplemental 2A. | This study |
|  |  |  |  |

Supplemental References:

Baile, M.G., E.L. Guiney, E.J. Sanford, J.A. MacGurn, M.B. Smolka, and S.D. Emr. 2019. Activity of a ubiquitin ligase adaptor is regulated by disordered insertions in its arrestin domain. *Mol Biol Cell*. 30:3057-3072.

Guiney, E.L., T. Klecker, and S.D. Emr. 2016. Identification of the endocytic sorting signal recognized by the Art1-Rsp5 ubiquitin ligase complex. *Mol Biol Cell*. 27:4043-4054.

Ho, H.C., J.A. MacGurn, and S.D. Emr. 2017. Deubiquitinating enzymes Ubp2 and Ubp15 regulate endocytosis by limiting ubiquitination and degradation of ARTs. *Mol Biol Cell*. 28:1271-1283.

MacGurn, J.A., P.C. Hsu, M.B. Smolka, and S.D. Emr. 2011. TORC1 regulates endocytosis via Npr1-mediated phosphoinhibition of a ubiquitin ligase adaptor. *Cell*. 147:1104-1117.

Robinson, J.S., D.J. Klionsky, L.M. Banta, and S.D. Emr. 1988. Protein sorting in Saccharomyces cerevisiae: isolation of mutants defective in the delivery and processing of multiple vacuolar hydrolases. *Mol Cell Biol*. 8:4936-4948.

Spence, J., S. Sadis, A.L. Haas, and D. Finley. 1995. A ubiquitin mutant with specific defects in DNA repair and multiubiquitination. *Mol Cell Biol*. 15:1265-1273.
