# Supplementary material for: Adaptor linked K63 di-ubiquitin activates Nedd4/Rsp5 E3 ligase
Source: eLife. 2022 Jun 30;11:e77424. doi: 10.7554/eLife.77424 (PMC9282857; doi:10.7554/eLife.77424)

Purified proteins for ITC assays

(Protein with arrow labeled were selected anad pooled together for ITC analyis):

1. Mono-Ub variants purification:

The lysate for Ub (WT or mutants) was heated at 70°C for 5 min then the supernatant was loaded onto SP Sepharose Fast Flow resin. The Ub was eluted using a linear gradient of 0-500mM NaCl. The eluted Ub mutants were fractionated by Superdex 200-exclusion column.

(A) Ub-WT mutant, S200, gel filtration

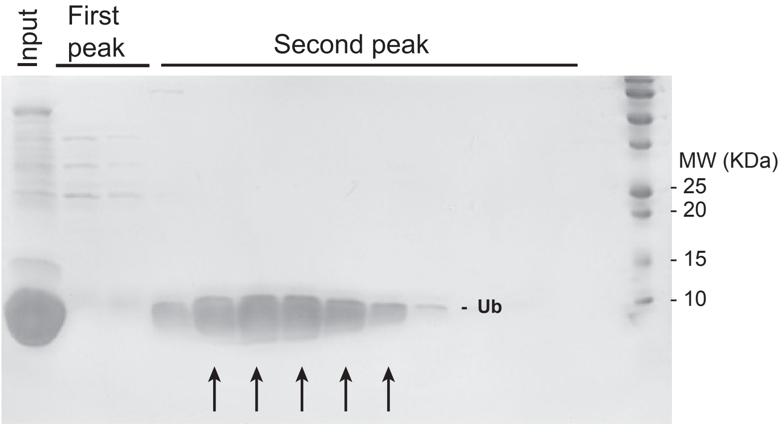

(B) Ub-D77 mutant, S200, gel filtration

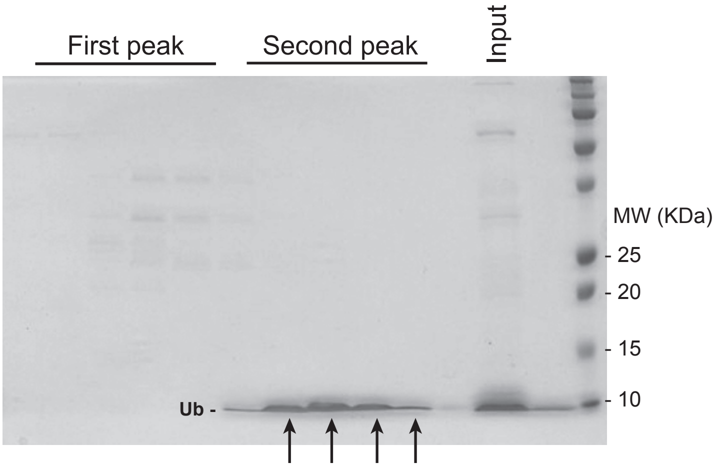

2. K63 linked di-Ub purification:

The K63 di-Ub synthesis mixture was first purified by cation exchange column SP fast flow. Then the eluted proteins by high salt were loaded onto S75 gel filtration.

K63 linked di-Ub, S75, gel filtration

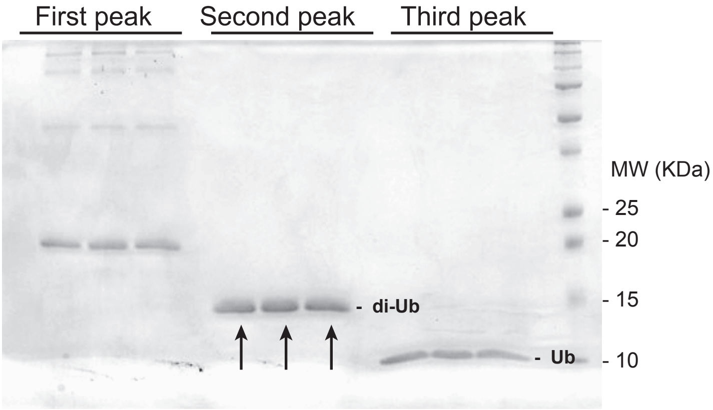

3. M1 linked di-Ub purification:

The Glutathione Sepharose® 4B resin was bound with lysate containing GST-Ub-Ub. The resin was digested by PreScission Protease then the supernantant was loaded onto S200 gel filtration.

M1 linked di-Ub, S200, gel filtration

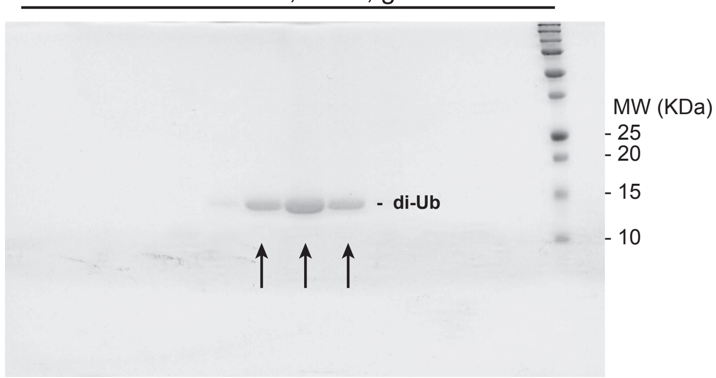

4. K48 linked di-Ub purification:

The K48 di-Ub synthesis mixture was first purified by cation exchange column SP fast flow. Then the eluted proteins by high salt were loaded onto S75 gel filtration.

K48 linked di-Ub, S75, gel filtration

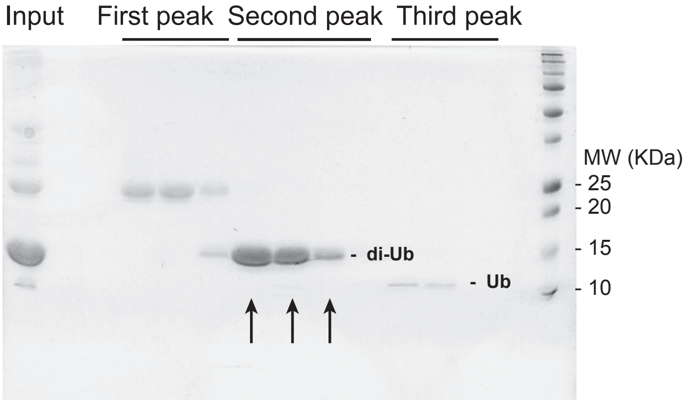

5. K63 linked di-Ub (Ub^{WT}-Ub^{I44A}) purification:

The K63 di-Ub (Ub^{WT}-Ub^{I44A}) synthesis mixture was first purified by cation exchange column SP fast flow. Then the eluted proteins by high salt were loaded onto S75 gel filtration.

K63 linked di-Ub, S75, gel filtration

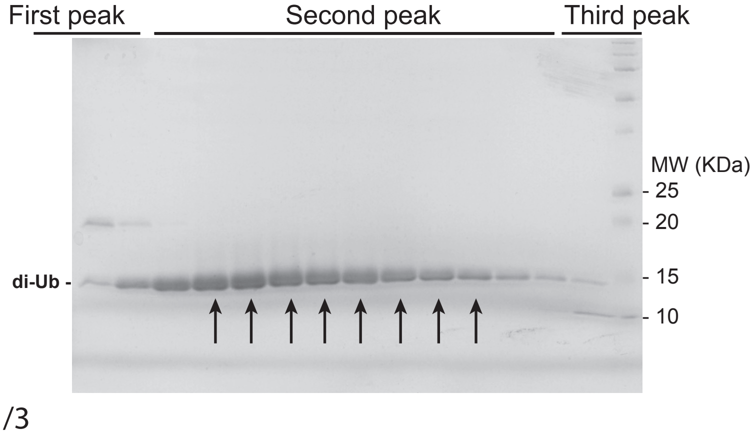

6. Rsp5 HECT domain purification:

The TALON cobalt resin was bound with lysate containing 6HIS-SUMO-HECT. The resin was digested by Ulp1 enzyme then the supernantant was loaded onto S200 gel filtration.

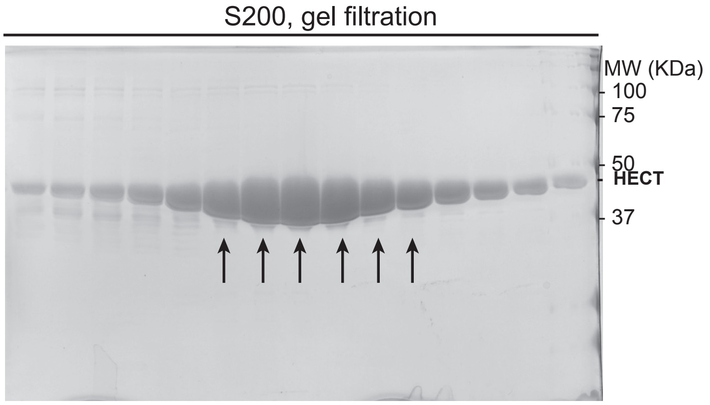

7. Rsp5 ww1-HECT domain purification:

The TALON cobalt resin was bound with lysate having 6HIS-SUMO-ww1-HECT. The resin was digested by Ulp1 enzyme then the supernantant was loaded onto S200 gel filtration.

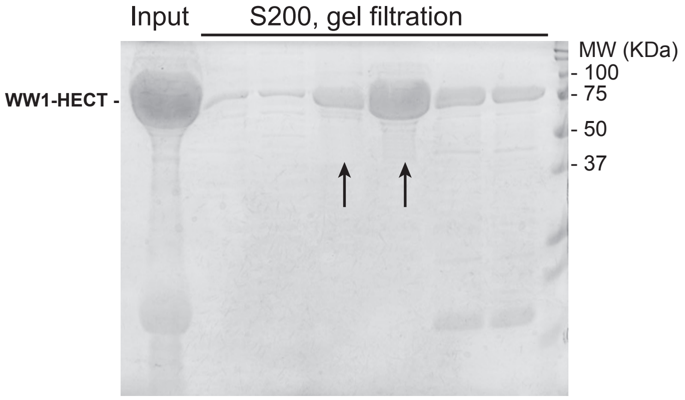

8. PY1-2(Art1) and PY1-2(*art1$^{\Delta PY}$*) purification:

The Glutathione Sepharose® 4B resin was bound with lysate containing GST-PY1-2(Art1) or GST-PY1-2(*art1$^{\Delta PY}$*). The resin was digested by PreScission Protease then the supernantant was loaded onto S200 gel filtration.

(A) PY1-2(Art1), S200, gel filtration

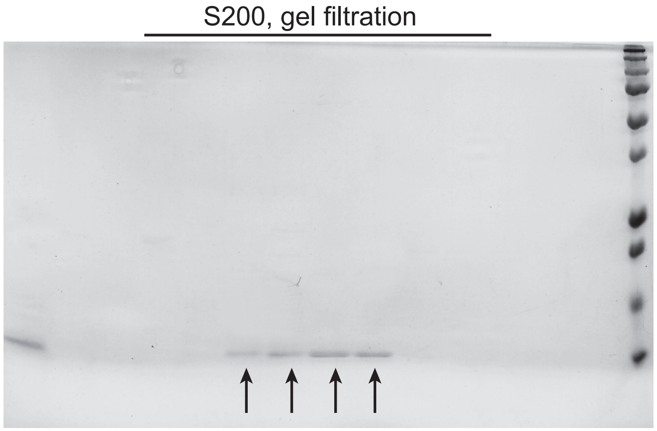

(B) PY1-2(*art1$^{\Delta PY}$*), S200, gel filtration

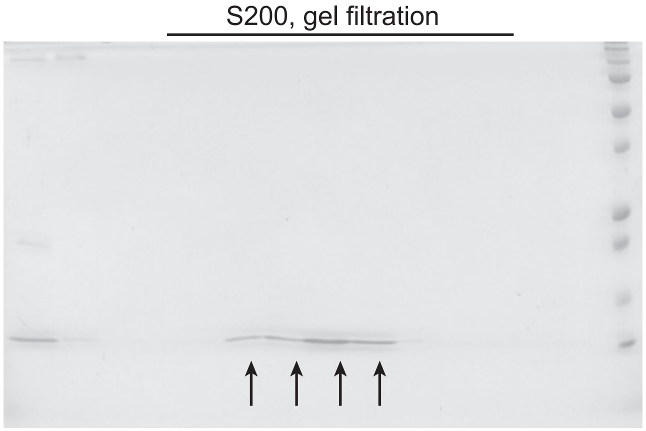

9. PY1-3(Art5) and PY1-3(*art5$^{\Delta PY}$*) purification:

The Glutathione Sepharose® 4B resin was bound with lysate containing GST-PY1-3(Art5) or GST-PY1-3(*art5$^{\Delta PY}$*). The resin was digested by PreScission Protease then the supernantant was loaded onto S200 gel filtration.

(A) PY1-3(Art5), S200, gel filtration

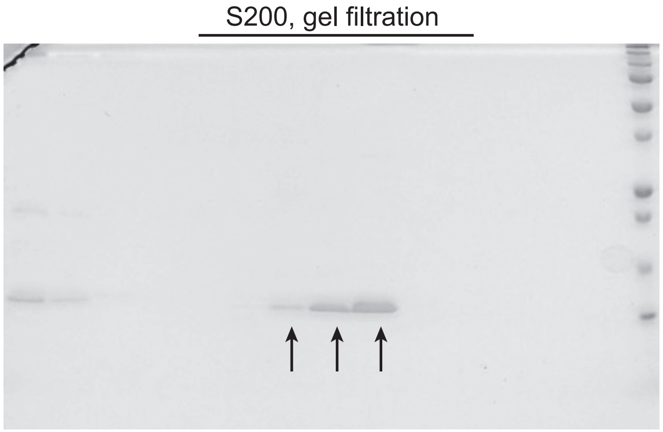

(B) PY1-3(*art5$^{\Delta PY}$*), S200, gel filtration

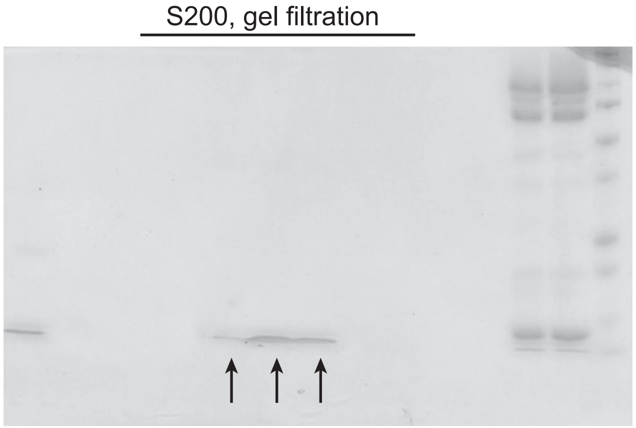

10. Pub1 purification:

The TALON cobalt resin was bound with lysate having 6HIS-SUMO-Pub1. The resin was digested by Ulp1 enzyme then the supernantant was loaded onto S200 gel filtration.

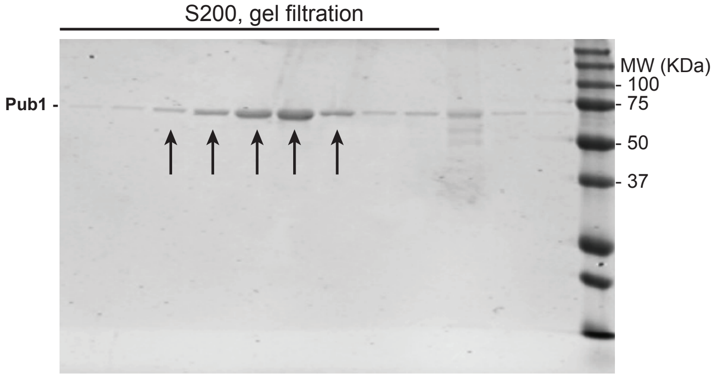

11. Any1 purification:

The TALON cobalt resin was bound with lysate having 6HIS-SUMO-Any1. The resin was digested by Ulp1 enzyme then the supernantant was loaded onto S200 gel filtration.

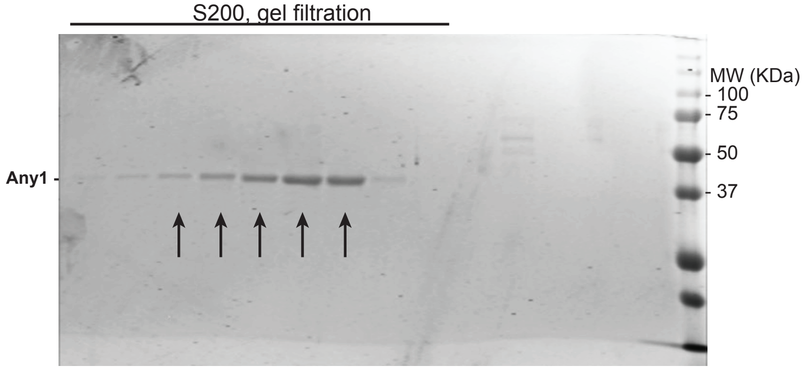

12. Any1-diUb purification.

(A) Synthesis of Any1-diUb

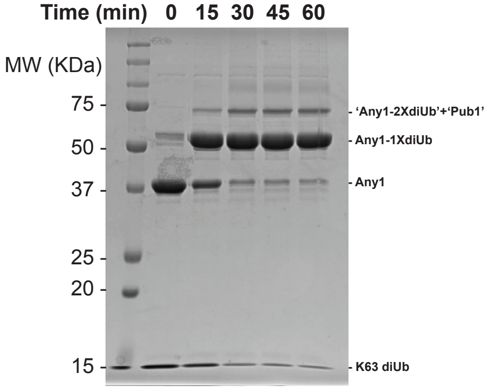

(B) The Any1-diUb synthesis mixture was loaded onto anion exchange to remove E1,E2 and E2. The eluted proteins were further fractionated by S200 gel filtration.

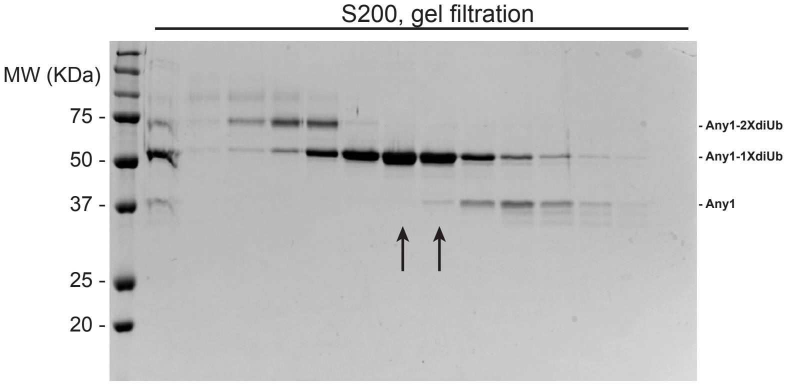

Supplement: Supplementary file 2. [file elife-77424-supp2.pdf]
