# Supplementary material for: Adaptor linked K63 di-ubiquitin activates Nedd4/Rsp5 E3 ligase
Source: eLife. 2022 Jun 30;11:e77424. doi: 10.7554/eLife.77424 (PMC9282857; doi:10.7554/eLife.77424)

1. Mono-Ub variants purification:

(A) Ub-WT mutant, S200, gel filtration

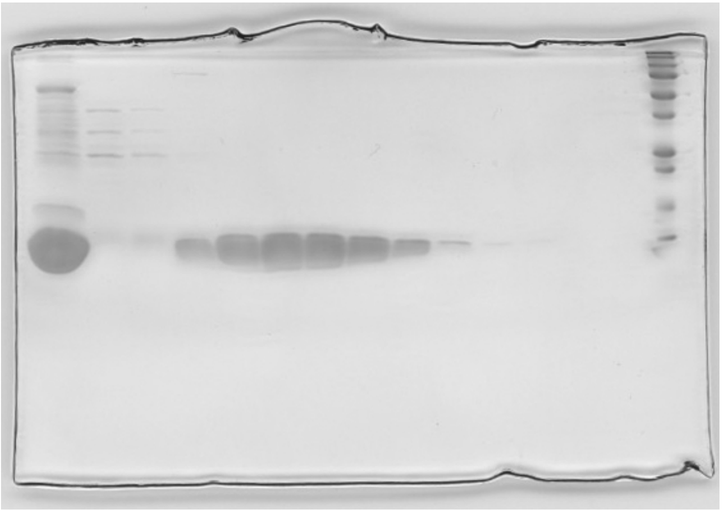

(B) I44A mutant, S200, gel filtration

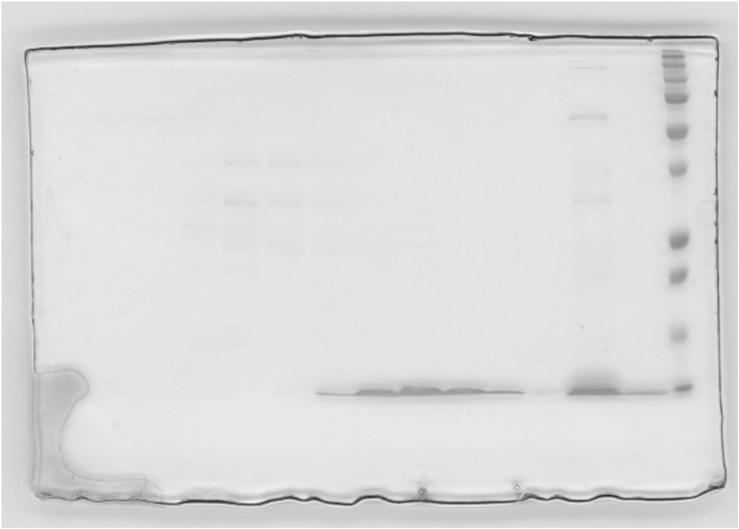

2. K63 linked di-Ub purification:

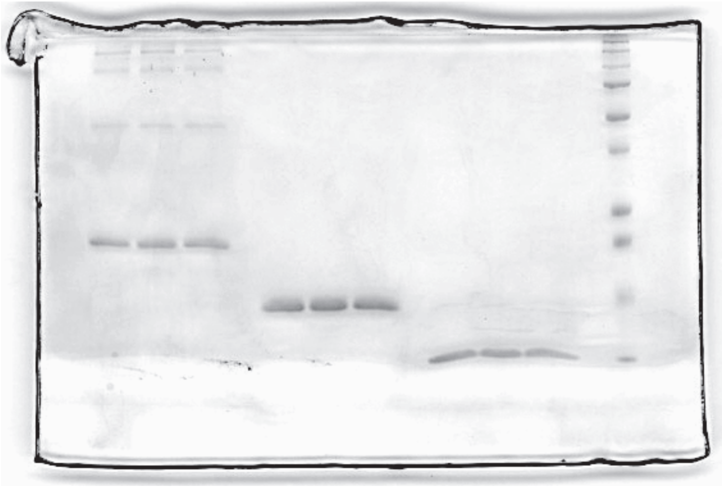

3. M1 linked di-Ub purification:

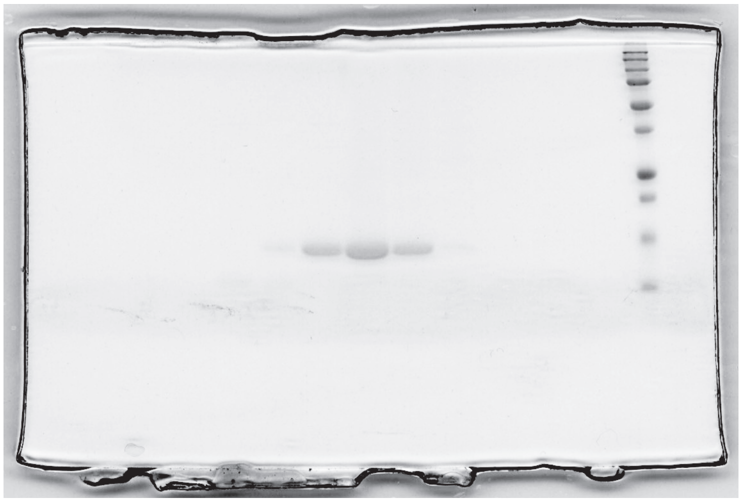

4. K48 linked di-Ub purification:

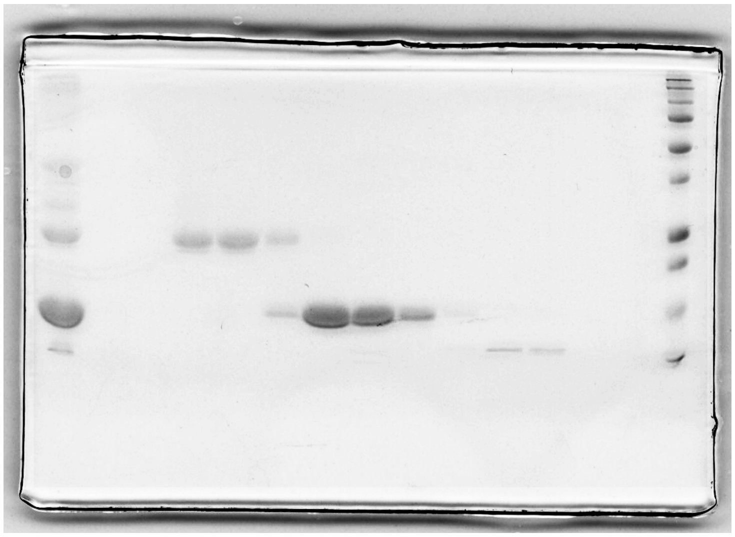

5. K63 linked di-Ub (Ub^{WT}-Ub^{I44A}) purification:

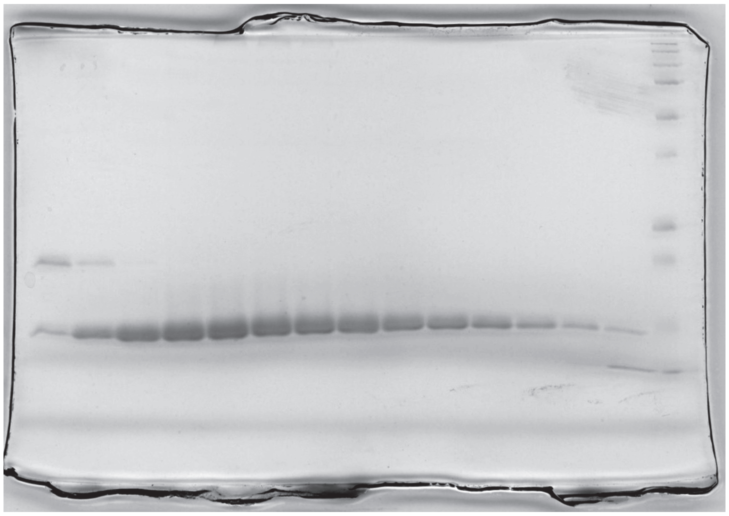

6. Rsp5 HECT domain purification:

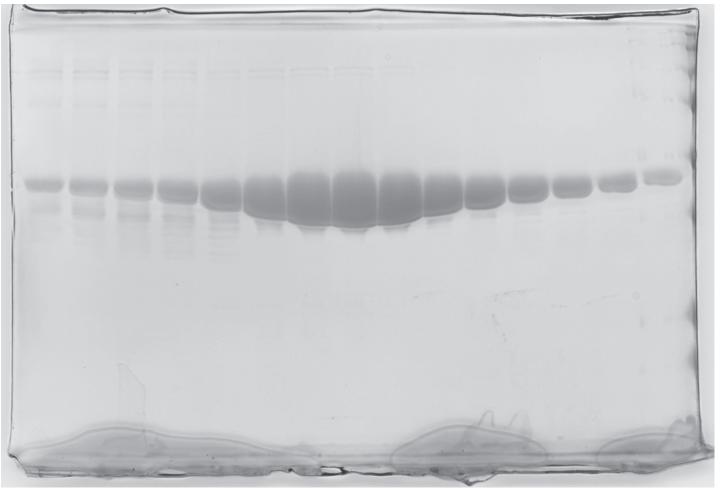

7. Rsp5 ww1-HECT domain purification:

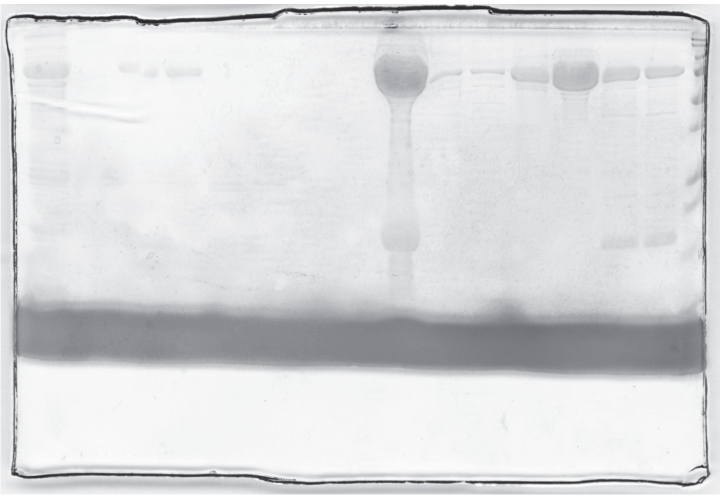

8. PY1-2(Art1) and PY1-2(*art1*$^{\Delta PY}$) purification:

(A) PY1-2(Art1), S200, gel filtration

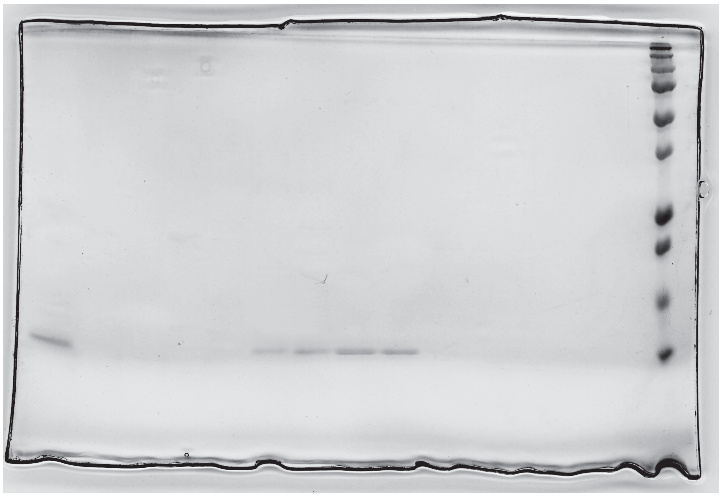

(B) PY1-2(*art1*$^{\Delta PY}$), S200, gel filtration

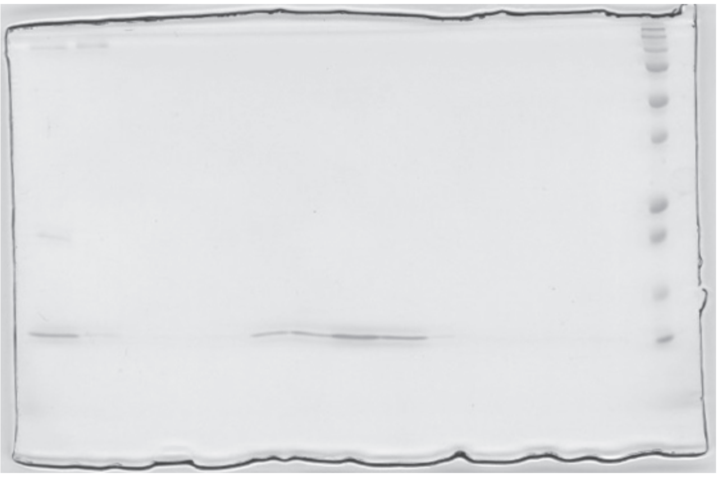

9. PY1-3(Art5) and PY1-3(*art5*$^{\Delta PY}$) purification:

(A) PY1-3(Art5), S200, gel filtration

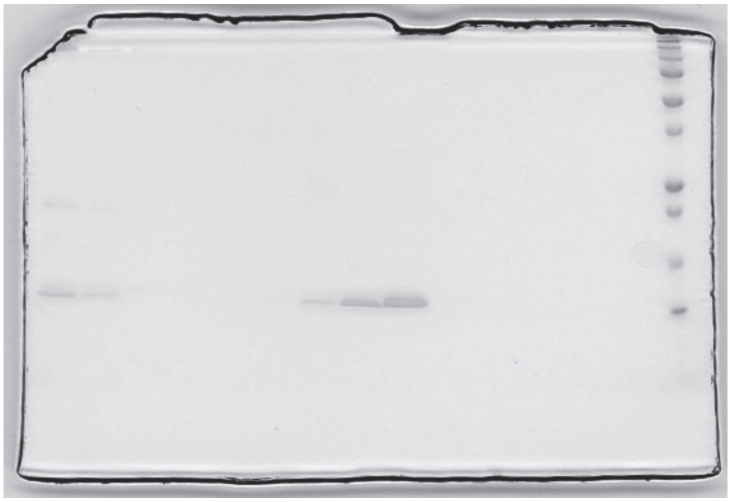

(B) PY1-3(*art5*$^{\Delta PY}$), S200, gel filtration

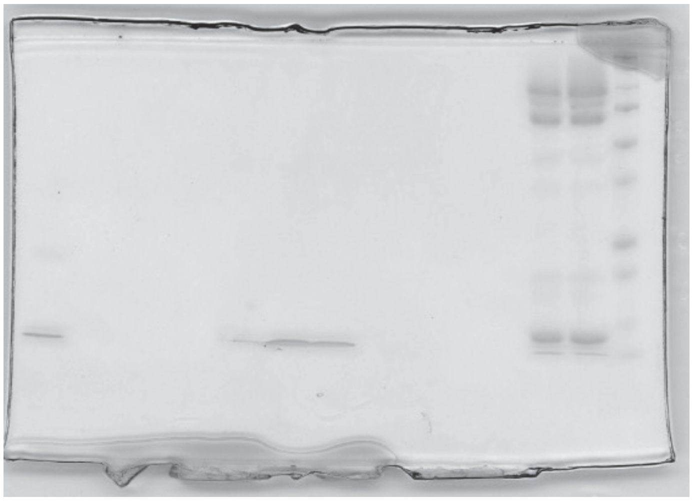

10. Pub1 purification:

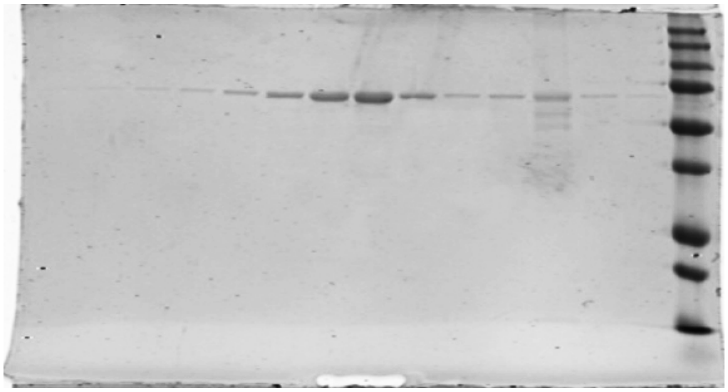

11. Any1 purification:

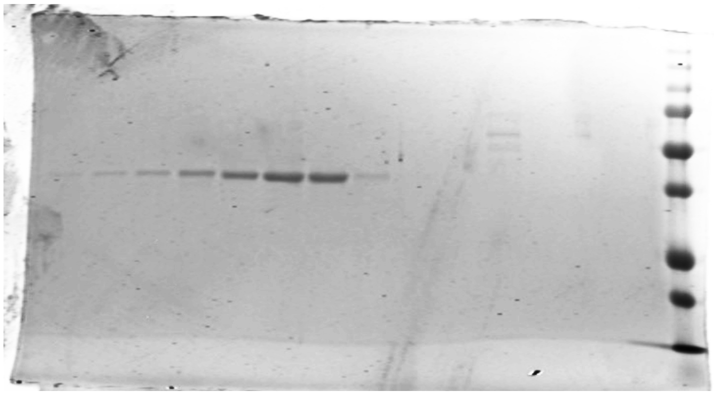

12. Any1-diUb purification.

(A) Synthesis of Any1-diUb

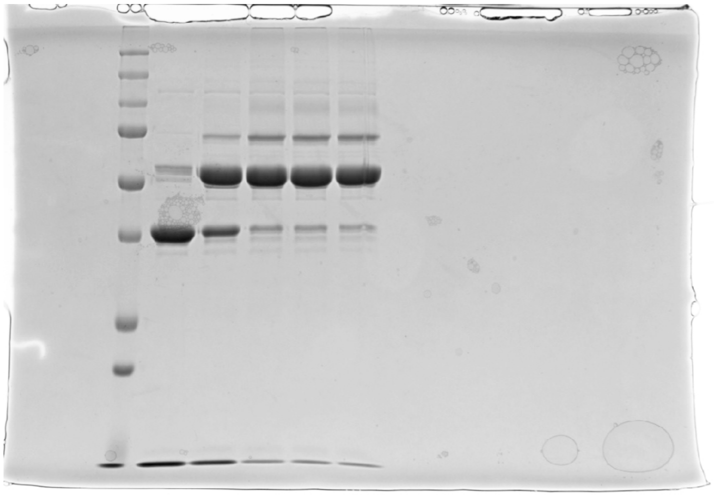

(B) Any1-diUb purification by S200 gel filtration.

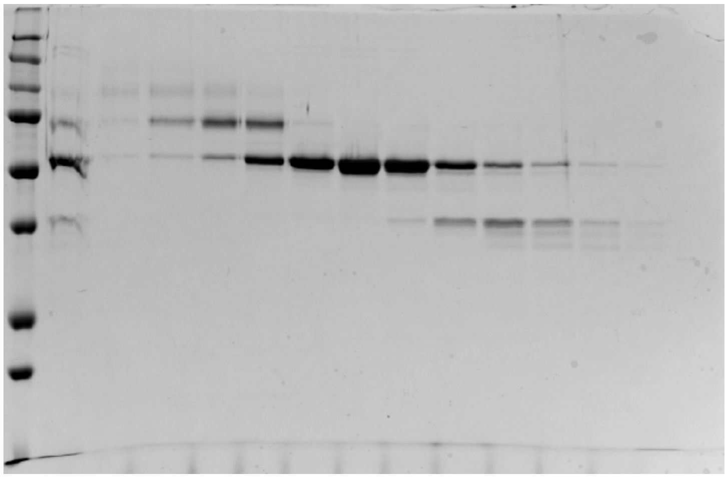

Supplement: Source data 2. [file elife-77424-data2.pdf]
